# Correcting the Bias Correction for the Bootstrap Confidence Interval in Mediation Analysis

**DOI:** 10.3389/fpsyg.2022.810258

**Published:** 2022-05-27

**Authors:** Tristan D. Tibbe, Amanda K. Montoya

**Affiliations:** Department of Psychology, University of California, Los Angeles, Los Angeles, CA, United States

**Keywords:** bias-corrected bootstrap confidence interval, indirect effect, bias correction, type I error rate, power, mediation, bootstrapping

## Abstract

The bias-corrected bootstrap confidence interval (BCBCI) was once the method of choice for conducting inference on the indirect effect in mediation analysis due to its high power in small samples, but now it is criticized by methodologists for its inflated type I error rates. In its place, the percentile bootstrap confidence interval (PBCI), which does not adjust for bias, is currently the recommended inferential method for indirect effects. This study proposes two alternative bias-corrected bootstrap methods for creating confidence intervals around the indirect effect: one originally used by Stine (1989) with the correlation coefficient, and a novel method that implements a reduced version of the BCBCI's bias correction. Using a Monte Carlo simulation, these methods were compared to the BCBCI, PBCI, and Chen and Fritz (2021)'s 30% Winsorized BCBCI. The results showed that the methods perform on a continuum, where the BCBCI has the best balance (i.e., having closest to an equal proportion of CIs falling above and below the true effect), highest power, and highest type I error rate; the PBCI has the worst balance, lowest power, and lowest type I error rate; and the alternative bias-corrected methods fall between these two methods on all three performance criteria. An extension of the original simulation that compared the bias-corrected methods to the PBCI after controlling for type I error rate inflation suggests that the increased power of these methods might only be due to their higher type I error rates. Thus, if control over the type I error rate is desired, the PBCI is still the recommended method for use with the indirect effect. Future research should examine the performance of these methods in the presence of missing data, confounding variables, and other real-world complications to enhance the generalizability of these results.

## 1. Introduction

Mediation analysis is a statistical method that researchers use to examine how one variable is able to influence another variable. It is a valuable tool in psychology research because it allows scientists to expose the mechanism(s) underlying psychological phenomena. As an example, Osberg and Eggert (2012) used mediation analysis to show that daily hassles increased bulimic symptoms in students by first increasing their irrational food beliefs. Thus, they were able to reveal not only that daily hassles affected students' bulimic symptoms, but also *how* they did so. It is important to note before continuing that mediation is concerned with causal processes, but a mediation analysis itself cannot be used as evidence that relationships are causal. As is the case with any statistical procedure, causation must be justified separately using research design, previous literature, and/or theory as supporting evidence.

Unlike a simple linear regression equation that estimates only the effect of a predictor variable *X* on an outcome variable *Y*, a **simple mediation model** (like the one used by Osberg and Eggert, 2012) involves a system of regression equations that estimates the **indirect effect** of a predictor *X* on an outcome *Y* through a single mediator variable *M*. In the example provided above, *X* would be daily hassles, *Y* would be bulimic symptoms, and *M* would be irrational food beliefs. The system of linear regression equations that constitutes a simple mediation model is as follows,


$$\begin{array}{r} Y_{i}=d_{1}+cX_{i}+e_{1i} \end{array}$$

(1) $$\begin{array}{r} M_{i}=d_{2}+aX_{i}+e_{2i} \end{array}$$

(2) $$\begin{array}{r} Y_{i}=d_{3}+c^{\prime }X_{i}+bM_{i}+e_{3i} \end{array}$$

where the *d*s represent the intercepts of the equations, the *e*s represent the error terms (assumed to be independent, normally distributed random variables with means of zero and a constant variance), and the subscript *i* indicates that the variable values belong to the *i*th individual. The equations above contain the parameters we estimate when we run a simple mediation analysis on a sample of data.

**Figure 1:**
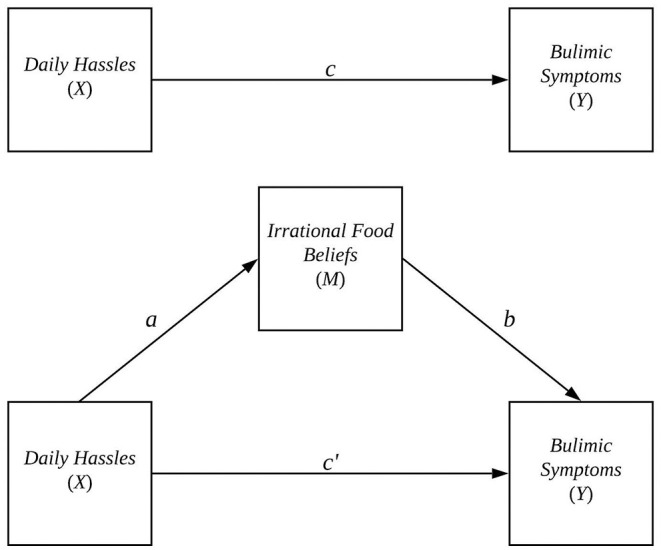
Simple mediation model used in (Osberg and Eggert, 2012).

To help visualize the relationship between *X*, *Y*, and *M*, see the path diagram in Figure 1. In the diagram, the effect of *X* on *M* is found by tracing the *a*-path from *X* to *M*, and the effect of *M* on *Y* controlling for *X* is found by tracing the *b*-path from *M* to *Y*. The indirect effect of *X* on *Y* through *M* is found by tracing from the *a*-path starting at *X* through the *b*-path ending at *Y*. Mathematically, the indirect effect is calculated by multiplying the *a* and *b* coefficients from Equations 1 and 2. Thus, in the context of the Osberg and Eggert (2012) study, *ab* would give the effect of daily hassles on students' bulimic symptoms through irrational food beliefs. In a sample, the true indirect effect *ab* is estimated by $â\hat{b}$, the product of the ordinary least squares estimates of *a* and *b*: â and $\hat{b}$, respectively.

To determine whether an indirect effect exists, either (a) a significance test can be performed to see if the null hypothesis *ab* = 0 can be rejected or (b) a confidence interval can be formed and examined to see if zero falls outside its confidence limits. However, even though both â and $\hat{b}$ are normally distributed under the assumptions of linear regression, their product is *not* normally distributed (Craig, 1936; Aroian, 1947; Aroian et al., 1978). As a result, the use of normal theory tests of statistical significance (and by extension normal theory confidence intervals) with the indirect effect have been shown to have lower power to detect a true effect than methods that do not assume normality (MacKinnon et al., 2002). Making matters more complicated is the fact that a closed-form solution for the indirect effect's sampling distribution has yet to be derived. Thus, no analytical approach exists for computing critical values for the distribution of the product (though some numerical approaches have been developed; e.g., see Tofighi and MacKinnon, 2011). An alternative approach is to use the observed data itself to empirically approximate the indirect effect's sampling distribution. The popular nonparametric bootstrap confidence interval, which one recent study found was applied to the indirect effect in over half of all mediation articles published in *Psychological Science* from 2011 to 2012 (Hayes and Scharkow, 2013), allows researchers to do exactly this.

Leveraging the power of modern computers, bootstrap procedures resample with replacement from the original sample of data many times (e.g., 5,000 times). Each of these 5,000 bootstrap samples is the same size as the original sample, and from each one a bootstrap indirect effect estimate, $â{\hat{b}}^{*}$, is computed. These 5,000 bootstrap indirect effect estimates form an observed bootstrap sampling distribution of the indirect effect. This observed bootstrap distribution containing 5,000 bootstrap estimates is a subset of the true bootstrap sampling distribution, which contains all possible bootstrap estimates calculated from bootstrap samples of the same size that can be drawn from the the original sample of data.

Based on the observed bootstrap sampling distribution, a bootstrap confidence interval for the indirect effect can be formed by first ordering the bootstrap estimates from smallest to largest (Efron and Tibshirani, 1993). Multiple methods can then be used to determine the endpoints of the bootstrap confidence interval. Of those available, two have been used the most in the mediation literature: the percentile bootstrap confidence interval (PBCI) and the bias-corrected bootstrap confidence interval (BCBCI).

## 2. Bootstrap Confidence Interval Techniques

**Figure 2:**
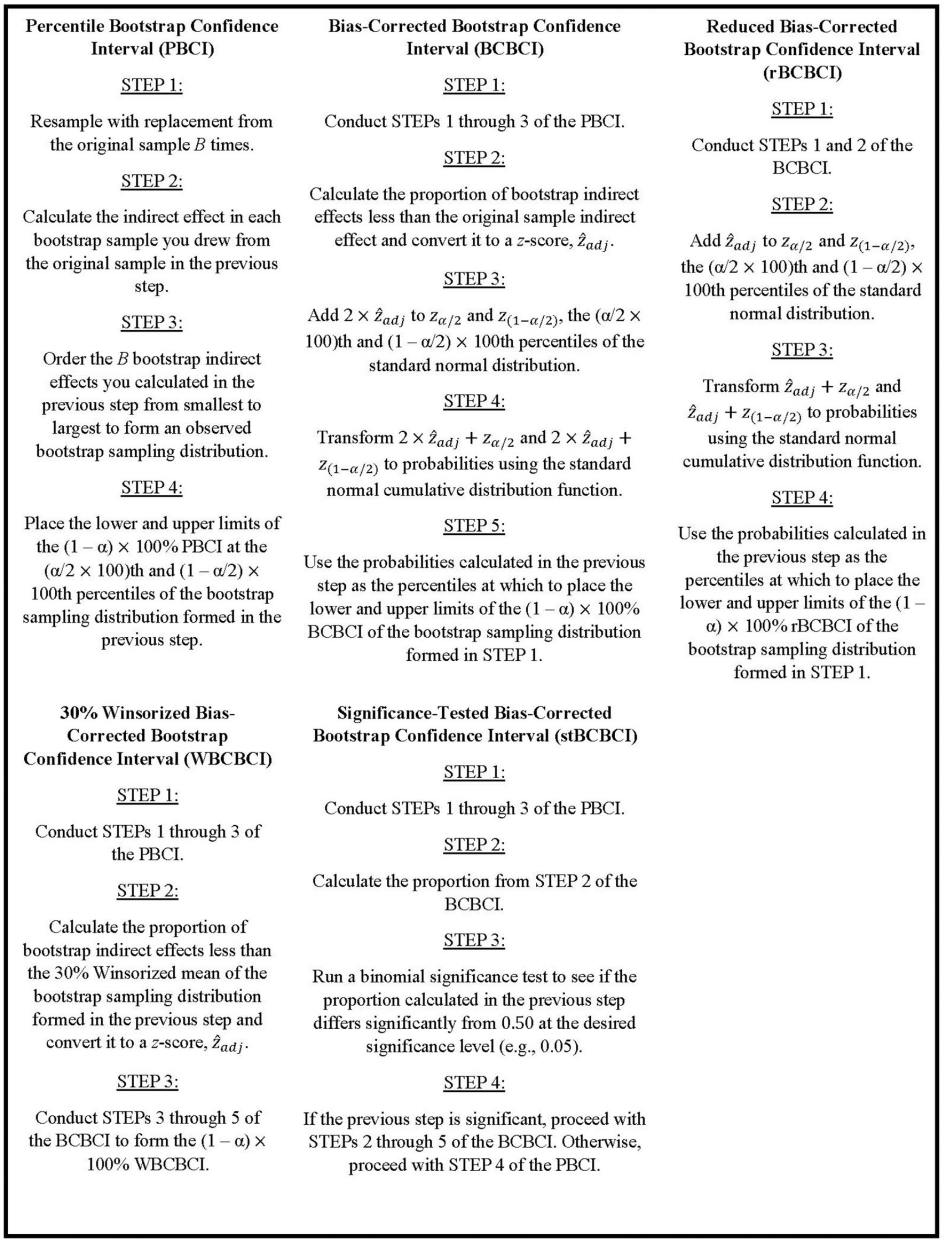
Steps to implement the bootstrap confidence interval methods.

The following two subsections detail the motivation and implementation of these two bootstrap confidence interval techniques, as well as describe type I error rate issues with the BCBCI in mediation analysis that motivate the current simulation study. The manuscript then transitions to discussing the primary goal of this research: developing alternative bias-corrected bootstrap techniques that address these issues with the traditional BCBCI when applied to the indirect effect. One alternative method was originally discussed in Stine (1989) and involves testing the significance of the bias the BCBCI is designed to correct, and the other was developed by the authors of this paper to implement a reduced bias correction that protects against overcorrecting. Explicit steps for implementing each bootstrap procedure discussed in this paper are written out in Figure 2.

### 2.1. Percentile Bootstrap Confidence Interval

After ordering the bootstrap indirect effect estimates from smallest to largest and determining the acceptable type I error rate for the analysis, α, the (α/2 × 100)th and (1−α/2) × 100th percentiles of the observed bootstrap sampling distribution are used to form the lower and upper limits of a (1−α) × 100% PBCI (Efron and Tibshirani, 1993).

Both the PBCI and the BCBCI rely on the assumption that some monotonic increasing function, *g*(·), exists to transform the sampling distributions of the sample indirect effect and its bootstrap estimator to a known symmetric distribution with variance $\sigma_{g\left(â\hat{b}\right)}^{2}$, the variance of $g\left(â\hat{b}\right)$ (Efron, 1982a; Stine, 1989). The normal distribution is usually chosen to be the known symmetric distribution—and it is used throughout the rest of this paper—because it is the asymptotic distribution of most statistics (Stine, 1989). The PBCI's and BCBCI's assumptions differ, however, in regards to the expected values of the sample and bootstrap indirect effects' transformed sampling distributions.

The (1−α) × 100% PBCI created from the true bootstrap sampling distribution—the bootstrap sampling distribution containing all possible bootstrap estimates calculated from bootstrap samples of the same size—has correct coverage (i.e., has α × 100% of the true sampling distribution of the indirect effect outside its limits) if the following holds true:


(3) $$\begin{array}{r} E\left(g\left(â\hat{b}\right)\right)=g\left(ab\right) \end{array}$$

(4) $$\begin{array}{r} E\left(g\left(â{\hat{b}}^{*}\right)\right)=g\left(â\hat{b}\right). \end{array}$$

That is, the expected value of the transformed sample indirect effect $g\left(â\hat{b}\right)$ has to be equal to the transformed true indirect effect *g*(*ab*), and the expected value of the transformed bootstrap indirect effect $g\left(â{\hat{b}}^{*}\right)$ has to be equal to the transformed sample indirect effect $g\left(â\hat{b}\right)$ (Stine, 1989). If either Equation 3 or Equation 4 does not hold true, there is mean bias present in either the transformed sample indirect effect's or the transformed bootstrap indirect effect's sampling distribution, and the PBCI is no longer guaranteed to have correct coverage. The BCBCI is a modification of the PBCI that weakens this requirement in an attempt to form confidence intervals guaranteed to have correct coverage even in the presence of bias.

### 2.2. Bias-Corrected Bootstrap Confidence Interval

In place of Equations 3 and 4, the BCBCI created using the true bootstrap sampling distribution has correct coverage if


(5) $$\begin{array}{r} E\left(g\left(â\hat{b}\right)\right)=g\left(ab\right)-z_{adj}\times \sigma_{g\left(â\hat{b}\right)} \end{array}$$

(6) $$\begin{array}{r} E\left(g\left(â{\hat{b}}^{*}\right)\right)=g\left(â\hat{b}\right)-z_{adj}\times \sigma_{g\left(â\hat{b}\right)} \end{array}$$

where *z*_*adj*_ is a constant and $\sigma_{g\left(â\hat{b}\right)}$ is the standard error of $g\left(â\hat{b}\right)$ (Stine, 1989). As before with the PBCI, these transformed terms take $â\hat{b}$ and $â{\hat{b}}^{*}$ and reshape their sampling distributions so that they both have a known, symmetric sampling distribution (i.e., the normal distribution). The BCBCI then goes a step further than the PBCI by allowing the transformed sample and bootstrap estimators to have mean bias of the form $-z_{adj}\times \sigma_{g\left(â\hat{b}\right)}$. This means that as the variability of the estimate decreases (i.e., as $\sigma_{g\left(â\hat{b}\right)}$ gets closer and closer to zero, which occurs as the sample size gets larger and larger), so too does the mean bias of these transformed estimators.

Even if the transformed estimators are mean biased, however, this does not imply that there is mean bias of the untransformed sample indirect effect (**sample mean bias**) or mean bias of the untransformed bootstrap indirect effect (**bootstrap mean bias**). This is due to the fact that *g*(·) does not have to be a linear transformation, only monotonically increasing, and bias of the mean is not preserved under nonlinear transformations (e.g., Needham, 1993). Thus, the expected value of a statistic can be equal to the parameter it estimates, but this does not imply the expected value of a nonlinear transformation of that statistic will be equal to the nonlinear transformed value of its parameter. A good example of this is the sample variance, *s*^2^: Although *s*^2^ is a mean unbiased estimator of the population variance σ^2^ (i.e., *E*(*s*^2^) = σ^2^), the square root of *s*^2^ = *g*(*s*^2^) = *s*—which is a nonlinear, monotonic increasing transformation of *s*^2^—is a mean biased estimator of the population standard deviation (i.e., *E*(*s*)≠σ). Similarly, $g\left(â\hat{b}\right)$ and $g\left(â{\hat{b}}^{*}\right)$ could be mean biased even if $â\hat{b}$ and $â{\hat{b}}^{*}$ are not, and vice versa. This is important, because it means that the assumptions underlying the BCBCI do not depend on sample mean bias or bootstrap mean bias, and so the BCBCI's bias correction does not target them.

Unlike bias of the mean, bias of the median *is* preserved under nonlinear transformations (e.g., Brown, 1947), and because *g*(·) transforms to a symmetric distribution, the mean of the transformed distribution is equal to its median (i.e., $E\left(g\left(â\hat{b}\right)\right)=Med\left(g\left(â\hat{b}\right)\right)$ and $E\left(g\left(â{\hat{b}}^{*}\right)\right)=Med\left(g\left(â{\hat{b}}^{*}\right)\right)$). Thus, Equations 5 and 6 imply that


(7) $$\begin{array}{r} Med\left(g\left(â\hat{b}\right)\right)=g\left(ab\right)-z_{adj}\times \sigma_{g\left(â\hat{b}\right)} \end{array}$$

(8) $$\begin{array}{r} Med\left(g\left(â{\hat{b}}^{*}\right)\right)=g\left(â\hat{b}\right)-z_{adj}\times \sigma_{g\left(â\hat{b}\right)} \end{array}$$

where *Med*(·) signifies the median of the variable in parentheses. Using the fact that bias of the median is preserved under nonlinear transformations, then, we find that Equations 7 and 8 imply that


(9) $$\begin{array}{r} Med\left(â\hat{b}\right)=g^{-1}\left(g\left(ab\right)-z_{adj}\times \sigma_{g\left(â\hat{b}\right)}\right) \end{array}$$

(10) $$\begin{array}{r} Med\left(â{\hat{b}}^{*}\right)=g^{-1}\left(g\left(â\hat{b}\right)-z_{adj}\times \sigma_{g\left(â\hat{b}\right)}\right) \end{array}$$

(see Efron, 1979). If *z*_*adj*_ = 0 in Equations 9 and 10, the $z_{adj}\times \sigma_{g\left(â\hat{b}\right)}$ terms drop out and so *g*^−1^(·) and *g*(·) cancel, leaving *ab* as the median of $â\hat{b}$'s sampling distribution and $â\hat{b}$ as the median of $â{\hat{b}}^{*}$'s bootstrap sampling distribution. The farther from zero *z*_*adj*_ is, the farther the medians of the untransformed sampling distributions are from the corresponding true parameter values. Thus, although *z*_*adj*_ is on the scale of the transformed estimators, it is an indicator of the median bias present in the untransformed sampling distributions of the sample and bootstrap indirect effects.

The bias constant *z*_*adj*_ is found using the equation


$$\begin{array}{l} z_{adj}=\Phi^{-1}\left(G^{*}\left(â\hat{b}\right)\right) \end{array}$$

where Φ^−1^ is the inverse of the normal cumulative distribution function and $G^{*}\left(â\hat{b}\right)$ is the cumulative distribution function of the true bootstrap sampling distribution evaluated at the sample indirect effect (Stine, 1989). In other words, *z*_*adj*_ is the *z*-score corresponding to the probability that a bootstrap estimate randomly selected from the true bootstrap sampling distribution of the indirect effect will be less than or equal to the original sample estimate. If this probability is 0.50, and thus the original estimate is the median of the true bootstrap sampling distribution, the corresponding *z*-score will be zero and so the bias terms in Equations (5–10) will be equal to zero as well.

In practice, G^*^ is unknown, and so *z*_*adj*_ is estimated using


(11) $$\begin{array}{r} {ẑ}_{adj}=\Phi^{-1}\left(\frac{\#\left\{â{\hat{b}}^{*}<â\hat{b}\right\}}{B}\right) \end{array}$$

where $\#\left\{â{\hat{b}}^{*}<â\hat{b}\right\}$ is the number of bootstrap indirect effect estimates in the observed bootstrap sampling distribution that are less than the original sample estimate and *B* is the total number of bootstrap indirect effect estimates collected (e.g., 5,000; Efron and Tibshirani, 1993). Thus, ẑ_*adj*_ is the *z*-score corresponding to the proportion of bootstrap estimates less than the original sample estimate in the observed bootstrap sampling distribution.

A (1−α) × 100% BCBCI is formed by first calculating the *z*-scores corresponding to the percentiles at which to place the confidence interval's lower and upper limits using the following equations:


(12) $$\begin{array}{r} \text{Lower:}2\times {ẑ}_{adj}+z_{\alpha /2} \end{array}$$

(13) $$\begin{array}{r} \text{Upper:}2\times {ẑ}_{adj}+z_{\left(1-\alpha /2\right)} \end{array}$$

where *z*_α/2_ is the *z*-score corresponding to the (α/2 × 100)th percentile of the standard normal distribution and *z*_(1−α/2)_ is the *z*-score corresponding to the (1−α/2) × 100th percentile of the standard normal distribution (Efron and Tibshirani, 1993). The ẑ_*adj*_ term is added to both *z*-scores twice: once to account for the estimated median bias of the sample indirect effect and once to account for the estimated median bias of the bootstrap indirect effect.

The final step in forming the BCBCI is converting the *z*-scores from Equations 12 and 13 to the percentiles of the observed bootstrap sampling distribution at which to place the lower and upper confidence limits using the following equations:


(14) $$\begin{array}{r} \text{Lower:}\Phi \left(2\times {ẑ}_{adj}+z_{\alpha /2}\right) \end{array}$$

(15) $$\begin{array}{r} \text{Upper:}\Phi \left(2\times {ẑ}_{adj}+z_{\left(1-\alpha /2\right)}\right) \end{array}$$

where Φ is the normal cumulative distribution function. Note that if ẑ_*adj*_ = 0, Equations 14 and 15 will give the (α/2 × 100)th and (1−α/2) × 100th percentiles of the observed bootstrap sampling distribution, resulting in a (1−α) × 100% BCBCI that is equal to the corresponding (1−α) × 100% PBCI. This is because, based on the form of the bias assumed through Equations 9 and 10, the BCBCI attempts to recenter the PBCI around the true indirect effect *ab*. When ẑ_*adj*_ = 0, we estimate that there is no median bias between either the bootstrap indirect effect and $â\hat{b}$ or between the sample indirect effect and *ab*, and so the PBCI is assumed to be already well-centered around the true effect. By allowing ẑ_*adj*_ to be different from zero, the BCBCI is designed to have less strict assumptions than the PBCI, and thus be appropriate in more situations.

Although the BCBCI is supposed to be more flexible than the PBCI, there is a growing body of research indicating that confidence intervals for the indirect effect created by the BCBCI have inflated type I error rates (i.e., exclude a true indirect effect of zero more often then they should) compared to the PBCI and other confidence interval methods, particularly with smaller sample sizes (*n* <500) (MacKinnon et al., 2004; Biesanz et al., 2010; Fritz et al., 2012; Chen and Fritz, 2021). Fritz et al. (2012) found that increasing the number of bootstrap samples used to create the BCBCI had no effect on these elevated type I error rates. The issue is perpetuated by the BCBCI's continued popularity in published mediation articles: Götz et al. (2021) collected all mediation articles that applied resampling techniques to the indirect effect published from 2018 to 2019 in five of the American Psychological Association's most prominent journals and found that about 25% still used the BCBCI in at least one mediation analysis.

Researchers prefer the BCBCI to competing inferential methods because of its higher power to detect a true indirect effect (MacKinnon et al., 2004; Preacher and Hayes, 2008; Williams and MacKinnon, 2008). It may offer advantages in terms of balance too. Balance refers to how evenly confidence intervals fall below and above the true effect. For example, we would expect a 95% confidence interval to capture the true effect 95% of the time and exclude the true effect 5% of the time (meaning it has correct coverage). If the confidence interval were perfectly balanced as well, it would fall below the true effect 2.5% of the time and it would fall above the true effect 2.5% of the time. Previous research has found evidence that the BCBCI may offer better balance than the PBCI with nonzero indirect effects (Williams and MacKinnon, 2008). Although it was also found that the PBCI may be more balanced when the indirect effect is equal to zero, an examination of the tabled data from Williams and MacKinnon (2008) revealed that the performance of these two methods were quite similar overall. Additionally, a later study by Fritz et al. (2012) failed to find a significant difference in the balance of the PBCI and BCBCI. Still more recent, Supplementary Material from Chen and Fritz (2021) suggests that the BCBCI may offer better overall balance across both zero and nonzero indirect effects. Clearly, further comparison of the balance of these two methods is warranted, and it would be beneficial to find an alternative to the BCBCI with good balance that maintains its high power while also controlling type I error rates for the indirect effect.

Chen and Fritz (2021) attempted to identify such an alternative in a recent simulation study. They proposed several bias-corrected bootstrap methods that replaced $â\hat{b}$ in Equation 11 with different measures of central tendency, including the Winsorized mean of the observed bootstrap sampling distribution set at different percentages of Winsorization. For example, their 30% Winsorized bias-corrected bootstrap confidence interval (WBCBCI) involved first finding the 30% Winsorized mean of the observed bootstrap sampling distribution of the indirect effect, and then calculating the bias correction by replacing $â\hat{b}$ with the Winsorized mean in Equation 11. Equations 12 through 15 were then used to calculate the bias-corrected confidence interval just as they were for the BCBCI. Ultimately, Chen and Fritz (2021) found that there was always a tradeoff between type I error rate and power, with power and type I error rate both decreasing as percentage of Winsorization increased. No method exceeded the BCBCI in terms of power, no method had better control over the type I error rate than the PBCI (which is equivalent to a 50% Winsorized bias-corrected bootstrap confidence interval), and all methods had balance levels that fell between those of the BCBCI and the PBCI (the BCBCI with the best balance overall and PBCI with the worst).

Instead of changing the measure of central tendency used in the BCBCI's bias estimation as Chen and Fritz (2021) did, the present study proposes two bootstrap confidence interval alternatives to the BCBCI based on the assumptions made to appropriately apply the BCBCI to the indirect effect: one introduced by Stine (1989) adapted here for use with the indirect effect, and another original method developed by the authors of this paper. The performance of these methods are then compared to the BCBCI, the PBCI, and the WBCBCI proposed by Chen and Fritz (2021) in a simulation study.

## 3. Alternatives to the BCBCI for the Indirect Effect

The alternative bias-corrected bootstrap methods discussed in this section target assumptions underlying the BCBCI's correction that may prove fallible when it is applied to the indirect effect. The first method, the significance-tested bias-corrected bootstrap confidence interval (stBCBCI), implements a significance test to determine whether differences observed between the sample indirect effect and the median of the observed bootstrap sampling distribution are large enough to warrant the use of a bias correction. The second method, the reduced bias-corrected bootstrap confidence interval (rBCBCI), applies a smaller bias correction than the BCBCI to decrease the chance of overcorrecting the confidence limits of the indirect effect's confidence interval.

### 3.1. Significance-Tested Bias-Corrected Bootstrap Confidence Interval

Recall from Section 2.2 that ẑ_*adj*_ determines how large of a bias correction the BCBCI applies to the confidence limits of the PBCI. This is because ẑ_*adj*_ represents the bias present between the median bootstrap indirect effect and the sample indirect effect, and through Equations (9 and 10) it is assumed that the bias present between the median sample indirect effect and the true indirect effect takes the same form. Thus, the larger ẑ_*adj*_ is, the farther *ab* is assumed to be from the center of the PBCI and the more its confidence limits need to be shifted to recenter the interval around the true effect as a result. Still, ẑ_*adj*_ is only an estimate of *z*_*adj*_, which can only be obtained using the true bootstrap sampling distribution that contains all possible bootstrap estimates calculated from bootstrap samples of the same size. Thus, it is always possible when *z*_*adj*_ = 0 (meaning that no bias correction is needed) to still observe a nonzero value of ẑ_*adj*_ in a sample due to random variability. If *z*_*adj*_ = 0 and ẑ_*adj*_ is nonzero due solely to random error, the BCBCI will inappropriately correct for bias that does not actually exist. The result is a BCBCI that inaccurately moves a PBCI that was already well-centered around the true indirect effect, moving one of the two confidence limits closer to excluding the true effect. This scenario can occur when the true indirect effect is zero because the underlying sampling distribution of the sample indirect effect is symmetric around *ab* = 0 (Craig, 1936). Thus, *ab* is the median of the sampling distribution and *z*_*adj*_ = 0 because there is no median bias. If we still obtain a nonzero ẑ_*adj*_ value from a sample, then the PBCI's limits will be incorrectly shifted and the true zero indirect effect will be closer to one of the two limits, resulting in a greater chance of the BCBCI excluding the true parameter value of zero and committing a type I error than the unadjusted PBCI.

To protect against such scenarios as the one described above, Stine (1989) recommended performing a test before applying the BCBCI's bias correction to the confidence limits to ensure the median bias in the observed bootstrap sampling distribution was statistically significant, rather than just due to random variability. In his method, the outcome of a binomial test with null hypothesis


$$\begin{array}{r} H_{0}:G^{*}\left(â\hat{b}\right)=0.5 \end{array}$$

and two-tailed alternative hypothesis


$$\begin{array}{r} H_{a}:G^{*}\left(â\hat{b}\right)\neq 0.5 \end{array}$$

determines what bootstrap confidence interval method to employ. For this procedure, the proportion of bootstrap indirect effect estimates less than the original sample estimate in the observed bootstrap sampling distribution (from Equation 11) is used to estimate $G^{*}\left(â\hat{b}\right)$. If the proportion is significantly different from .50 at an α-level of .05 (which, with 5,000 bootstrap replications, equates to a proportion less than .4862 or a proportion greater than .5138), the BCBCI is implemented. On the other hand, if the test is not significant, no bias correction is applied to the confidence limits because it cannot be concluded that $â\hat{b}$ differs from the median of the true bootstrap sampling distribution. Thus, with a nonsignificant test, the PBCI is used instead of the BCBCI. The α-level used to determine the significance of the median bias here can be different from the α-level used to determine the confidence level of the bootstrap confidence intervals. Stine (1989) applied this stBCBCI procedure to the correlation coefficient. The current study is the first to apply it to the indirect effect.

### 3.2. Reduced Bias-Corrected Bootstrap Confidence Interval

While the stBCBCI is meant to prevent the use of the BCBCI in cases where no actual median bias is present to decrease its type I error rate—i.e., the rate at which it indicates *ab* is significantly different from zero when it is in fact equal to zero—, perhaps reducing the BCBCI's bias correction can have the same effect. Recall from Section 2.2 that the BCBCI adds ẑ_*adj*_ to the *z*-scores corresponding to the percentiles of the lower and upper confidence limits of the PBCI *twice*: once to correct for the bias given in Equation 9 and once for the bias given in Equation 10. This form of bias is only an assumption, however, and if it does not hold true with the indirect effect then the BCBCI improperly adjusts for bias. Examining the properties of the indirect effect's sampling distribution may thus inform a bias correction that is better suited for this statistic.

As stated in the previous section, when the true indirect effect is zero, the sampling distribution of the indirect effect is known to be symmetric about the origin, and so its median which is equal to its mean is equal to the true indirect effect (Craig, 1936). As a result, there is no median bias when *ab* = 0 and so no bias correction is needed. A bias correction is thus only necessary when the indirect effect's distribution is skewed at nonzero values of *ab*. In practice, we can never know when the true indirect effect is zero and no bias correction should be implemented, and so perhaps reducing the bias correction implemented by the BCBCI will offer a method that can still correct for bias when *ab* is nonzero while simultaneously decreasing the chances of committing a type I error when *ab* is zero. To this end, we propose the following modifications to Equations (14 and 15):


$$\begin{array}{r} \text{Lower:}\Phi \left({ẑ}_{adj}+z_{\alpha /2}\right)\\ \text{Upper:}\Phi \left({ẑ}_{adj}+z_{\left(1-\alpha /2\right)}\right). \end{array}$$

Thus, ẑ_*adj*_ is added to the *z*-scores corresponding to the percentiles of the lower and upper confidence limits of the PBCI once instead of twice. This translates to applying the BCBCI's bias correction at only the bootstrap indirect effect level (and not the sample indirect effect level), reducing how much the PBCI's limits are shifted and theoretically recentering the PBCI around $â\hat{b}$ as a result. These modified limits form the new (1−α) × 100% rBCBCI for the indirect effect.

The following section describes a simulation study that compared the stBCBCI and the rBCBCI to the PBCI, the BCBCI, and the 30% Winsorized bias-corrected bootstrap confidence interval (WBCBCI) proposed by Chen and Fritz (2021).

## 4. Simulation

### 4.1. Manipulated Factors

To compare the performance of the five bootstrap confidence interval methods listed above, a Monte Carlo simulation was conducted. In addition to the bootstrap method used, three other factors were varied in the simulation: effect size of the *a*-path, effect size of the *b*-path, and sample size. First, to see how the bootstrap methods performed in the presence of a variety of different effect sizes, the sizes of the *a*- and *b*-paths were varied across conditions to be 0.00 (null effect), 0.16 (small effect), 0.39 (medium effect), and 0.59 (large effect) in accordance with effect size conventions established by Cohen (1988). These four different *a*-path sizes and four different *b*-path sizes then multiplied to form a total of 16 different path-size combinations.

Second, since previous studies have shown that the BCBCI has inflated type I error rates with samples of *n* <500, the bootstrap methods were tested with five different sample sizes: 25, 50, 75, 100, and 500. This resulted in a total of 80 conditions. Each condition was run 1,000 times, resulting in a total of 80,000 iterations run in the simulation.

In addition to the manipulated factors described above, the confidence levels of the bootstrap methods were varied to be 95, 90, and 80%. However, as was found in MacKinnon et al. (2004), results were similar regardless of the confidence level, so the 95% setting is focused on throughout the rest of this paper. Results from the other two settings are available in the Supplementary Material.

### 4.2. Data Generation

The Monte Carlo simulation was coded and run in R Version 4.0.2 (R Core Team, 2020). For each iteration, values of *X* were randomly generated from a standard normal distribution, with the number of *X* values drawn determined by the sample size factor. These *X* values were then plugged into Equation 1 and summed with error terms randomly drawn from the standard normal distribution to generate corresponding values of *M*. The intercept of the equation was set to zero, and the *a*-path was set to one of the four effect size levels listed above. After generating values of *M* using Equation 1, Equation 2 was used with the *M* and *X* values to generate the corresponding *Y* values. Once again, the equation's error term was added by randomly selecting a value from the standard normal distribution, and the intercept was again set to zero. The size of the *b*-path was set to one of the four effect size levels as determined by the current condition, and the *c*′-path was set to zero since this path has not been found to have any effect on bootstrap confidence interval approaches for the indirect effect (MacKinnon et al., 2004; Fritz and MacKinnon, 2007).

Once values for *X*, *M*, and *Y* were obtained, ordinary least squares regression was applied to estimate *a* and *b* in Equations (2 and 3), respectively. These estimates were then multiplied together to get $â\hat{b}$, the original sample estimate of the indirect effect. The simulation then drew samples of the same size as the original sample with replacement from the generated *X*, *M*, and *Y* values 5,000 times, calculating the indirect effect in each resample to form 5,000 bootstrap estimates of the indirect effect. These estimates were then ordered from smallest to largest, and the PBCI, BCBCI, stBCBCI, rBCBCI, and WBCBCI procedures were used to calculate 95% confidence intervals for the indirect effect to compare the methods in terms of type I error rate, power, balance, coverage, and width.

### 4.3. Measured Outcomes

For each condition, **sample mean bias** was calculated as


$$\begin{array}{r} \frac{\sum_{j=1}^{1000}â{\hat{b}}_{j}}{1000}-ab, \end{array}$$

where $â{\hat{b}}_{j}$ is the sample indirect effect estimate in iteration *j* and 1,000 is the total number of iterations. Also, **bootstrap mean bias** was calculated for each condition as


$$\begin{array}{r} \frac{\sum_{j=1}^{1000}\left(\frac{\sum_{k=1}^{5000}â{\hat{b}}_{kj}^{*}}{5000}-â{\hat{b}}_{j}\right)}{1000}, \end{array}$$

where $â{\hat{b}}_{kj}^{*}$ is the bootstrap indirect effect estimate from bootstrap sample *k* in iteration *j*, and 5,000 is the total number of bootstrap indirect effect samples drawn in iteration *j*. These measures were collected to monitor mean bias and illustrate empirically whether the BCBCI's bias correction is based on the mean bias present in either the bootstrap indirect effect's or sample indirect effect's sampling distribution. The results should show that the bias correction does not depend on these mean biases. Instead, the correction targets median bias in the estimators' sampling distributions.

The performance of the methods included in this simulation study are discussed using the type I error rates, power, and balance of the bootstrap confidence intervals. In the seven conditions where either the *a*-path, the *b*-path, or both were equal to zero (meaning the true indirect effect was zero), the number of times a confidence interval excluded zero was tallied up and divided by the total number of iterations to get the proportion of times zero was excluded by each method (i.e., the method's **type I error rate**). The sizes of the type I error rates were assessed using Bradley's liberal robustness criterion which, with α = 0.05, resulted in an interval from 0.025 to 0.075 (Bradley, 1978). If the type I error rate of a confidence interval fell outside of this interval, it was deemed to have either an inflated (if it fell above the interval) or a conservative (if it fell below the interval) type I error rate.

The **power** of each method was calculated exactly the same way as the type I error rate except in the conditions where both the *a*-path and the *b*-path were nonzero. To find which manipulated factors had a significant impact on rejection rate (i.e., type I error rate and power), logistic regression analyses were run with the binary 0/1 indicator variable from the simulation (0 indicates zero is included in the confidence interval, 1 indicates zero is excluded) entered as an outcome and sample size, *a*-path size, *b*-path size, and method entered as factors. Type II sums of squares were used to test the significance of the main effects of the factors and all possible two-way through four-way interactions. To protect against type I error rate inflation, only effects with *p*-values less than .001 were deemed significant.

For each bootstrap method within each condition, **balance** was calculated by recording the number of times the confidence interval was either above or below the true indirect effect (i.e., did not capture the true indirect effect). These tallies were then counted across iterations to give the total number of confidence intervals that fell above the true effect and the total number that fell below the true effect in each condition. Finally, the total number of confidence intervals that fell above the true effect was divided by the total number of confidence intervals that excluded the true effect (i.e., the number above the true effect plus the number below it). If this value was equal to 0.50, the method was perfectly balanced, and balance decreased as the value's distance from 0.50 increased. Values greater than 0.50 indicated that the confidence interval fell above the true effect more often than it fell below, and values smaller than 0.50 indicated the confidence interval fell below the true effect more often than it fell above. A binomial significance test was also applied to the balance values to see which were significantly different from 0.50 at an α-level of 0.05.

In addition to the aforementioned outcome measures, confidence interval coverage (the percentage of the time a confidence interval captures the true effect) and width (the upper confidence limit minus the lower confidence limit) were also collected for each method. To save space, these results are included in the Supplementary Material.

## 5. Results

The following subsections summarize the results of the simulation study in terms of the measured outcome variables using a series of tables and figures. The mean bias, type I error rate, and balance figures each contain multiple graphs, with each graph pertaining to an *a*-path-*b*-path combination. Within each graph, the outcome variable is plotted on the *y*-axis and the natural logarithm of the sample size is plotted on the *x*-axis. The power figures, on the other hand, contain multiple graphs that each pertain to a *b*-path size, and the *a*-path sizes are plotted along the *x*-axis. There are separate figures for the sample sizes of 25, 75, and 500 included in the paper, and additional figures are available in the Supplementary Material. The power graphs were structured in this way to make it easier to detect differences in the power of the methods. The outcome variables are discussed in the following order: mean bias, type I error rate, power, and balance.

### 5.1. Mean Bias

**Figure 3:**
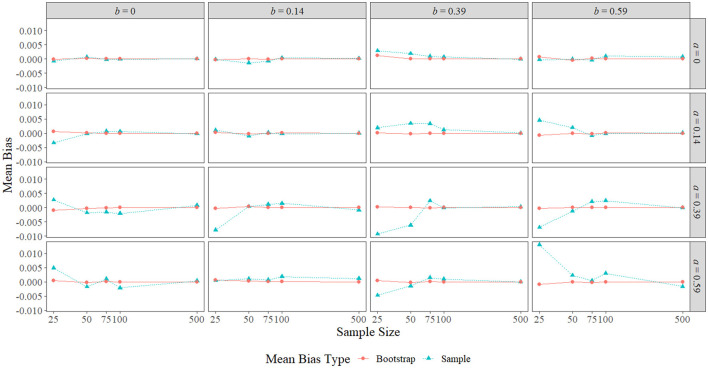
Sample mean bias and bootstrap mean bias across the range of *a*-path sizes, *b*-path sizes, and sample sizes. The *x*-axis is on the natural log scale.

Figure 3 displays the sample mean bias and bootstrap mean bias in each condition. The largest sample mean biases were observed with a sample size of 25 and a nonzero true indirect effect, with the largest positive mean bias of 0.013 obtained when *a* = *b* = 0.59, and the largest negative mean bias of −0.009 obtained when *a* = *b* = 0.39. With nonzero true indirect effects, sample mean bias appeared to be the worst at the smallest two or three sample sizes when the size of one or both the paths was larger (0.39 or 0.59). A notable exception to this pattern occurred when *a* = 0.59 and *b* = 0.14, where the sample mean bias remained close to zero across all sample sizes. When *a* = 0, the sample mean bias remained near zero regardless of the size of *b* or the sample size, and when *b* = 0 and the sample size was 25, the sample mean bias grew as the size of *a* increased. Overall, the sample mean bias shrank toward zero in all effect size conditions as sample size increased. Bootstrap mean bias, on the other hand, remained near zero across all conditions.

### 5.2. Type I Error Rate

**Figure 4:**
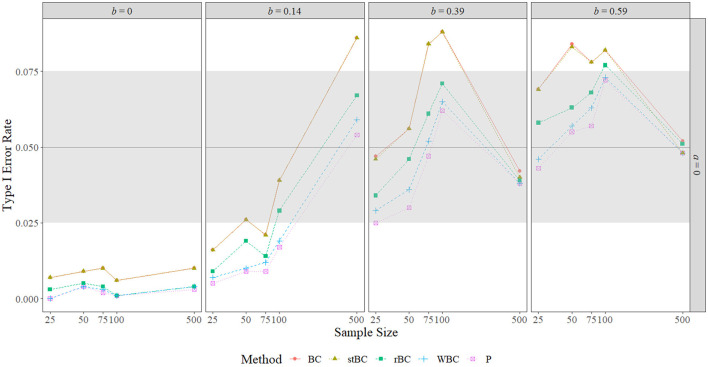
Type I error rate of all methods when *a*-path is zero across the range of *b*-path sizes and sample sizes. BC, bias-corrected bootstrap confidence interval; stBC, significance-tested bias-corrected bootstrap confidence interval; rBC, reduced bias-corrected bootstrap confidence interval; WBC, 30% Winsorized bias-corrected bootstrap confidence interval; P, percentile bootstrap confidence interval. The black horizontal line at .05 on the graphs represents the target type I error rate determined by the α-level of .05, and the grey shaded region indicates Bradley's liberal robustness criterion (.025 to .075). The *x*-axis is on the natural log scale.

**Figure 5:**
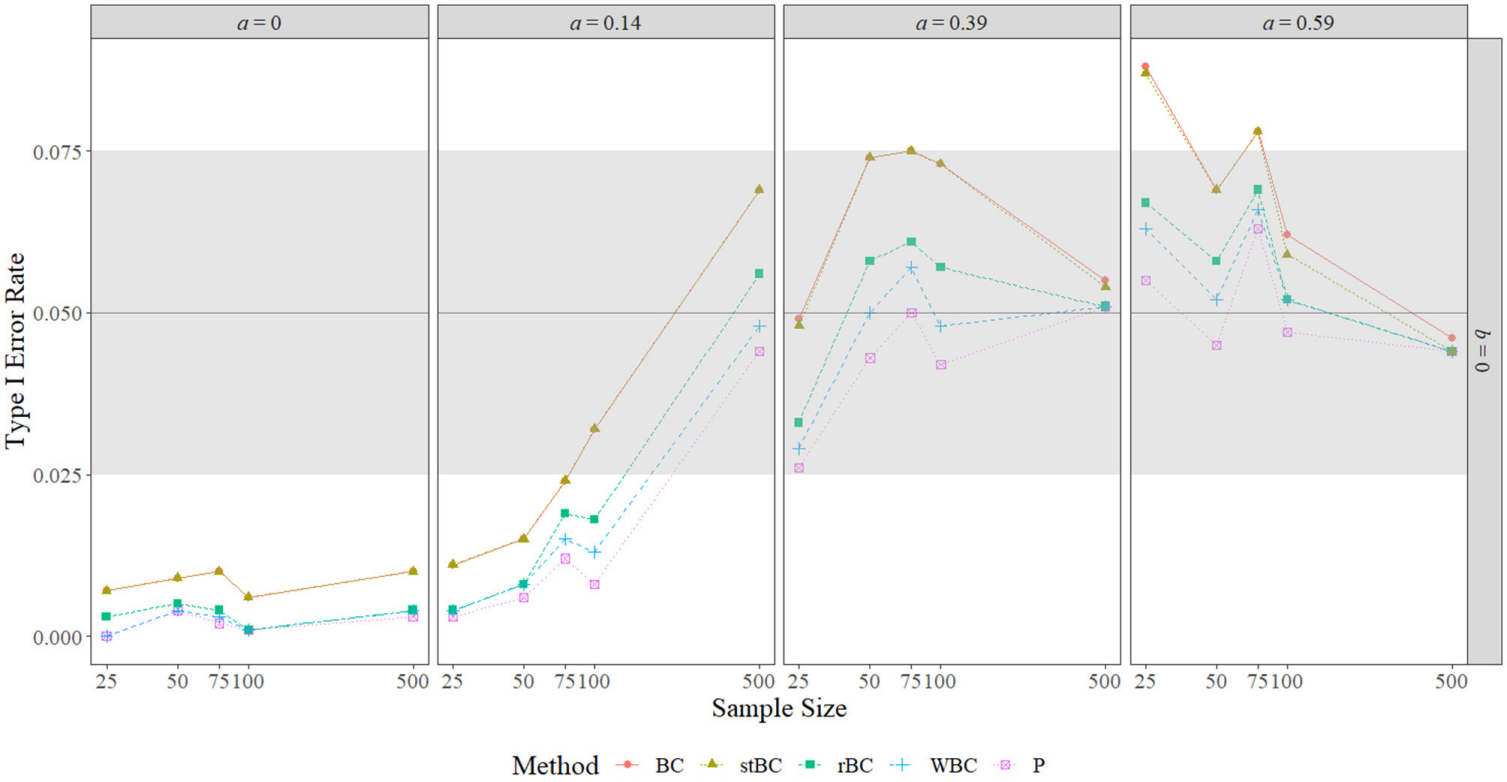
Type I error rate of all methods when *b*-path is zero across the range of *a*-path sizes and sample sizes. BC, bias-corrected bootstrap confidence interval; stBC, significance-tested bias-corrected bootstrap confidence interval; rBC, reduced bias-corrected bootstrap confidence interval; WBC = 30% Winsorized bias-corrected bootstrap confidence interval; P, percentile bootstrap confidence interval. The black horizontal line at .05 on the graphs represents the target type I error rate determined by the α-level of .05, and the grey shaded region indicates Bradley's liberal robustness criterion (.025 to .075). The *x*-axis is on the natural log scale.

Figures 4 and 5 display the type I error rates of the methods when *a* = 0 and *b* = 0, respectively, and the type I error rates found in all conditions are included in Table 1. The black horizontal line at 0.05 on the graphs represents the target type I error rate determined by the α-level of 0.05, and the gray shaded region indicates Bradley's liberal robustness criterion. The first notable feature of these graphs is that, regardless of condition, the order of the methods in terms of type I error rate almost always remained the same, with the BCBCI having the highest type I error rate followed by the stBCBCI, the rBCBCI, the WBCBCI, and finally the PBCI with the lowest type I error rate. The only deviation from this order occurred when *b* = 0.59 and the sample size was set at 500. In this condition, the rBCBCI's type I error rate of 0.051 exceeded the stBCBCI's type I error rate of 0.048.

**Table 1:** Type I error rate of all methods across all conditions where *ab* = 0.

		**Bootstrap Method**				

**a**	**b**	**Sample Size**	***P***	**BC**	**rBC**	**WBC**	**stBC**
0	0	25	0.000	0.007	0.003	0.000	0.007
		50	0.004	0.009	0.005	0.004	0.009
		75	0.002	0.010	0.004	0.003	0.010
		100	0.001	0.006	0.001	0.001	0.006
		500	0.003	0.010	0.004	0.004	0.010
0	0.14	25	0.005	0.016	0.009	0.007	0.016
		50	0.009	0.026	0.019	0.010	0.026
		75	0.009	0.021	0.014	0.012	0.021
		100	0.017	0.039	0.029	0.019	0.039
		500	0.054	0.086	0.067	0.059	0.086
0	0.39	25	0.025	0.047	0.034	0.029	0.046
		50	0.030	0.056	0.046	0.036	0.056
		75	0.047	0.084	0.061	0.052	0.084
		100	0.062	0.088	0.071	0.065	0.088
		500	0.038	0.042	0.039	0.038	0.040
0	0.59	25	0.043	0.069	0.058	0.046	0.069
		50	0.055	0.084	0.063	0.057	0.083
		75	0.057	0.078	0.068	0.063	0.078
		100	0.072	0.082	0.077	0.073	0.082
		500	0.048	0.052	0.051	0.048	0.048
0.14	0	25	0.003	0.011	0.004	0.004	0.011
		50	0.006	0.015	0.008	0.008	0.015
		75	0.012	0.024	0.019	0.015	0.024
		100	0.008	0.032	0.018	0.013	0.032
		500	0.044	0.069	0.056	0.048	0.069
0.39	0	25	0.026	0.049	0.033	0.029	0.048
		50	0.043	0.074	0.058	0.050	0.074
		75	0.050	0.075	0.061	0.057	0.075
		100	0.042	0.073	0.057	0.048	0.073
		500	0.051	0.055	0.051	0.051	0.054
0.59	0	25	0.055	0.088	0.067	0.063	0.087
		50	0.045	0.069	0.058	0.052	0.069
		75	0.063	0.078	0.069	0.066	0.078
		100	0.047	0.062	0.052	0.052	0.059
		500	0.044	0.046	0.044	0.044	0.044

*BC, bias-corrected bootstrap confidence interval; stBC, significance-tested bias-corrected bootstrap confidence interval; rBC, reduced bias-corrected bootstrap confidence interval; WBC, 30% Winsorized bias-corrected bootstrap confidence interval; P, percentile bootstrap confidence interval*.

When *a* = *b* = 0, all methods were too conservative regardless of sample size, and the WBCBCI and PBCI attained the lowest observed type I error rate of 0 when *n* = 25. All other instances of the methods' type I error rates falling below the lower limit of Bradley's liberal robustness criterion occurred when either the *a*-path or the *b*-path was equal to 0.14 and the sample size was less than or equal to 100. The WBCBCI and the PBCI had type I error rates that were too conservative in the highest number of conditions, with their type I error rates falling below the lower limit of Bradley's liberal robustness criterion in 13 of the 35 conditions where *ab* = 0. On the other hand, the BCBCI and the stBCBCI had type I error rates that were too liberal in the highest number of conditions, with their type I error rates falling above the upper limit of Bradley's liberal robustness criterion in 8 of the 35 conditions. The only other method with a type I error rate that exceeded the upper limit of the criterion was the rBCBCI, which had a type I error rate of 0.077 when *b* = 0.59 and *n* = 100. The maximum observed type I error rate of 0.088 was exhibited by both the BCBCI and the stBCBCI when *b* = 0.39 and the sample size was 100 and again by the BCBCI when *a* = 0.59 and *n* = 25.

In summary, the type I error rates of all methods were too conservative when *a* = *b* = 0 in every sample size condition. When the size of the *a*-path or *b*-path was 0.14, type I error rates increased as sample size increased overall. The relationship between type I error rate and sample size was noticeably nonmonotonic when the nonzero path was 0.39 or 0.59, with type I error rates increasing until about n = 75 or *n* = 100 before they decreased to near the target type I error rate of 0.05 by *n* = 500. Regardless of the condition, however, the order of the methods almost always remained the same, with the order from highest type I error rate to lowest being: BCBCI, stBCBCI, rBCBCI, WBCBCI, and PBCI.

### 5.3. Power

**Figure 6:**
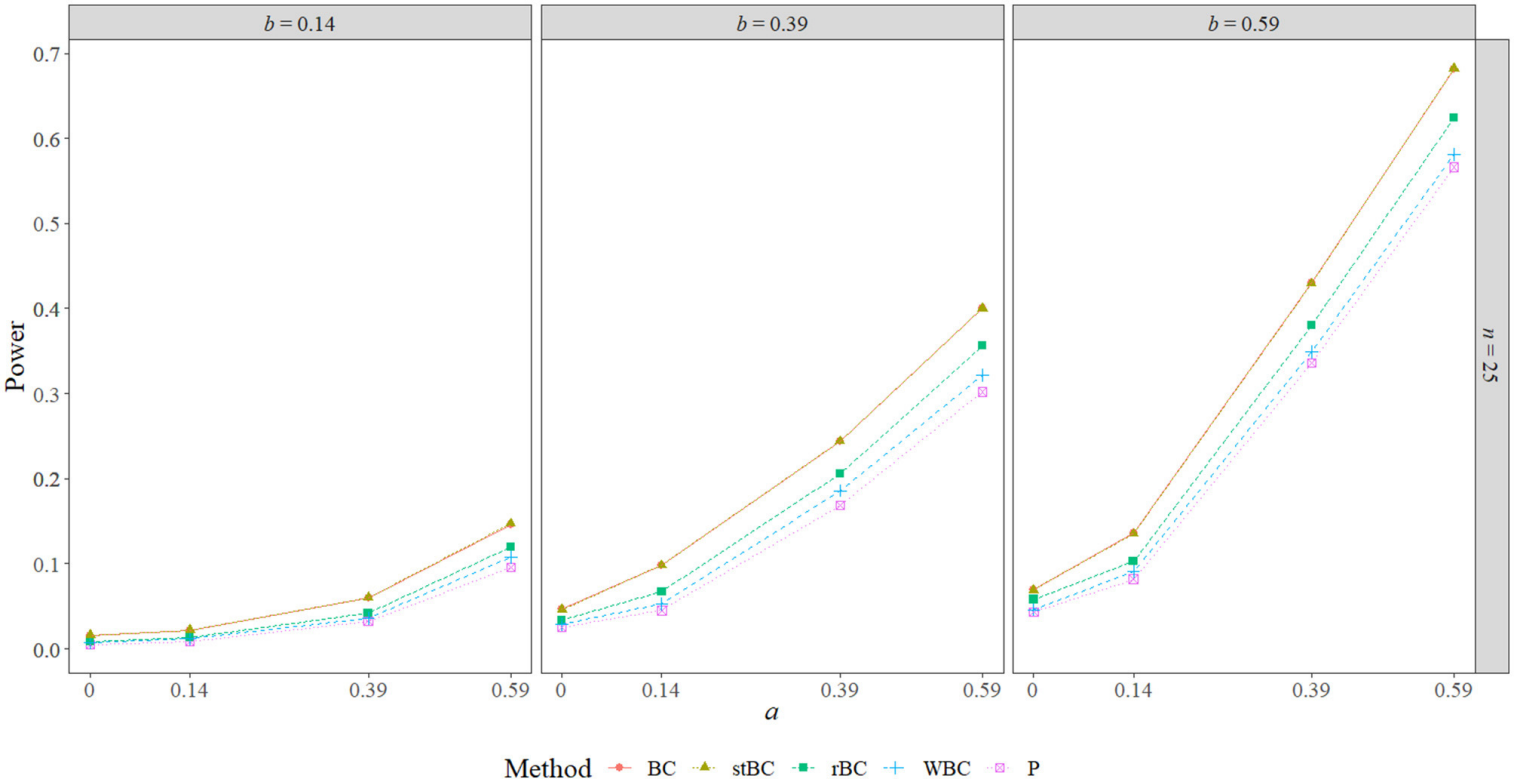
Power of all methods when *n* = 25 across the range of *b*-path sizes and *a*-path sizes. BC, bias-corrected bootstrap confidence interval; stBC, significance-tested bias-corrected bootstrap confidence interval; rBC, reduced bias-corrected bootstrap confidence interval; WBC, 30% Winsorized bias-corrected bootstrap confidence interval; P, percentile bootstrap confidence interval.

**Figure 7:**
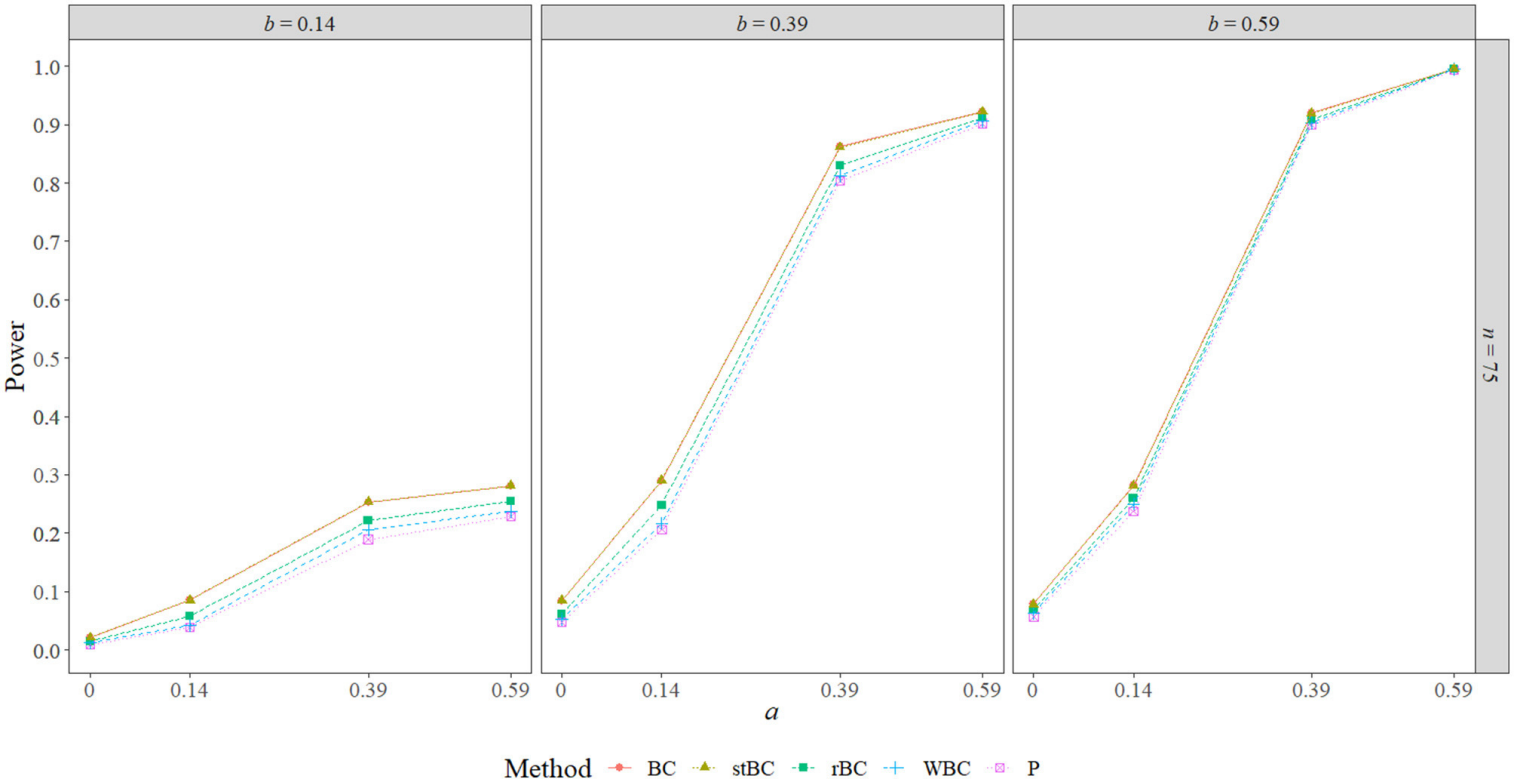
Power of all methods when *n* = 75 across the range of *b*-path sizes and *a*-path sizes. BC, bias-corrected bootstrap confidence interval; stBC, significance-tested bias-corrected bootstrap confidence interval; rBC, reduced bias-corrected bootstrap confidence interval; WBC, 30% Winsorized bias-corrected bootstrap confidence interval; P, percentile bootstrap confidence interval.

**Figure 8:**
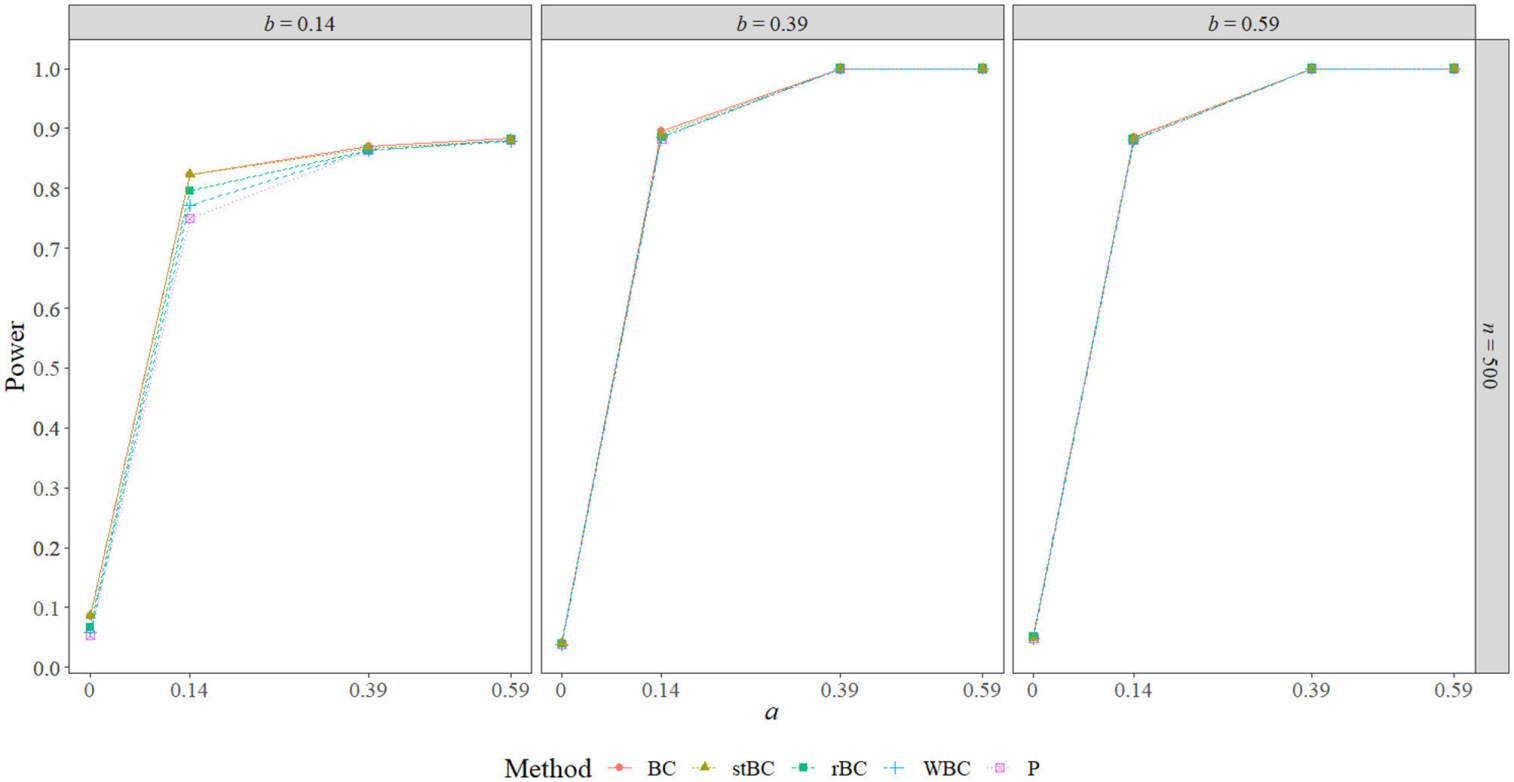
Power of all methods when *n* = 500 across the range of *b*-path sizes and *a*-path sizes. BC, bias-corrected bootstrap confidence interval; stBC, significance-tested bias-corrected bootstrap confidence interval; rBC, reduced bias-corrected bootstrap confidence interval; WBC, 30% Winsorized bias-corrected bootstrap confidence interval; P, percentile bootstrap confidence interval.

Figures 6–8 display the power of all the methods when the sample size was 25, 75, and 500, respectively. Additional power figures are available in the Supplementary Material, and the power values for all conditions are also available in Table 2. As expected, empirical power increased as both sample size and the sizes of the *a*- and *b*-paths increased, reaching one when *a* and/or *b* was 0.39 or 0.59 and the sample size was 100 or 500. With rare exceptions (where the difference in power was at most 0.03), the order of the methods remained the same across all conditions, with the BCBCI having the highest power followed by the stBCBCI, rBCBCI, WBCBCI, and finally the PBCI with the lowest power. All main effects of the method, sample size, *a*-path size, and *b*-path size factors and all two way interactions were significant (likelihood ratio test *p* < 0.001). Also, the three-way interaction between *a*-path size, *b*-path size, and sample size was significant as well (likelihood ratio test *p* < 0.001). See the Supplementary Material for more information.

**Table 2:** Power of all methods across all conditions where *ab*≠0.

		**Bootstrap Method**				

**a**	**b**	**Sample Size**	***P***	**BC**	**rBC**	**WBC**	**stBC**
0.14	0.14	25	0.009	0.022	0.013	0.012	0.022
		50	0.020	0.049	0.031	0.024	0.049
		75	0.039	0.085	0.058	0.042	0.085
		100	0.069	0.125	0.094	0.079	0.125
		500	0.749	0.823	0.795	0.772	0.823
0.14	0.39	25	0.045	0.098	0.067	0.053	0.098
		50	0.123	0.198	0.161	0.133	0.198
		75	0.206	0.290	0.248	0.217	0.290
		100	0.267	0.344	0.307	0.285	0.343
		500	0.883	0.896	0.886	0.885	0.890
0.14	0.59	25	0.082	0.136	0.103	0.091	0.135
		50	0.158	0.209	0.182	0.169	0.208
		75	0.237	0.282	0.260	0.250	0.281
		100	0.296	0.329	0.311	0.304	0.327
		500	0.880	0.885	0.881	0.881	0.883
0.39	0.14	25	0.032	0.060	0.042	0.036	0.060
		50	0.128	0.187	0.158	0.139	0.187
		75	0.189	0.253	0.221	0.205	0.253
		100	0.292	0.353	0.319	0.303	0.352
		500	0.864	0.870	0.864	0.863	0.867
0.39	0.39	25	0.169	0.244	0.206	0.186	0.244
		50	0.501	0.607	0.551	0.523	0.606
		75	0.804	0.863	0.830	0.812	0.861
		100	0.920	0.953	0.938	0.928	0.953
		500	1.000	1.000	1.000	1.000	1.000
0.39	0.59	25	0.336	0.430	0.380	0.349	0.429
		50	0.732	0.791	0.765	0.744	0.790
		75	0.899	0.921	0.908	0.903	0.919
		100	0.964	0.971	0.968	0.966	0.971
		500	1.000	1.000	1.000	1.000	1.000
0.59	0.14	25	0.096	0.146	0.120	0.108	0.147
		50	0.169	0.220	0.195	0.179	0.219
		75	0.229	0.281	0.255	0.237	0.281
		100	0.305	0.352	0.332	0.317	0.350
		500	0.881	0.884	0.881	0.878	0.880
0.59	0.39	25	0.302	0.400	0.356	0.322	0.400
		50	0.734	0.795	0.766	0.746	0.792
		75	0.902	0.922	0.911	0.906	0.922
		100	0.970	0.978	0.976	0.971	0.975
		500	1.000	1.000	1.000	1.000	1.000
0.59	0.59	25	0.566	0.681	0.624	0.581	0.682
		50	0.939	0.960	0.952	0.942	0.960
		75	0.993	0.996	0.995	0.995	0.995
		100	0.999	1.000	0.999	0.999	1.000
		500	1.000	1.000	1.000	1.000	1.000

*BC, bias-corrected bootstrap confidence interval; stBC, significance-tested bias-corrected bootstrap confidence interval; rBC, reduced bias-corrected bootstrap confidence interval; WBC, 30% Winsorized bias-corrected bootstrap confidence interval; P, percentile bootstrap confidence interval*.

### 5.4. Balance

**Figure 9:**
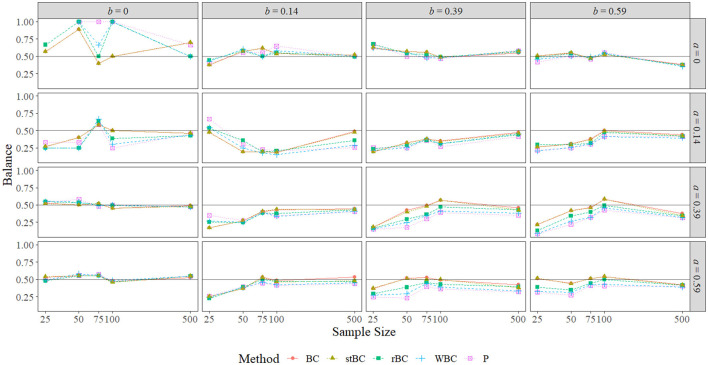
Balance of all methods across the range of *a*-path sizes, *b*-path sizes, and sample sizes. BC, bias-corrected bootstrap confidence interval; stBC, significance-tested bias-corrected bootstrap confidence interval; rBC, reduced bias-corrected bootstrap confidence interval; WBC, 30% Winsorized bias-corrected bootstrap confidence interval; P, percentile bootstrap confidence interval. The black horizontal line at .50 on the graphs represents perfect balance. The *x*-axis is on the natural log scale.

Figure 9 displays the balance of the 95% bootstrap confidence interval methods used. Recall that a value of 0.50 indicates that exactly half of the true indirect effects not captured by the confidence interval were below the lower limit, and so the confidence interval was perfectly balanced. Thus, the black horizontal line at 0.50 on the graphs indicates perfect balance. A point above the line indicates a condition in which the confidence interval fell above the true effect more often than it fell below, and a point below the line indicates a condition in which the confidence interval fell below the true effect more often than it fell above. Note that there is no data point for either the PBCI or the WBCBCI in the *a* = *b* = 0 graph at the sample size of 25 because these methods captured every true indirect effect in this condition.

With the exception of the conditions in which *a* = *b* = 0 and *a* = *b* = 0.14, the balance of all methods seemed to converge to the same value as sample size grew to 500. The worst balance occurred when *a* = *b* = 0, particularly when the sample size was 50 or 100 for the rBCBCI and the WBCBCI or when the sample size was 50, 75, or 100 for the PBCI. At these sample sizes, every time these confidence intervals failed to capture the true indirect effect, they fell above the true effect, but this was largely due to the low number of true indirect effects excluded at these sample sizes. For example, the rBCBCI, WBCBCI, and PBCI only excluded zero a single time when the sample size was set to 100. In fact, the WBCBCI and PBCI never excluded zero more than four times for a given sample size when *a* = *b* = 0. Thus, large disparities in balance were the result of very small differences in the number of true indirect effects excluded when both the *a*-path and *b*-path were set to zero.

**Figure 10:**
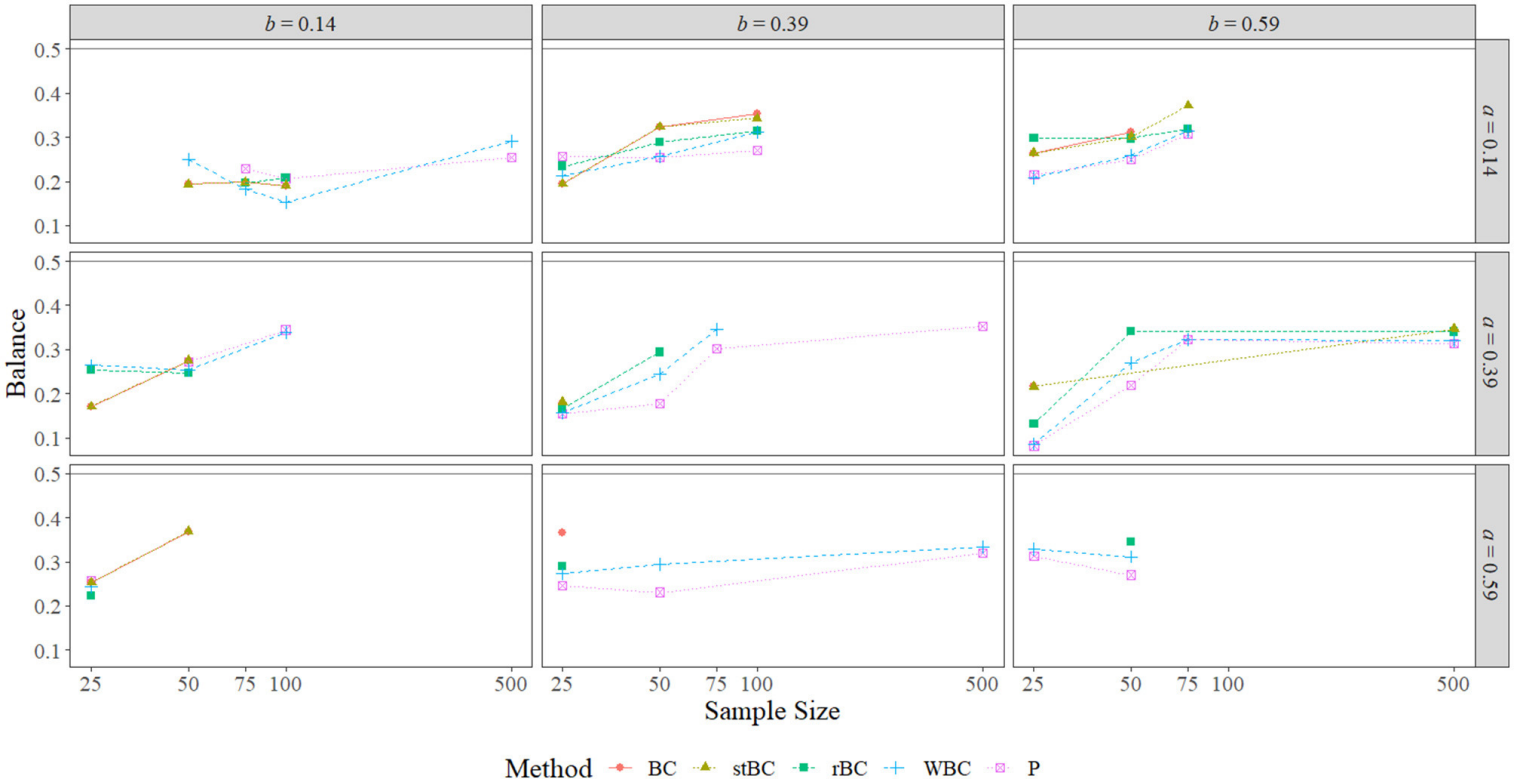
Balance of all methods that are significant at α = 0.05 across the range of all nonzero *a*-path sizes, nonzero *b*-path sizes, and sample sizes. BC, bias-corrected bootstrap confidence interval; stBC, significance-tested bias-corrected bootstrap confidence interval; rBC, reduced bias-corrected bootstrap confidence interval; WBC, 30% Winsorized bias-corrected bootstrap confidence interval; P, percentile bootstrap confidence interval. The black horizontal line at .50 on the graphs represents the null hypothesis of perfect balance. The *x*-axis is on the natural log scale.

The BCBCI and stBCBCI were more balanced than the other three methods when *a* = *b* = 0 and the sample size was 50 or 100, and when the sample size was 100 the BCBCI and the stBCBCI were perfectly balanced (falling above zero three times each and falling below zero three times each). In fact, the BCBCI and the stBCBCI were the best balanced methods overall, achieving the proportions closest to 0.50 in a combined total of 49 of the 80 conditions (one time both tying with the PBCI). The rBCBCI and WBCBCI, which tied in two conditions, had the best balance in 14 and 8 conditions, respectively. Finally, the PBCI had the best balance in 12 conditions, tying once with both the BCBCI and stBCBCI and one other time with the WBCBCI. Besides the conditions in which *a* = *b* = 0, the patterns of balance were similar across methods, with the BCBCI and stBCBCI most often closest to perfectly balanced followed by the rBCBCI, PBCI, and WBCBCI. Examining only the proportions that were significantly different from .50 in Figure 10 reveals the same pattern of balance, with the BCBCI significantly different in only 16 conditions, the stBCBCI in 17, the rBCBCI in 18, the PBCI in 25, and the WBCBCI in 26. Please note that only the nonzero *ab* conditions are displayed in the figure because only one condition resulted in a significant proportion when *ab* = 0: Both the BCBCI and stBCBCI had a significant balance value of 0.899 when *a* = *b* = 0 and *n* = 50 (the balance values of the other three methods were actually larger, but not significant due to a fewer number of true indirect effects being excluded by their confidence intervals).

In summary, the balance of all methods converged toward the same value as sample size increased in almost all conditions. The worst balance was witnessed in the *a* = *b* = 0 conditions, but this was largely due to only a very few true effects being excluded by the methods in these conditions. The overall order of the methods from best balanced to worst balanced was: BCBCI, stBCBCI, rBCBCI, PBCI, and WBCBCI. One feature of the 90% and 80% conditions worth mentioning here is that, while the number of times the balance for the PBCI, WBCBCI, and rBCBCI was significantly different from 0.50 increased as the confidence level decreased, this number for the BCBCI and stBCBCI actually *decreased* slightly as the confidence level decreased (see the Supplementary Material).

## 6. Simulation Summary

The simulation reveals that the bootstrap methods fall on a continuum. From lowest type I error rate and power to highest type I error rate and power, the order went PBCI, WBCBCI, rBCBCI, stBCBCI, and BCBCI. The order from worst balance to best balance was the same with the exception that the positions of the WBCBCI and PBCI were switched.

In terms of bias, the simulation study empirically demonstrated that the BCBCI's bias correction is not implemented based on sample mean bias or bootstrap mean bias (i.e., the difference between the mean sample indirect effect and the true indirect effect or the difference between the mean bootstrap indirect effect and the sample indirect effect). While bootstrap mean bias was near zero in all conditions and sample mean bias near-monotonically decreased to zero as sample size increased, the simulation's type I error rate results clearly show that the bias correction does not have the same relationship with sample size. In Figures 4 and 5, differences between the type I error rates of the PBCI and the BCBCI illustrate how large the BCBCI's bias correction was: The bigger the difference in their type I error rates, the more the PBCI's limits were shifted by the bias correction to produce the BCBCI. The difference between these rates never decreases to zero monotonically with sample size, indicating that the bias correction is not shrinking just because sample size is increasing. In fact, some of the largest differences in type I error rates occurred at the largest sample size of 500 when *a* or *b* was 0.14, and thus the bias correction was near its largest at this sample size as well. Clearly, it is not mean bias that is influencing the BCBCI's bias correction. Instead, it is median bias.

One notable feature of the simulation was that the type I error rates of all methods often surpassed the α = 0.05 level that corresponds to a 95% confidence interval. Since power is a function of α, increasing as the α-level increases, power may be artificially inflated by these liberal type I error rates, and thus comparing the power of these methods without consideration for their type I error rates may be misleading. To address this issue, a supplementary simulation was run that compared the power of each bias-corrected method to a corresponding PBCI set to the maximum type I error rate achieved by the bias-corrected method.

## 7. Supplementary Power Simulation

After the primary simulation was complete, the maximum type I error rate found for each of the four 95% bias-corrected confidence intervals was recorded. In this supplementary simulation, the maximum type I error rate achieved by each bias-corrected method was used to determine the α-level at which to set a (1−α) × 100% PBCI for comparison. For example, the maximum type I error rate found for the rBCBCI in the primary simulation was 0.077, and so a (1−0.077) × 100 = 92.3% PBCI was calculated to compare statistical power with the rBCBCI in this simulation. Setting the PBCI (the method with the lowest observed power in the primary simulation) to a confidence level corresponding to the maximum type I error rate achieved by each bias-corrected method should offer a worst-case-scenario comparison, ensuring that any power differences due to type I error rate inflation are removed even if each method is at its most liberal. Using the maximum type I error rate of each method should be a more informative option than using the average type I error rate of each method; the overly conservative type I error rates the methods achieved in the primary simulation's *a* = *b* = 0 conditions brought their mean error rates down to almost nominal levels, resulting in comparison PBCIs that would be set near a 95% confidence level like the PBCI in Figures 6–8.

### 7.1. Manipulated Factors

The manipulated factors in this simulation were exactly the same as those in the primary simulation, but the conditions in which *ab* = 0 are not focused on since the outcome of interest in this simulation was power.

### 7.2. Data Generation

The exact same procedure was followed to generate data in this simulation as was used in the primary simulation, except four different PBCIs were formed to be compared with the four 95% bias-corrected bootstrap confidence interval methods. The confidence level of each PBCI was determined by the maximum type I error rate its corresponding bias-corrected method(s) reached during the first simulation. These type I error rates were 0.088 for the BCBCI and stBCBCI (resulting in a 91.2% comparison PBCI), 0.077 for the rBCBCI (resulting in a 92.3% comparison PBCI), and 0.073 for the WBCBCI (resulting in a 92.7% comparison PBCI).

### 7.3. Measured Outcome

The power of the bootstrap methods was calculated the same way it was in the primary simulation.

## 8. Supplementary Results

**Figure 11:**
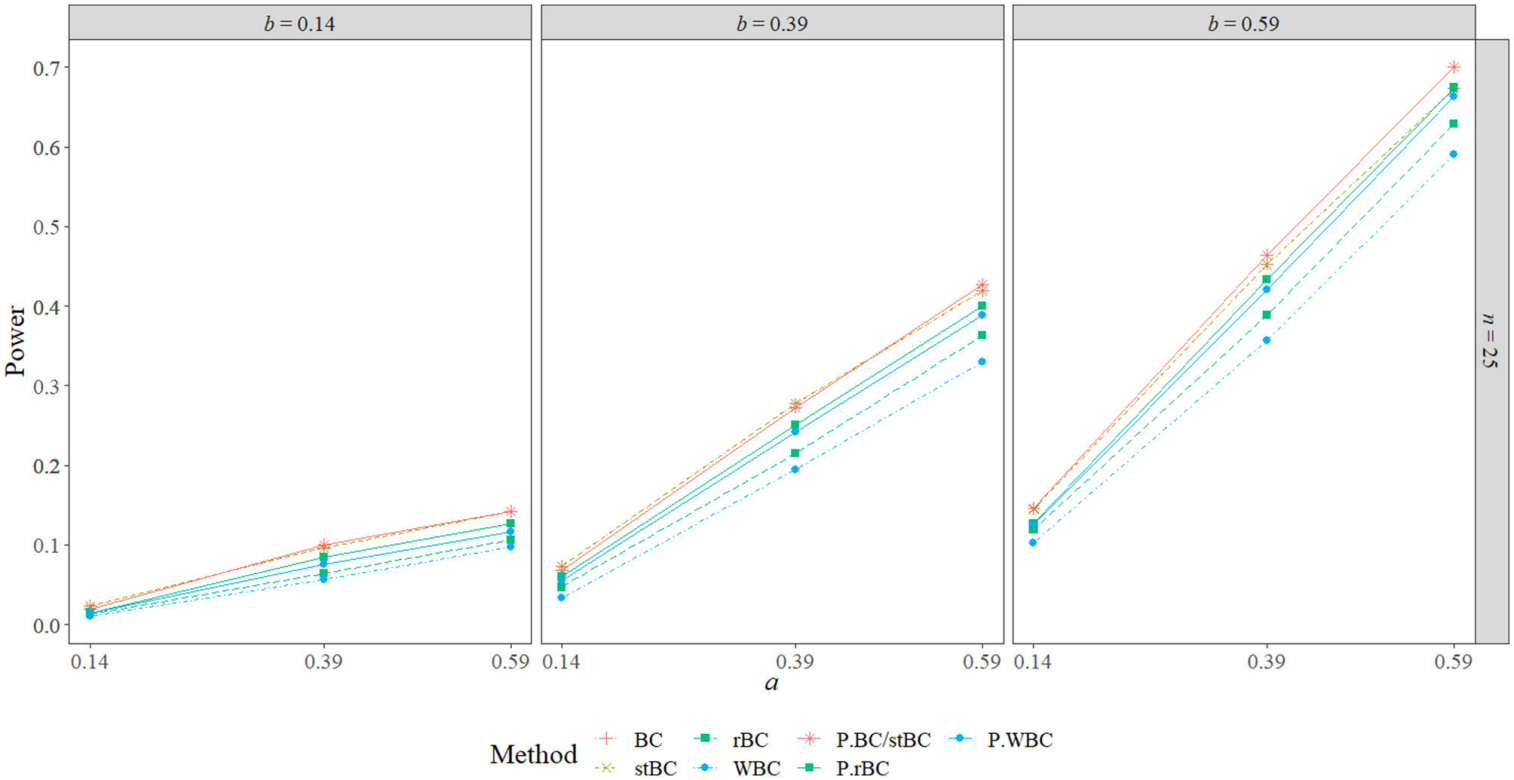
Power of all methods compared to PBCI controlling for type I error rate when *n* = 25 across the range of *b*-path sizes and *a*-path sizes. BC, bias-corrected bootstrap confidence interval; stBC, significance-tested bias-corrected bootstrap confidence interval; rBC, reduced bias-corrected bootstrap confidence interval; WBC, 30% Winsorized bias-corrected bootstrap confidence interval; P.BC/stBC, comparison percentile bootstrap confidence interval for BC and stBC; P.rBC, comparison percentile bootstrap confidence interval for rBC; P.WBC, comparison percentile bootstrap confidence interval for WBC.

**Figure 12:**
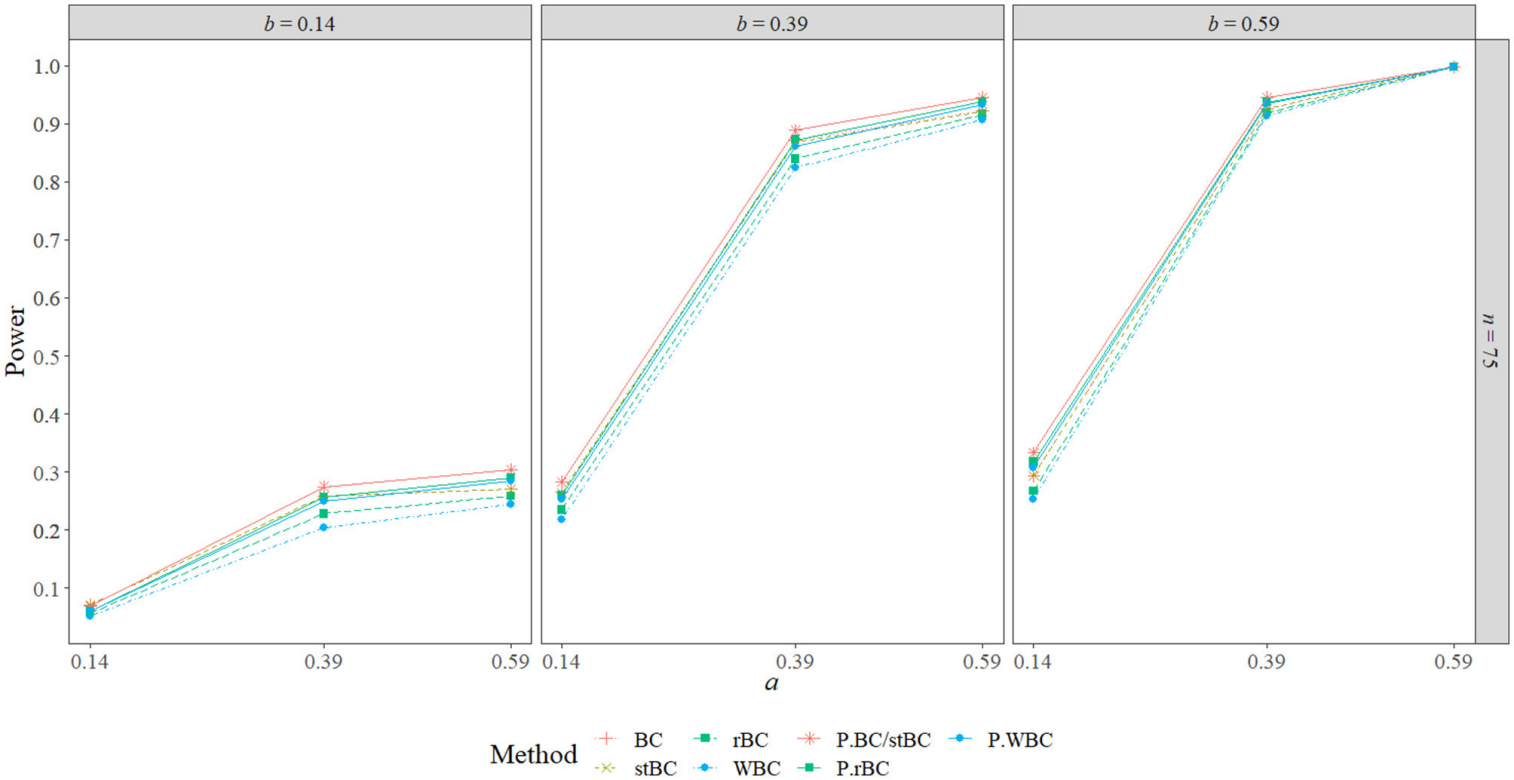
Power of all methods compared to PBCI controlling for type I error rate when *n* = 75 across the range of *b*-path sizes, *a*-path sizes. BC, bias-corrected bootstrap confidence interval; stBC, significance-tested bias-corrected bootstrap confidence interval; rBC, reduced bias-corrected bootstrap confidence interval; WBC, 30% Winsorized bias-corrected bootstrap confidence interval; P.BC/stBC, comparison percentile bootstrap confidence interval for BC and stBC; P.rBC, comparison percentile bootstrap confidence interval for rBC; P.WBC, comparison percentile bootstrap confidence interval for WBC.

**Figure 13:**
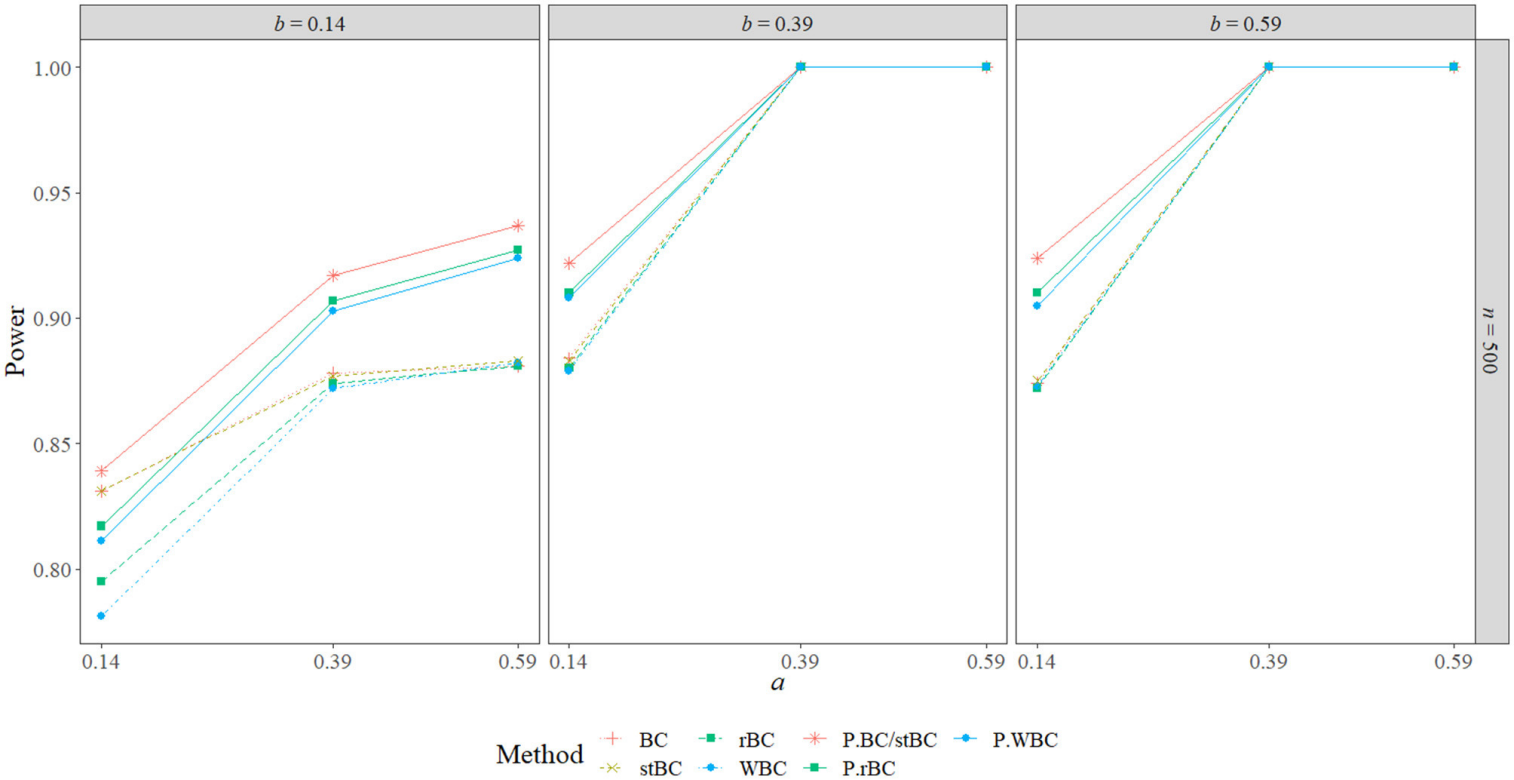
Power of all methods compared to PBCI controlling for type I error rate when *n* = 500 across the range of *b*-path sizes and *a*-path sizes. BC, bias-corrected bootstrap confidence interval; stBC, significance-tested bias-corrected bootstrap confidence interval; rBC, reduced bias-corrected bootstrap confidence interval; WBC, 30% Winsorized bias-corrected bootstrap confidence interval; P.BC/stBC, comparison percentile bootstrap confidence interval for BC and stBC; P.rBC, comparison percentile bootstrap confidence interval for rBC; P.WBC, comparison percentile bootstrap confidence interval for WBC.

Figures 11–13 present the BCBCI, stBCBCI, rBCBCI, and WBCBCI plotted with their comparison PBCIs that control for the inflated type I error rates of each method at the sample sizes of 25, 75, and 500, respectively (additional figures are available in the Supplementary Material). As can be seen in the figures, the advantages in terms of power of the bias-corrected methods over the PBCI that were evident in Figures 6–8 are no longer present, with the adjusted PBCIs achieving higher power than their corresponding bias-corrected methods in all but six conditions (in which both the BCBCI and stBCBCI obtained higher power, but the difference in power was never more than 0.005). Thus, controlling for type I error rate, the bias-corrected methods did not seem to perform any better in terms of power than the PBCI. There still appears to be an advantage in terms of balance, however: The BCBCI's and stBCBCI's balance values were significantly different from 0.50 in 16 and 17 conditions, respectively, while the balance of their corresponding control PBCI was significantly different in 34. The balance values of the rBCBCI and WBCBCI were significantly different from 0.50 in 18 and 26 conditions, respectively, while the balance values of their corresponding control PBCIs were significantly different in 31 conditions each (see the corresponding figure in the Supplementary Material).

## 9. General Discussion

The present study compared the PBCI, BCBCI, and WBCBCI to two new alternative bias-corrected bootstrap techniques for the indirect effect: the stBCBCI and the rBCBCI. Performance measures included type I error rate, power, and balance. In terms of balance, the results of this study agree with those of Chen and Fritz (2021), who found that the BCBCI offered overall better balance than other bootstrap methods. The tendency of the methods' balance values to grow more and more similar as sample size increased in our simulation replicates their findings as well. Also in line with previous research, the BCBCI exhibited the most elevated type I error rates and the highest power, and the PBCI had the most control over type I error rates and the lowest overall power. Similarly, the finding that all methods were too conservative when *a* = *b* = 0 reflects the results of previous simulation studies comparing inferential methods for the indirect effect (Biesanz et al., 2010; Fritz et al., 2012; Chen and Fritz, 2021). As explained by Fritz et al. (2012), this is because the sizes of both the *a*-path and the *b*-path matter in determining the significance of a sample estimate of a true indirect effect. When both *a* and *b* are zero, $â\hat{b}$ will be close to zero as well, while if *a* is zero and *b* is 0.59, for example, the product of their sample estimates will likely be farther from zero. This means that a sample estimate of a true indirect effect with *a* = *b* = 0 is the most likely to be close to zero and the least likely to significantly differ from zero, resulting in the smallest number of type I errors. One method for inference in mediation which does not suffer from this conservative type I error rate issue is the model-based constrained optimization procedure, as proposed by Tofighi and Kelley (2020). Through this procedure, two models are fit: one where the indirect effect is constrained to zero (i.e., *ab* = 0) and one where it is freely estimated. The resulting models are then compared using a likelihood ratio test. This method seems to have more accurate type I error rates due to fitting the constrained model, thus finding the most likely model which fits the null hypothesis among many candidates.

Across all conditions, the stBCBCI performed very similarly to the BCBCI on all performance criteria, and the WBCBCI performed quite similarly to the PBCI. Increasing the percentage of trimming should make the WBCBCI more and more similar to the PBCI, with the 50% Winsorized BCBCI producing the exact same confidence intervals as the PBCI (Chen and Fritz, 2021). The rBCBCI, on the other hand, offered a balance of the benefits and shortcomings of the BCBCI and the PBCI, falling somewhere in the middle on all performance criteria.

Although the goal of this research was to develop a method that maintained the high power and balance of the BCBCI while still controlling the type I error rate, the primary simulation revealed that there was always a tradeoff between the three performance measures. Across conditions, the methods examined fell on a continuum, which in order of increasing type I error rate, power, and balance went: PBCI, WBCBCI, rBCBCI, stBCBCI, and BCBCI (with the exception that the WBCBCI had slightly worse balance than the PBCI). Thus, a method with overall better balance and higher power also had higher type I error rates than its competitors. Decreasing the confidence level of the PBCI in the supplementary simulation seemed to provide the same power benefit using any of the bias-corrected methods did, however, and thus the only clear advantage of the bias-corrected methods is the better balance they provide. As such, if controlling for false positives is more important than finding an effect if it exists, the PBCI is still recommended. This might be the case, for example, in an experiment testing the efficacy of a drug that can have severe side effects and is designed to treat a non-life threatening condition: It is important that the drug only be found effective if we are quite confident it can help significantly; otherwise, we risk recommending an ineffective drug that has a high potential to harm its users. If detecting a true effect is most important but control over the type I error rate is still a concern, the rBCBCI is a good compromise between the PBCI and the BCBCI that has the benefit of being better balanced than the former method. A situation where the rBCBCI might thus be useful would be an experiment to see if an expensive drug designed to treat a debilitating condition is effective: It is important to find an effect of the drug if it exists so that people suffering from the condition can get help. At the same time, however, protection against finding an effect when one does not exist is warranted so we decrease the risk of selling a useless drug that will cost patients a great deal of money. Balance is also beneficial here so there is no worry our confidence interval tends to miss the true effect in one direction more than the other. These decisions about which method to use are only impactful when the sample size is small, however; in almost all effect size conditions in the primary simulation, differences between methods on all three performance measures decreased with sample size to the point that, with *n* = 500, the performance of each method had converged to nearly the same value.

Regardless of condition, the performance of the stBCBCI closely resembled the performance of the BCBCI in terms of type I error rate, power, and balance. This was due to how often the stBCBCI's significance test indicated a significant difference between the median of the observed bootstrap sampling distribution and the corresponding sample indirect effect estimate. On average, this significance test rejected the null hypothesis in over 72% of the iterations in each condition: about 71% of the time when the true indirect effect was zero and about 74% of the time when the true indirect effect was nonzero. Thus, the stBCBCI used the bias-corrected bootstrap for its confidence interval most of the time. Since the BCBCI was used almost three times more often than the PBCI, it makes sense that the stBCBCI performed very similarly to the former test. In fact, with 5,000 bootstrap replications representing the sample size of each trial, the binomial test of the stBCBCI would reach significance at an α-level of 0.05 if the observed proportion was greater than 0.0138 away from 0.50, and so even minor deviations away from the median of the observed bootstrap distribution resulted in the BCBCI being employed. Using a more conservative α-level (e.g., α = 0.01) may be worthwhile to increase the significance threshold. Furthermore, because the stBCBCI employs two tests of significance in order to conduct inference on the indirect effect (i.e., the binomial test of median bias to determine which bootstrap confidence interval to use and then the bootstrap confidence interval itself to determine the significance of the indirect effect), a more conservative α-level would help alleviate any multiple testing issues that could arguably arise.

Still, the frequent significance of the stBCBCI's test of median bias highlights the fact that median and mean bias are distinct quantities. While the discrepancy between the sample indirect effect and the median of the observed bootstrap sampling distribution was often large enough to warrant the use of the stBCBCI, bootstrap mean bias (i.e., mean bias of the bootstrap indirect effect) recorded during the simulation remained near zero across all conditions. In 76 of the 80 conditions observed in this study, bootstrap mean bias was smaller than sample mean bias (i.e., mean bias of the sample indirect effect). Further exploration of the differences between the mean and median bias properties of the indirect effect, and how well they agree with the bias properties assumed under the bias correction of the BCBCI, could advance our understanding of the BCBCI's appropriateness when applied to the indirect effect. Diagnostic functions like those discussed in Efron (1982b) could also help assess whether the existence of the function *g*(·) is a reasonable assumption for the indirect effect.

### 9.1. Limitations and Future Directions

The following section discusses several limitations of the current study, including the conclusions made regarding balance, the lack of missing data, the lack of confounding variables, and the use of a simple mediation model within the ordinary least squares (OLS) regression framework. Potential future directions are also described to address these issues and other questions that remain regarding the BCBCI.

The worst balance among the methods examined in this study was observed in conditions in which *a* = *b* = 0. However, these were also the conditions in which the fewest number of true indirect effects were excluded by the confidence intervals. For example, in the *a* = *b* = 0 and *n* = 100 condition, the rBCBCI, PBCI, and WBCBCI never had any confidence intervals fall below the true indirect effect because only one true effect was ever excluded by these bootstrap methods across all 1,000 iterations of the condition. Thus, the confidence intervals never fell below the true effect, but they also only fell above it once. Due to very small numbers of indirect effects being excluded by confidence intervals in this condition and others in which *a* = *b* = 0, more simulations should be run to verify the accuracy of the balance values found in this study.

Future simulation studies should also examine the impact of missing data on these methods. In real-world scenarios, it is quite common for some data values to be missing, and thus methods of handling missing data must be applied in conjunction with bootstrapping to draw accurate inferences about the data. Future research should combine the bias-corrected bootstrap methods discussed here with popular missing data methods in the presence of different missing data mechanisms to see how their performances change and whether they offer any performance advantages over the PBCI in such situations. For existing work on missing data handling procedures combined with bootstrapping for the indirect effect, see Wu and Jia (2013) and Zhang and Wang (2013).

Another real-world complication ignored in this study is the presence of confounding variables. Every mediation model used in the simulations was correctly specified, meaning that the form of the model fit to the data matched the underlying relationships between the variables in the population. These are conditions in which any bias present should be at a minimum. With an unknown third variable present, the indirect effect produced by the mediation model may be more biased (see Valente et al., 2017 for an overview of confounding and how to address it in mediation analyses), and thus the bias-corrected methods may perform differently. It would be informative to see how the BCBCI and the other bias-correction variants perform in relation to the PBCI when the indirect effect estimate is (median) biased by a confounding variable in future work. Other conditions that lead to violations of model assumptions, such as nonconstant error variance and the presence of outliers, should also be explored in future research to increase the generalizability of these results to real, less ideal datasets.

In addition to being correctly specified and being applied to data meeting all necessary model assumptions, the models included in these simulation studies were all simple mediation models estimated using OLS regression rather than the also-popular structural equation modeling (SEM) framework. Still, OLS regression mediation models are mathematically equivalent to identified SEM mediation models when the mediator and outcome variables are continuous and observed, so these results are expected to generalize to corresponding SEM simple mediation models (e.g., see Rijnhart et al., 2017). For a general introduction to SEM in mediation analysis, see Gunzler et al. (2013).

The inflated type I error rates of the BCBCI applied to the indirect effect call into question its ability to perform in areas of statistics outside of mediation analysis as well. For example, Karlsson (2009) found evidence that the BCBCI applied to a weighted nonlinear quantile regression estimator for longitudinal data resulted in significant undercoverage—i.e., the true value being captured by the interval less often than it should according to the set confidence level—and recommended using the PBCI instead. Identifying and examining other BCBCI problem areas such as this in future studies can help further understanding of the BCBCI's issues and lead to the development of improved bias-corrections for the sample indirect effect and other statistics. Applying the new bias-corrected methods discussed in this study to other statistics would be worthwhile as well to see how they perform in these other areas. On the other hand, adapting novel bootstrapping procedures applied in other areas of research (e.g., the iterated bootstrap confidence interval approach discussed in Lee and Young (1995) or Davidson and MacKinnon (2010)'s wild bootstrap procedure) for use with the indirect effect in future studies could also prove fruitful.

Although—in correctly specified, complete data mediation analyses at least—the benefits of the bias correction are still accompanied by inflated type I error rates after the completion of this study, it is possible that there is still an alteration to the BCBCI's bias correction that can wed its increased power with the PBCI's control over the type I error rate. Thus, future research should continue examining ways in which the BCBCI can be tailored to the indirect effect in small samples to make it the robust alternative to the PBCI it was once believed to be.

## Data Availability Statement

The datasets presented in this study can be found in an online repository. The name of the repository can be found below: https://osf.io/mncjp/.

## Author Contributions

TT contributed to investigation, software, formal analysis, visualization, writing the original draft, and reviewing and editing the manuscript. AM contributed to conceptualization, investigation, supervision, and reviewing and editing the manuscript. Both authors contributed to the article and approved the submitted version.

## Funding

This material is based upon work supported by the National Science Foundation Graduate Research Fellowship Program under grant no. DGE-2034835.

## Author Disclaimer

Any opinions, findings, and conclusions or recommendations expressed in this material are those of the author(s) and do not necessarily reflect the views of the National Science Foundation.

## Conflict of Interest

The authors declare that the research was conducted in the absence of any commercial or financial relationships that could be construed as a potential conflict of interest.

## Publisher's Note

All claims expressed in this article are solely those of the authors and do not necessarily represent those of their affiliated organizations, or those of the publisher, the editors and the reviewers. Any product that may be evaluated in this article, or claim that may be made by its manufacturer, is not guaranteed or endorsed by the publisher.
