# Supplementary material for: Correcting the Bias Correction for the Bootstrap Confidence Interval in Mediation Analysis
Source: Front Psychol. 2022 May 27;13:810258. doi: 10.3389/fpsyg.2022.810258 (PMC9197131; doi:10.3389/fpsyg.2022.810258)
Supplement: Supplementary file 1 [file Data_Sheet_1.pdf]

Correcting the Bias Correction for the Bootstrap Confidence Interval in Mediation Analysis

Supplementary Material

Tristan D. Tibbe, Amanda K. Montoya

Type I Error Rate Graphs

Confidence Level = 90%

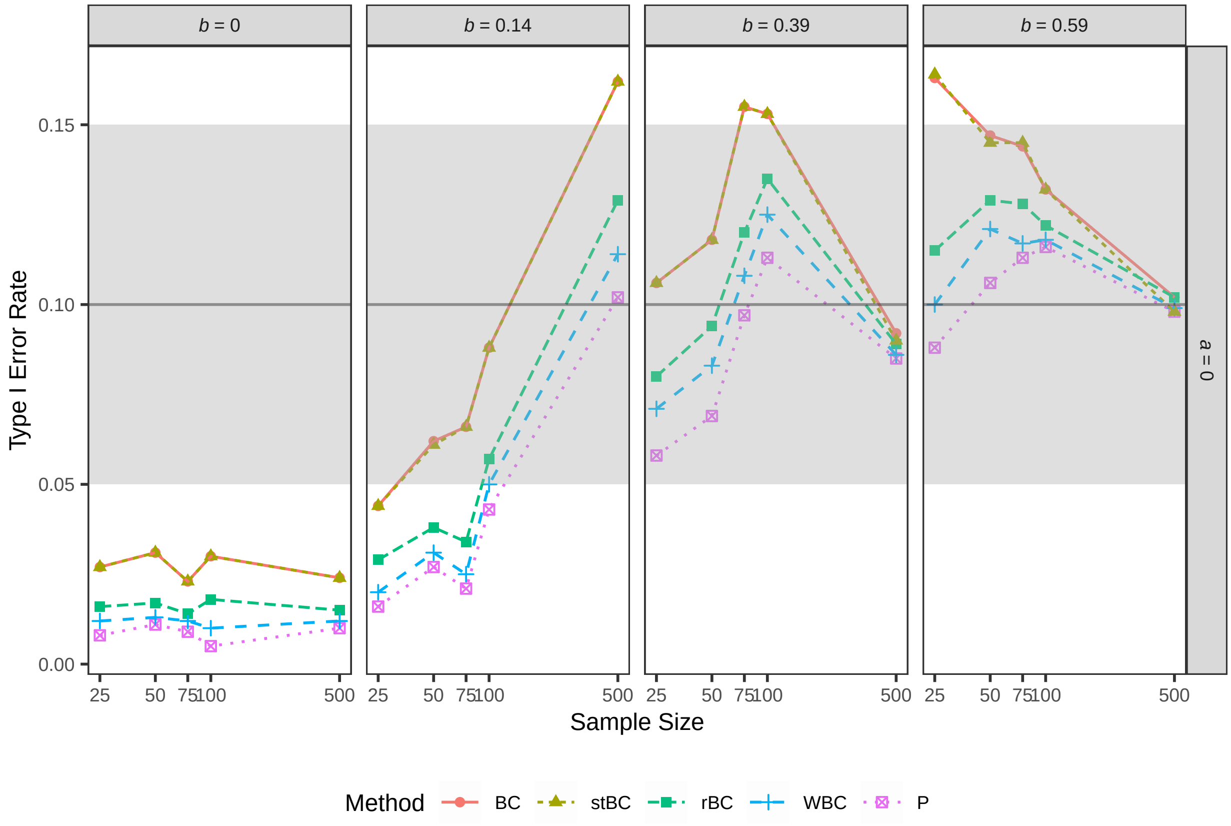

SM Figure 1. Type I error rate of all methods set at 90% confidence level when a-path is zero across the range of b-path sizes and sample sizes. *Note.* 'BC' = bias-corrected bootstrap confidence interval, 'stBC' = significance-tested bias-corrected bootstrap confidence interval, 'rBC' = reduced bias-corrected bootstrap confidence interval, 'WBC' = 30% Winsorized bias-corrected bootstrap confidence interval, 'P' = percentile bootstrap confidence interval. The black horizontal line at .10 on the graphs represents the target type I error rate determined by the α-level of .10, and the grey shaded region indicates Bradley's liberal robustness criterion (.05 to .15). The x-axis is on the natural log scale.

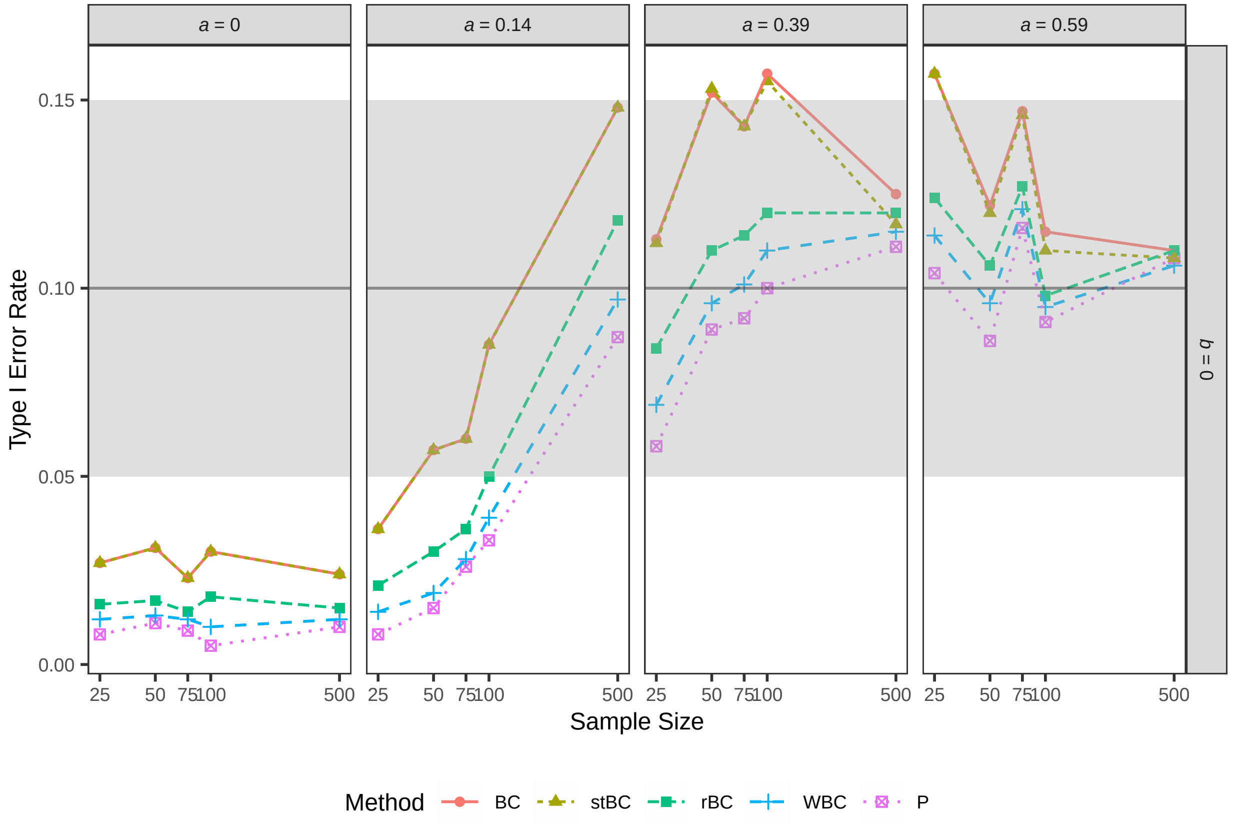

SM Figure 2. Type I error rate of all methods set at 90% confidence level when b-path is zero across the range of a-path sizes and sample sizes. *Note.* 'BC' = bias-corrected bootstrap confidence interval, 'stBC' = significance-tested bias-corrected bootstrap confidence interval, 'rBC' = reduced bias-corrected bootstrap confidence interval, 'WBC' = 30% Winsorized bias-corrected bootstrap confidence interval, 'P' = percentile bootstrap confidence interval. The black horizontal line at .10 on the graphs represents the target type I error rate determined by the α-level of .10, and the grey shaded region indicates Bradley's liberal robustness criterion (.05 to .15). The x-axis is on the natural log scale.

Confidence Level = 80%

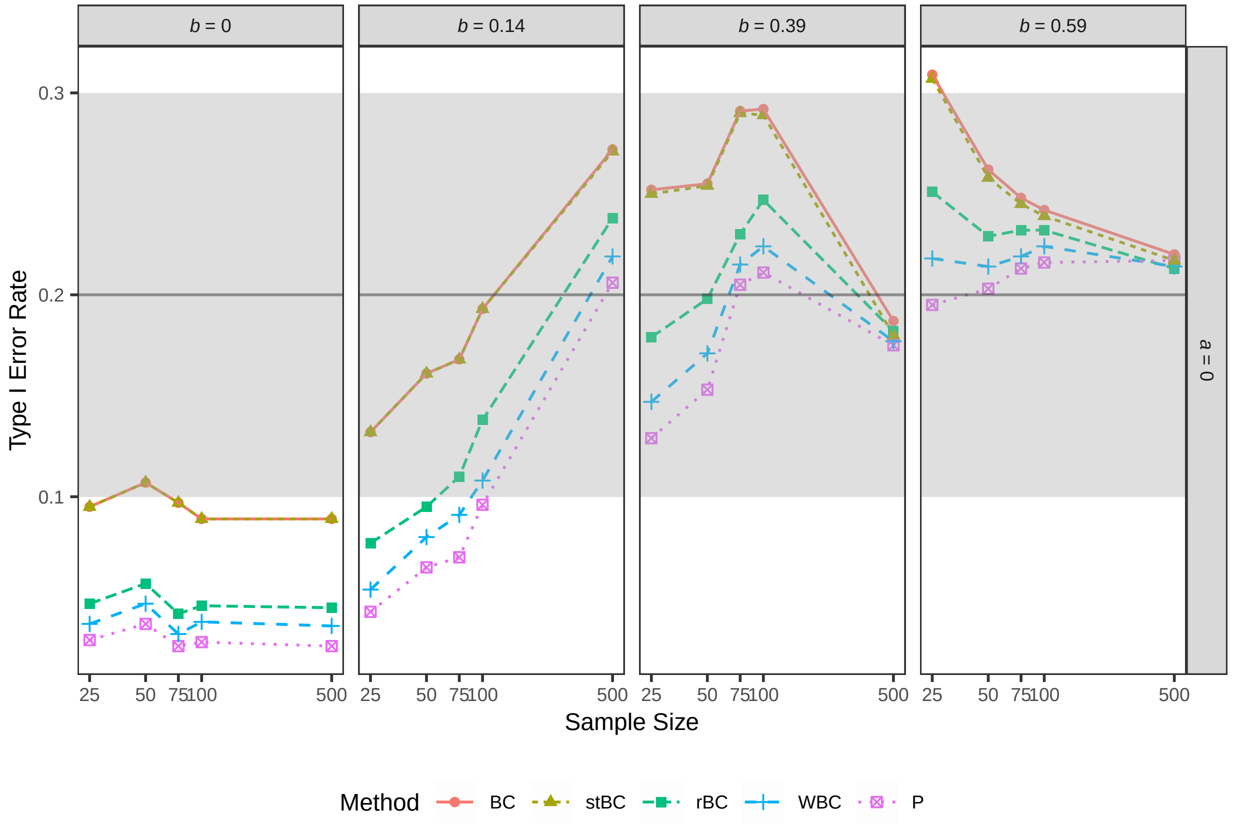

SM Figure 3. Type I error rate of all methods set at 80% confidence level when a-path is zero across the range of b-path sizes and sample sizes. *Note.* 'BC' = bias-corrected bootstrap confidence interval, 'stBC' = significance-tested bias-corrected bootstrap confidence interval, 'rBC' = reduced bias-corrected bootstrap confidence interval, 'WBC' = 30% Winsorized bias-corrected bootstrap confidence interval, 'P' = percentile bootstrap confidence interval. The black horizontal line at .20 on the graphs represents the target type I error rate determined by the α-level of .20, and the grey shaded region indicates Bradley's liberal robustness criterion (.10 to .30). The x-axis is on the natural log scale.

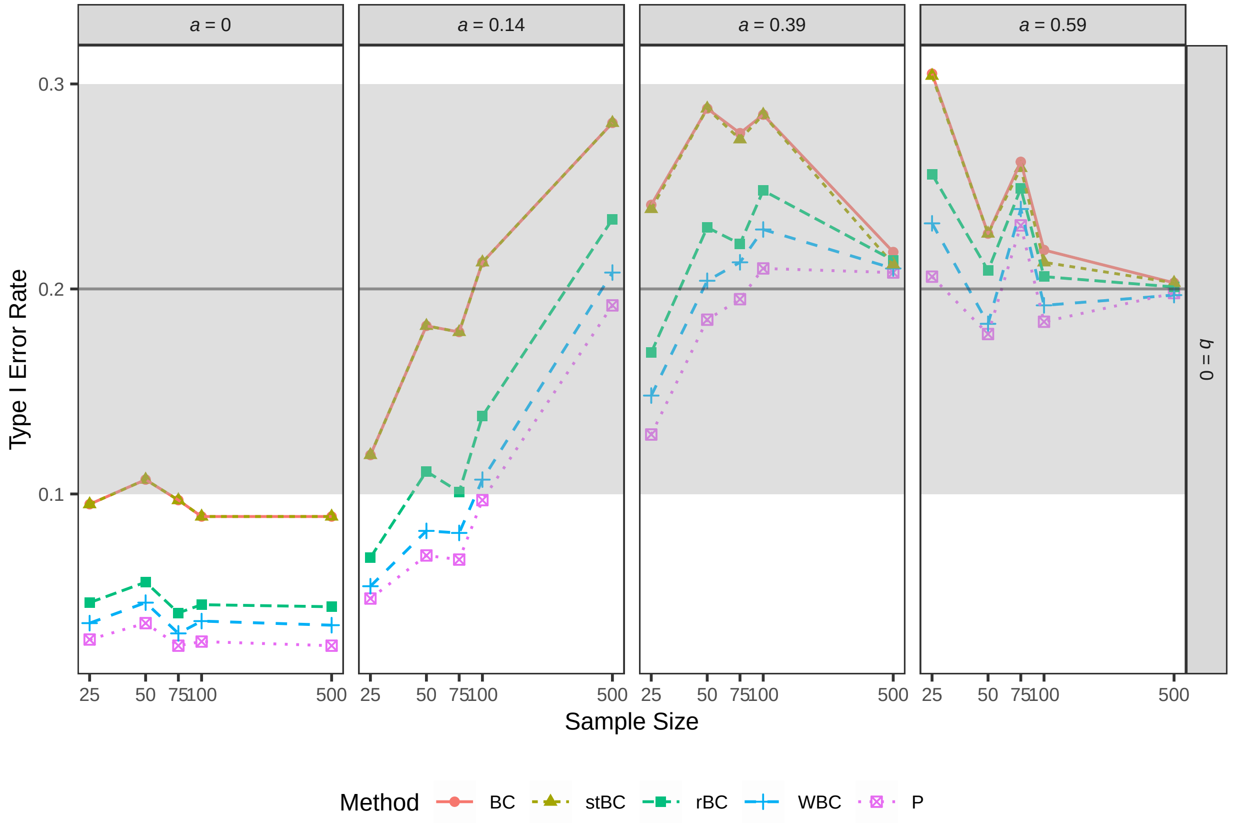

SM Figure 4. Type I error rate of all methods set at 80% confidence level when b-path is zero across the range of a-path sizes and sample sizes. *Note.* 'BC' = bias-corrected bootstrap confidence interval, 'stBC' = significance-tested bias-corrected bootstrap confidence interval, 'rBC' = reduced bias-corrected bootstrap confidence interval, 'WBC' = 30% Winsorized bias-corrected bootstrap confidence interval, 'P' = percentile bootstrap confidence interval. The black horizontal line at .20 on the graphs represents the target type I error rate determined by the α-level of .20, and the grey shaded region indicates Bradley's liberal robustness criterion (.10 to .30). The x-axis is on the natural log scale.

Power Graphs

Confidence Level = 95%

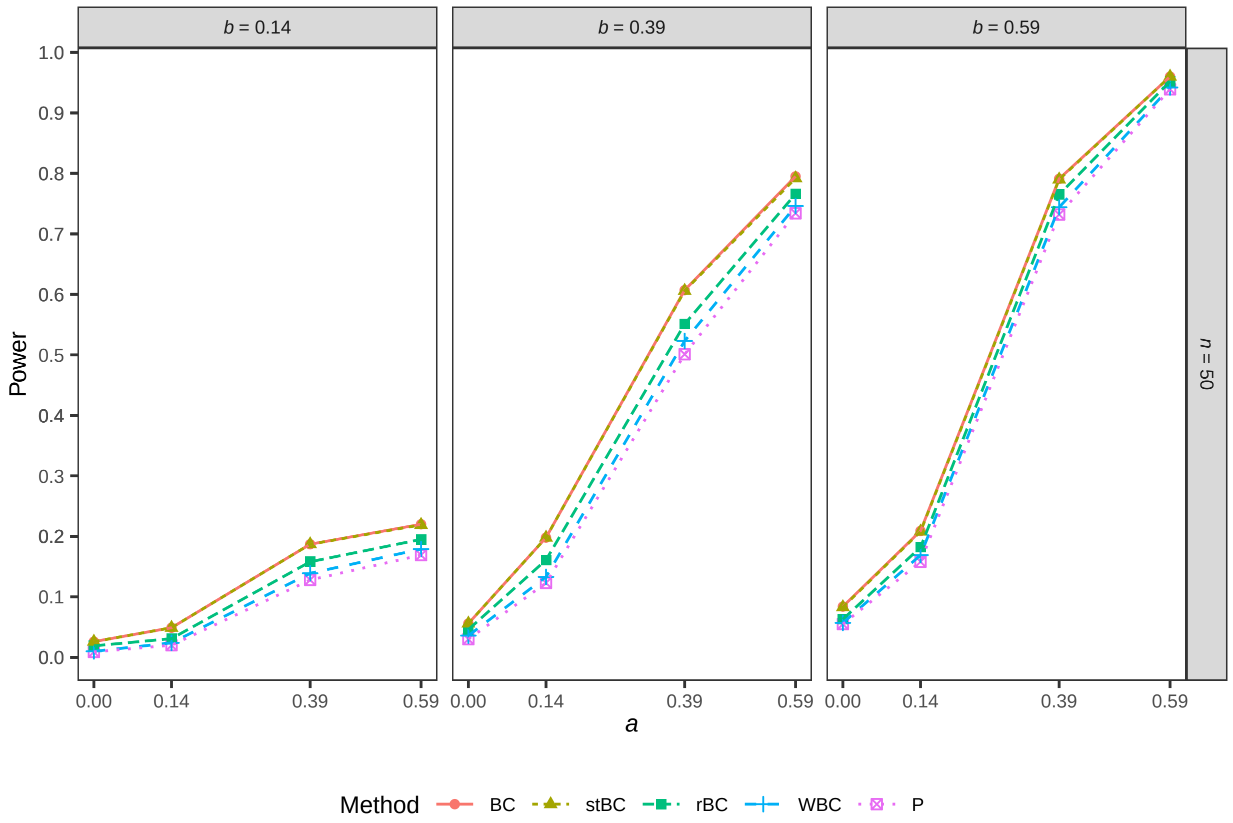

SM Figure 5. Power of all methods set at 95% confidence level when $n = 50$ across the range of b-path sizes and a-path sizes. *Note.* 'BC' = bias-corrected bootstrap confidence interval, 'stBC' = significance-tested bias-corrected bootstrap confidence interval, 'rBC' = reduced bias-corrected bootstrap confidence interval, 'WBC' = 30% Winsorized bias-corrected bootstrap confidence interval, 'P' = percentile bootstrap confidence interval.

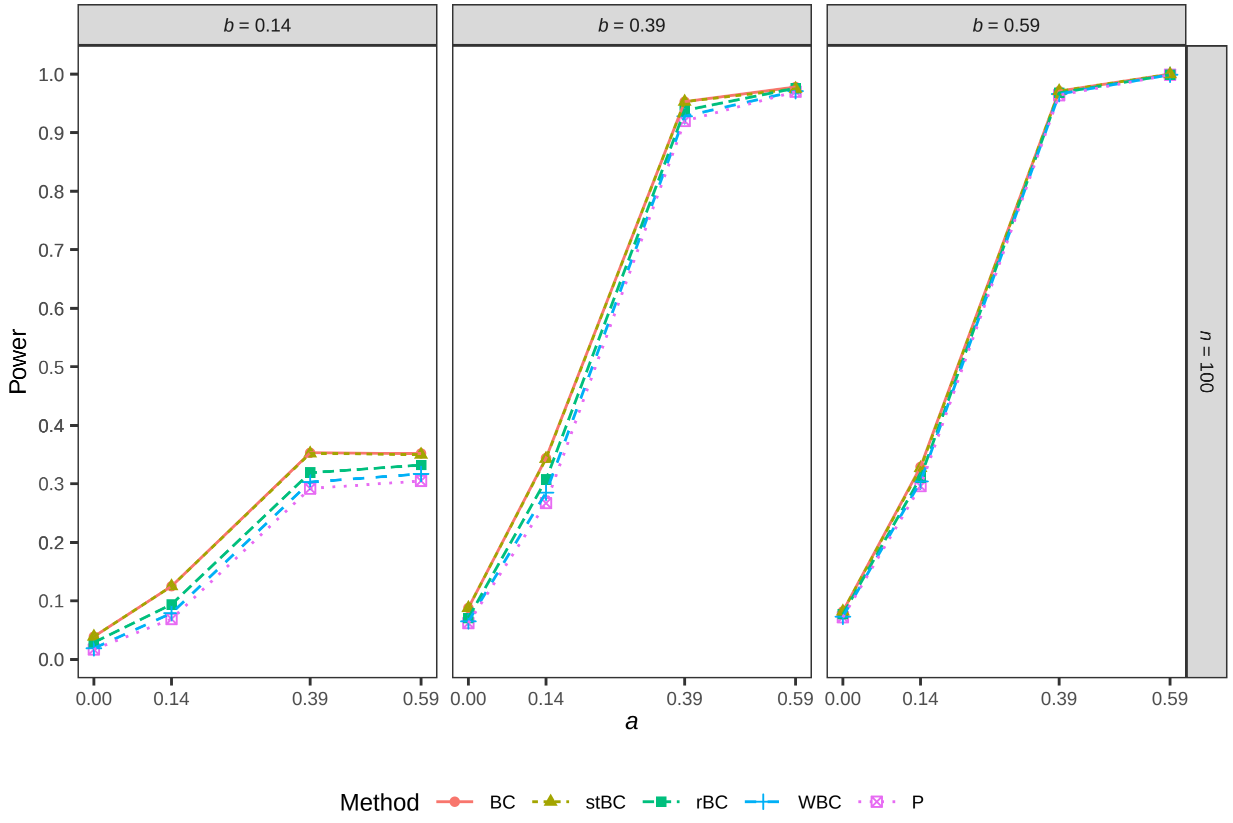

SM Figure 6. Power of all methods set at 95% confidence level when $n = 100$ across the range of b-path sizes and a-path sizes. *Note.* 'BC' = bias-corrected bootstrap confidence interval, 'stBC' = significance-tested bias-corrected bootstrap confidence interval, 'rBC' = reduced bias-corrected bootstrap confidence interval, 'WBC' = 30% Winsorized bias-corrected bootstrap confidence interval, 'P' = percentile bootstrap confidence interval.

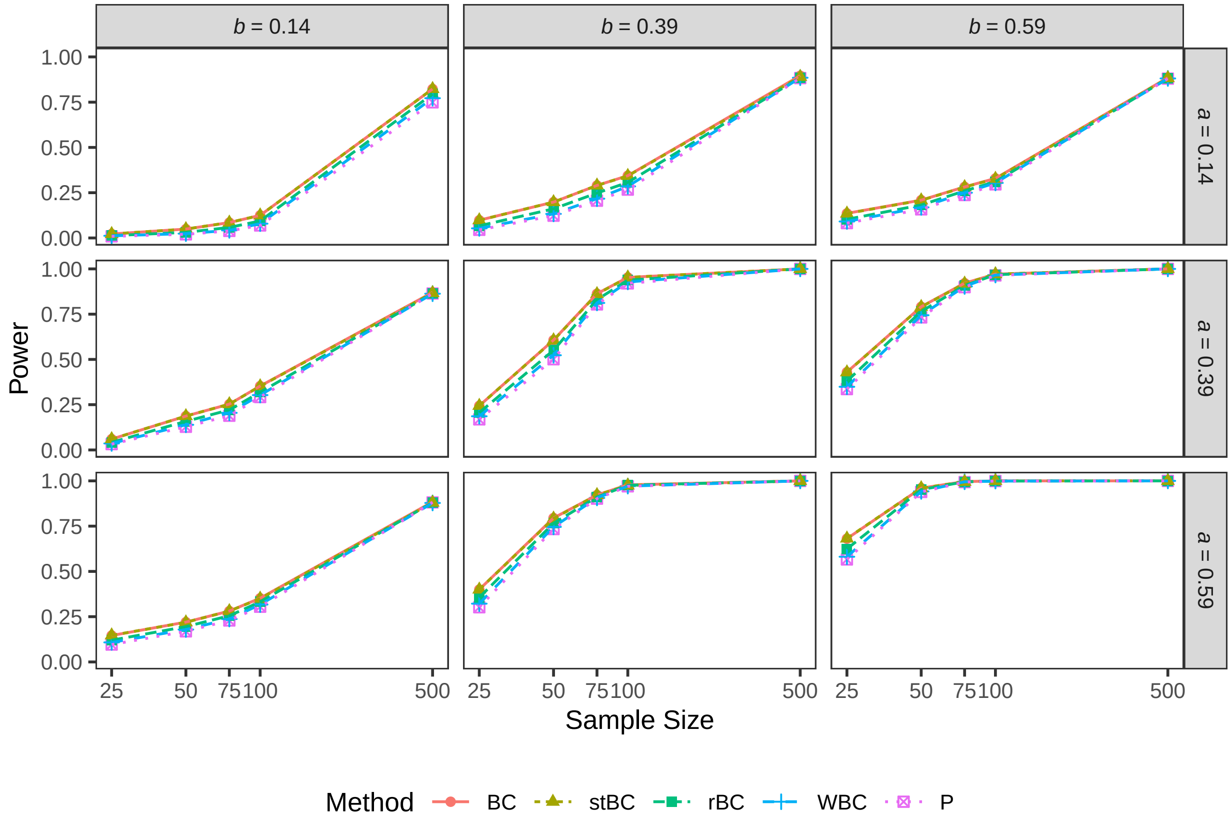

SM Figure 7. Chen and Fritz (2021)-style power graphs of all methods set at 95% confidence level across the range of a-path sizes, b-path sizes, and sample sizes. *Note.* 'BC' = bias-corrected bootstrap confidence interval, 'stBC' = significance-tested bias-corrected bootstrap confidence interval, 'rBC' = reduced bias-corrected bootstrap confidence interval, 'WBC' = 30% Winsorized bias-corrected bootstrap confidence interval, 'P' = percentile bootstrap confidence interval.

Confidence Level = 90%

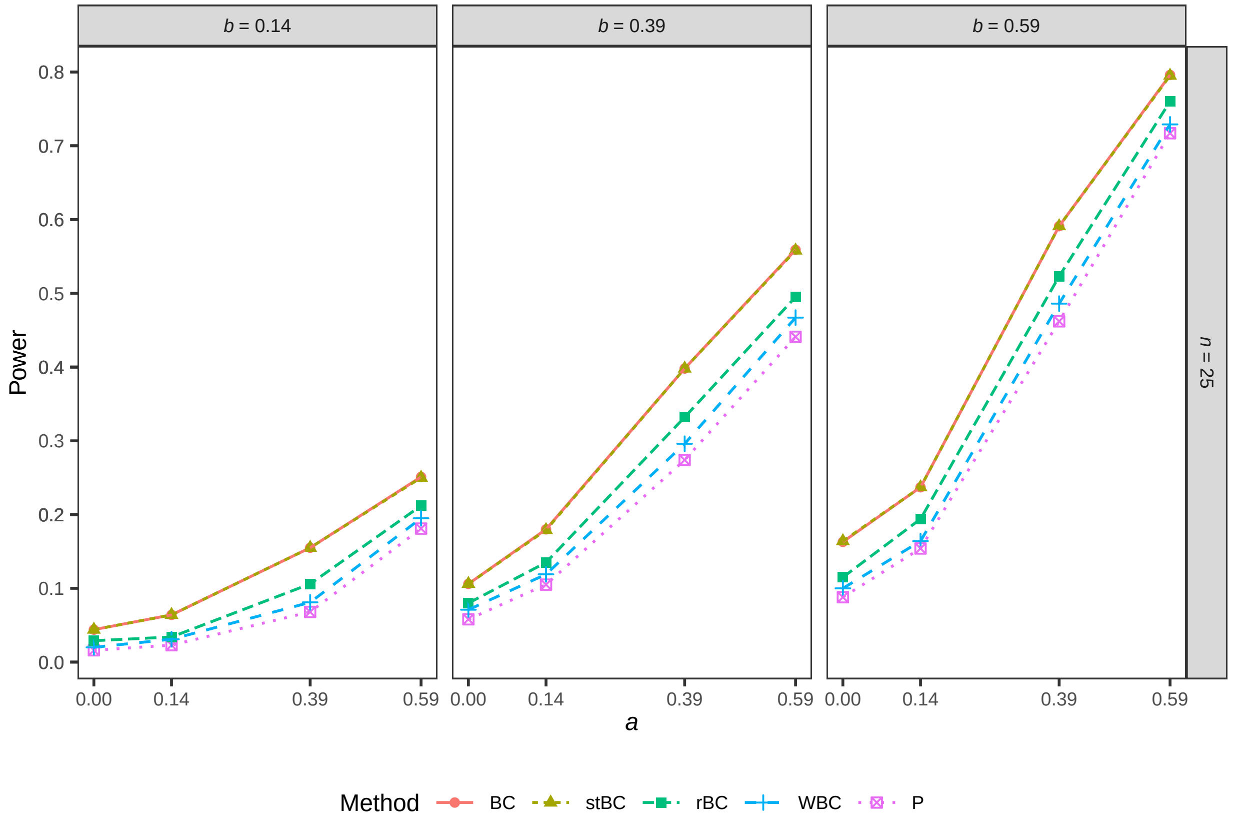

SM Figure 8. Power of all methods set at 90% confidence level when $n = 25$ across the range of b-path sizes and a-path sizes. *Note.* 'BC' = bias-corrected bootstrap confidence interval, 'stBC' = significance-tested bias-corrected bootstrap confidence interval, 'rBC' = reduced bias-corrected bootstrap confidence interval, 'WBC' = 30% Winsorized bias-corrected bootstrap confidence interval, 'P' = percentile bootstrap confidence interval.

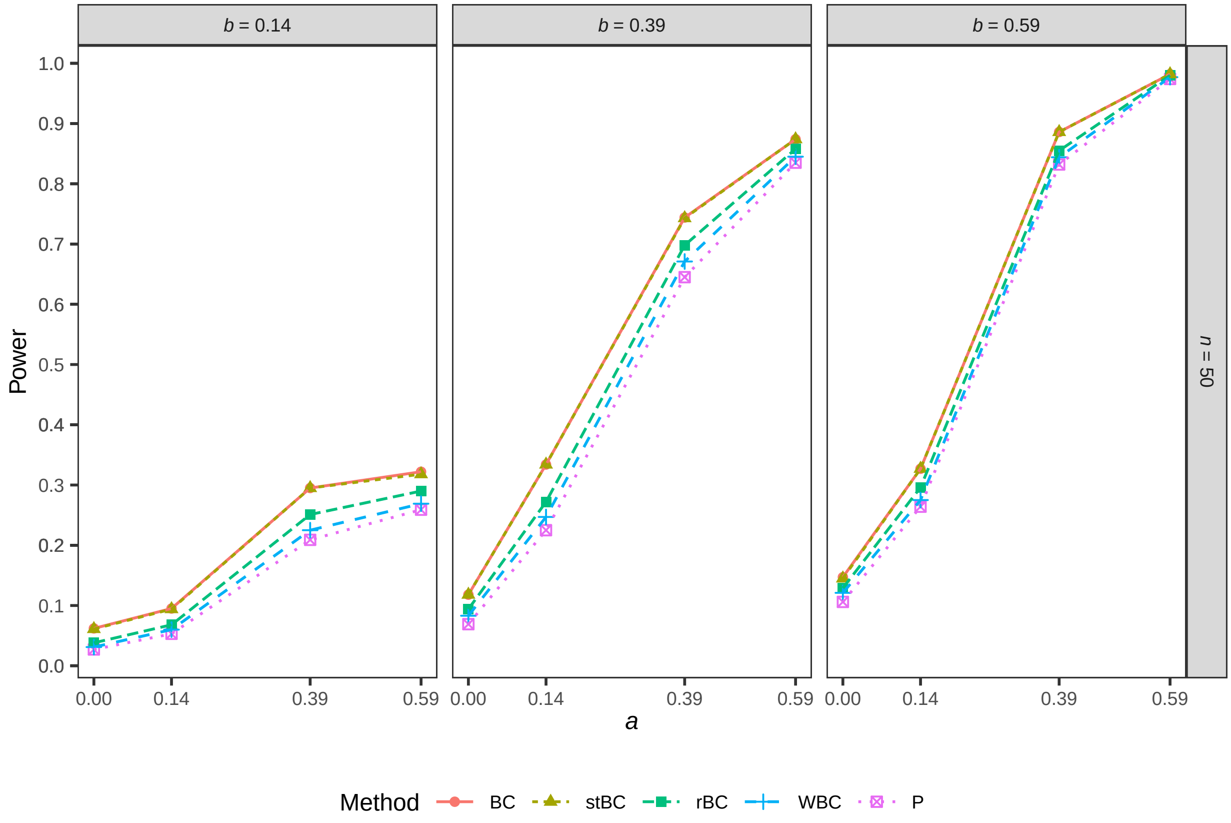

SM Figure 9. Power of all methods set at 90% confidence level when $n = 50$ across the range of b-path sizes and a-path sizes. *Note.* 'BC' = bias-corrected bootstrap confidence interval, 'stBC' = significance-tested bias-corrected bootstrap confidence interval, 'rBC' = reduced bias-corrected bootstrap confidence interval, 'WBC' = 30% Winsorized bias-corrected bootstrap confidence interval, 'P' = percentile bootstrap confidence interval.

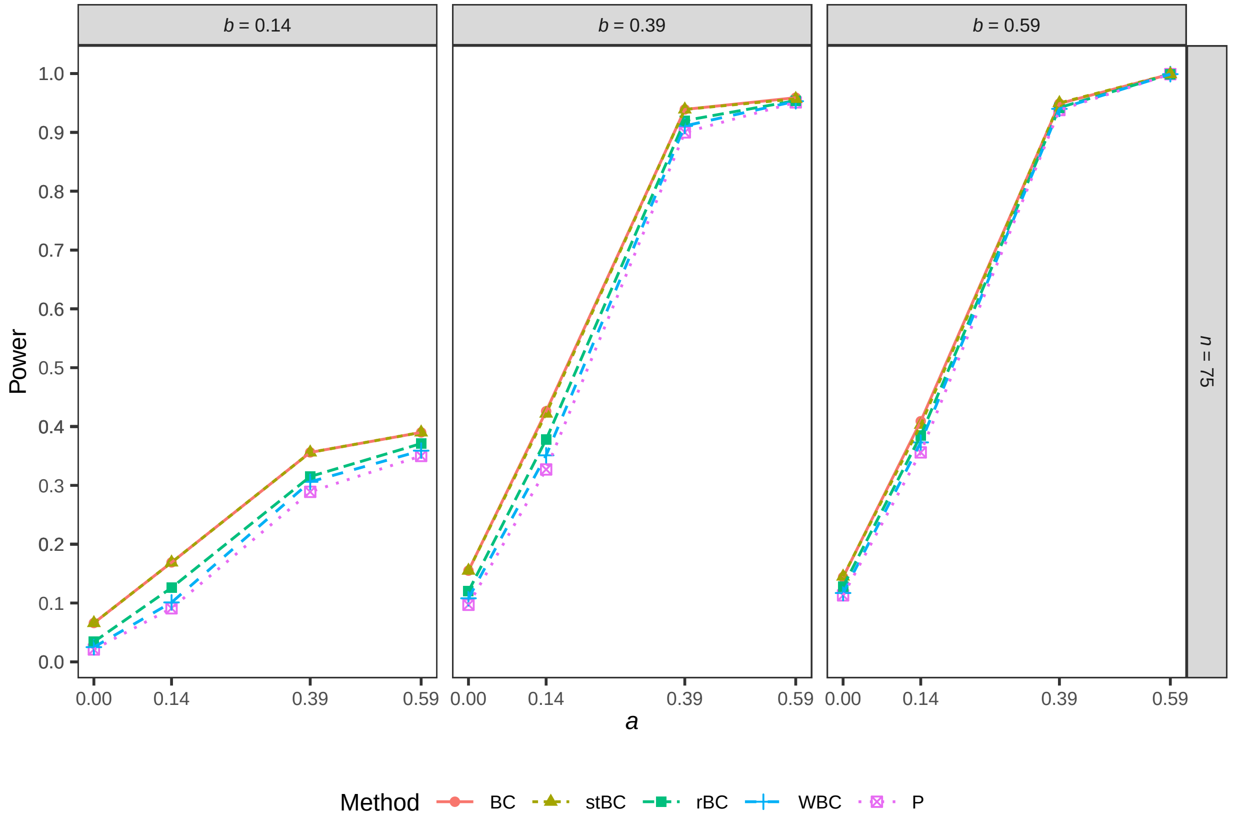

SM Figure 10. Power of all methods set at 90% confidence level when $n = 75$ across the range of b-path sizes and a-path sizes. *Note.* 'BC' = bias-corrected bootstrap confidence interval, 'stBC' = significance-tested bias-corrected bootstrap confidence interval, 'rBC' = reduced bias-corrected bootstrap confidence interval, 'WBC' = 30% Winsorized bias-corrected bootstrap confidence interval, 'P' = percentile bootstrap confidence interval.

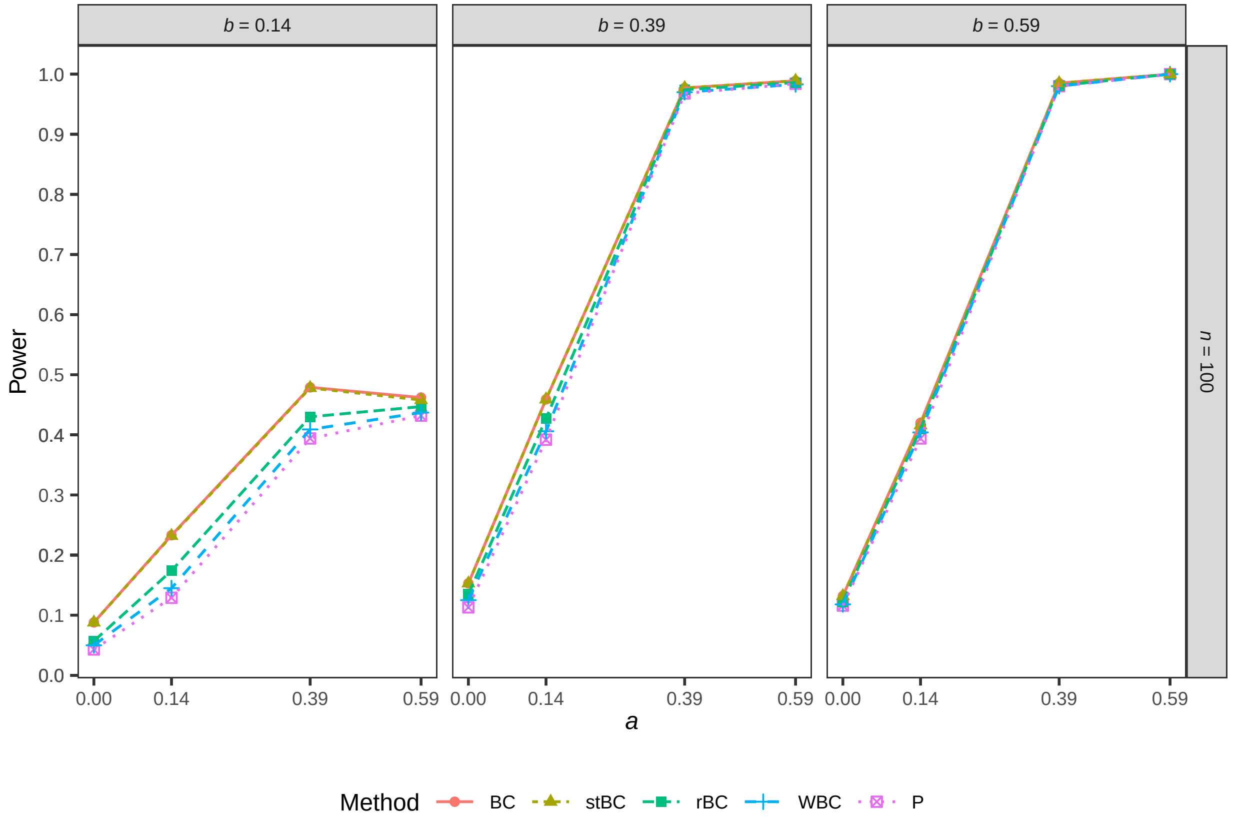

SM Figure 11. Power of all methods set at 90% confidence level when $n = 100$ across the range of b-path sizes and a-path sizes. *Note.* 'BC' = bias-corrected bootstrap confidence interval, 'stBC' = significance-tested bias-corrected bootstrap confidence interval, 'rBC' = reduced bias-corrected bootstrap confidence interval, 'WBC' = 30% Winsorized bias-corrected bootstrap confidence interval, 'P' = percentile bootstrap confidence interval.

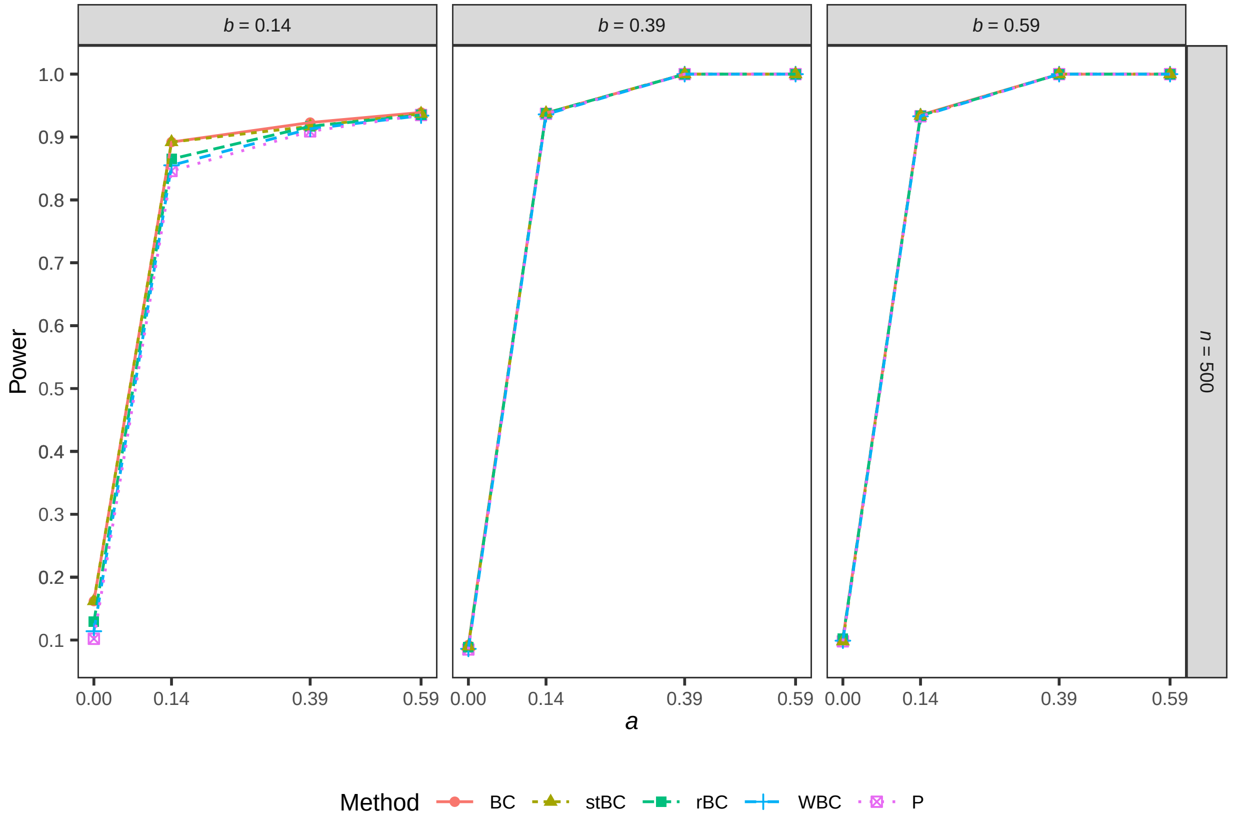

SM Figure 12. Power of all methods set at 90% confidence level when $n = 500$ across the range of b-path sizes and a-path sizes. *Note.* 'BC' = bias-corrected bootstrap confidence interval, 'stBC' = significance-tested bias-corrected bootstrap confidence interval, 'rBC' = reduced bias-corrected bootstrap confidence interval, 'WBC' = 30% Winsorized bias-corrected bootstrap confidence interval, 'P' = percentile bootstrap confidence interval.

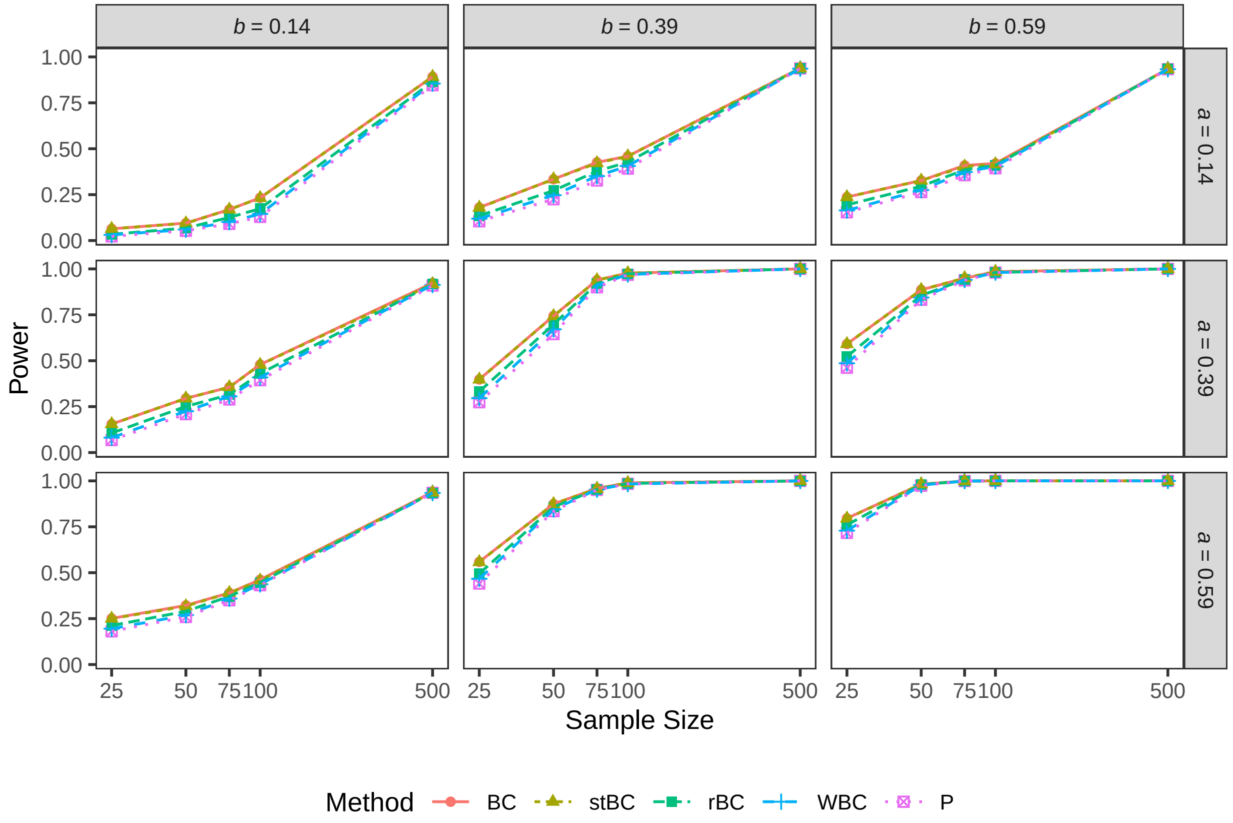

SM Figure 13. Chen and Fritz (2021)-style power graphs of all methods set at 90% confidence level across the range of *a*-path sizes, *b*-path sizes, and sample sizes. *Note.* 'BC' = bias-corrected bootstrap confidence interval, 'stBC' = significance-tested bias-corrected bootstrap confidence interval, 'rBC' = reduced bias-corrected bootstrap confidence interval, 'WBC' = 30% Winsorized bias-corrected bootstrap confidence interval, 'P' = percentile bootstrap confidence interval.

Confidence Level = 80%

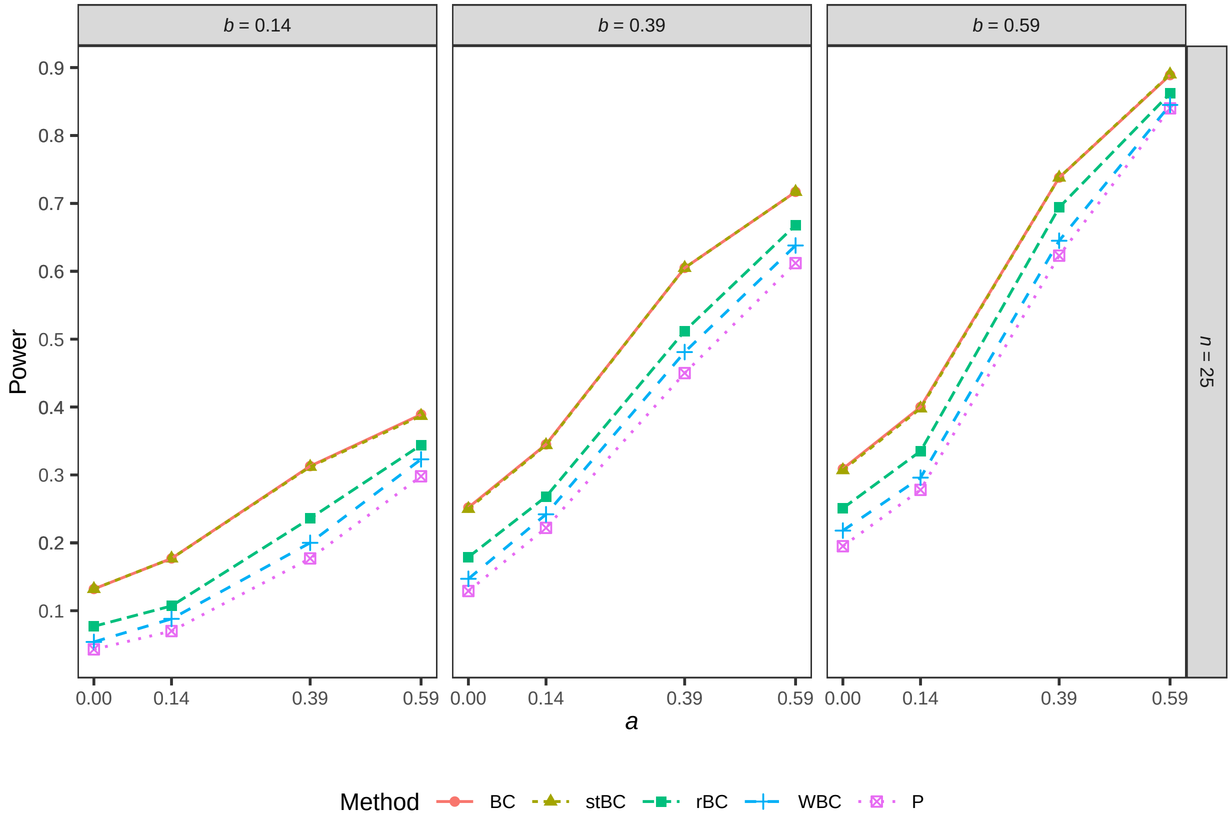

SM Figure 14. Power of all methods set at 80% confidence level when $n = 25$ across the range of b-path sizes and a-path sizes. *Note.* 'BC' = bias-corrected bootstrap confidence interval, 'stBC' = significance-tested bias-corrected bootstrap confidence interval, 'rBC' = reduced bias-corrected bootstrap confidence interval, 'WBC' = 30% Winsorized bias-corrected bootstrap confidence interval, 'P' = percentile bootstrap confidence interval.

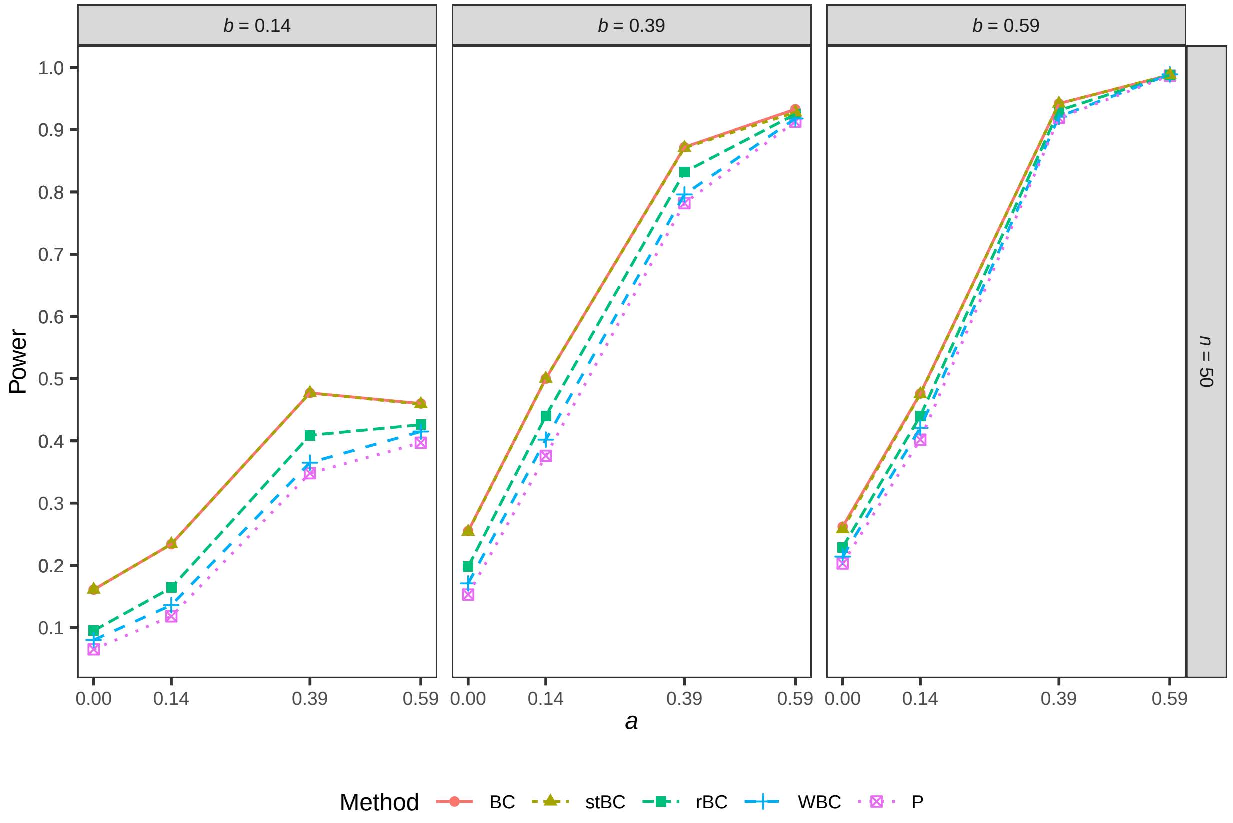

SM Figure 15. Power of all methods set at 80% confidence level when $n = 50$ across the range of b-path sizes and a-path sizes. *Note.* ‘BC’ = bias-corrected bootstrap confidence interval, ‘stBC’ = significance-tested bias-corrected bootstrap confidence interval, ‘rBC’ = reduced bias-corrected bootstrap confidence interval, ‘WBC’ = 30% Winsorized bias-corrected bootstrap confidence interval, ‘P’ = percentile bootstrap confidence interval.

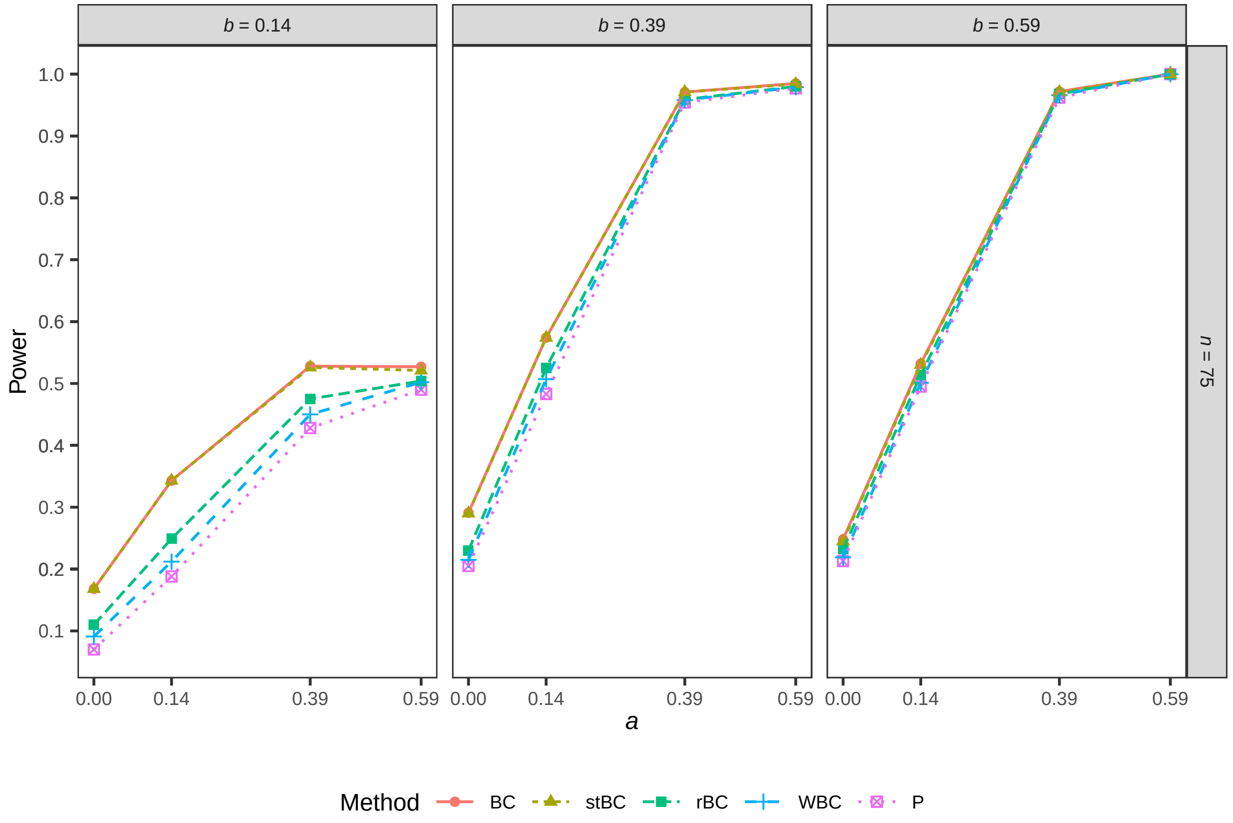

SM Figure 16. Power of all methods set at 80% confidence level when $n = 75$ across the range of b-path sizes and a-path sizes. *Note.* 'BC' = bias-corrected bootstrap confidence interval, 'stBC' = significance-tested bias-corrected bootstrap confidence interval, 'rBC' = reduced bias-corrected bootstrap confidence interval, 'WBC' = 30% Winsorized bias-corrected bootstrap confidence interval, 'P' = percentile bootstrap confidence interval.

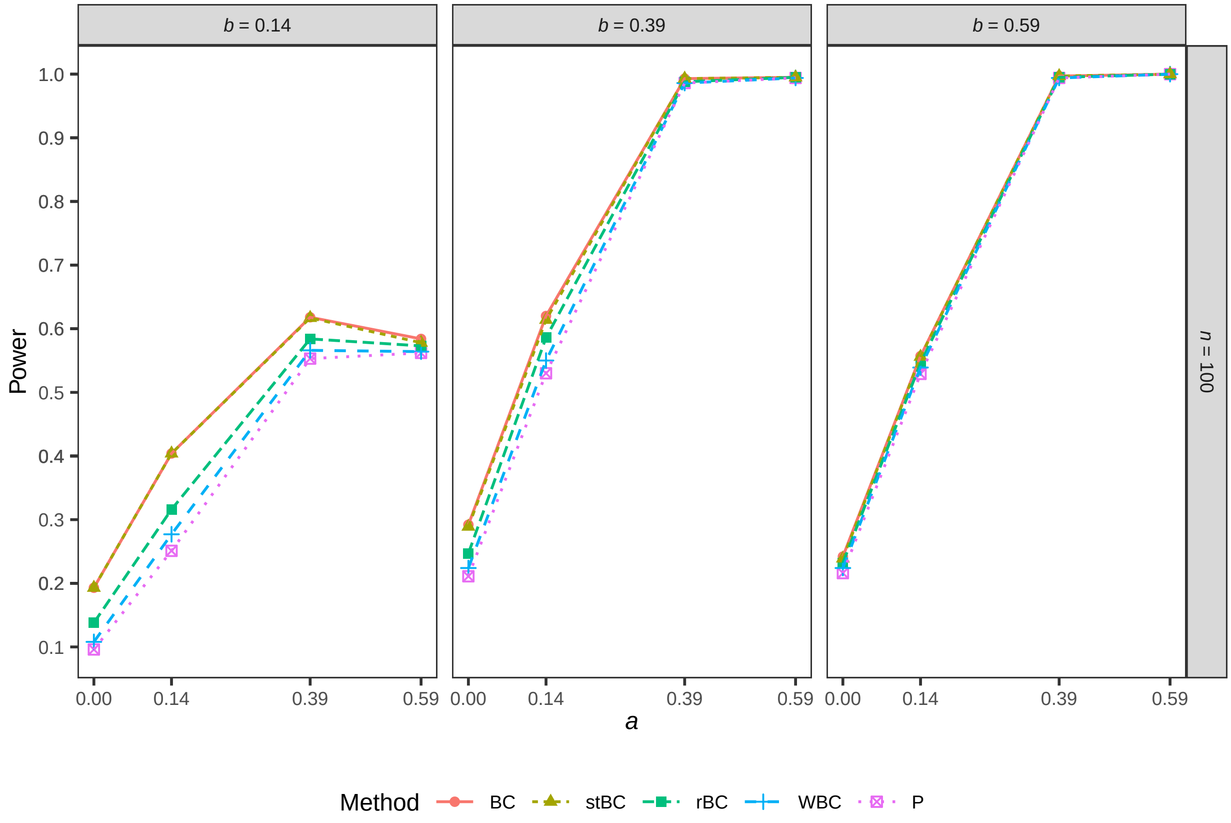

SM Figure 17. Power of all methods set at 80% confidence level when $n = 100$ across the range of b-path sizes and a-path sizes. *Note.* 'BC' = bias-corrected bootstrap confidence interval, 'stBC' = significance-tested bias-corrected bootstrap confidence interval, 'rBC' = reduced bias-corrected bootstrap confidence interval, 'WBC' = 30% Winsorized bias-corrected bootstrap confidence interval, 'P' = percentile bootstrap confidence interval.

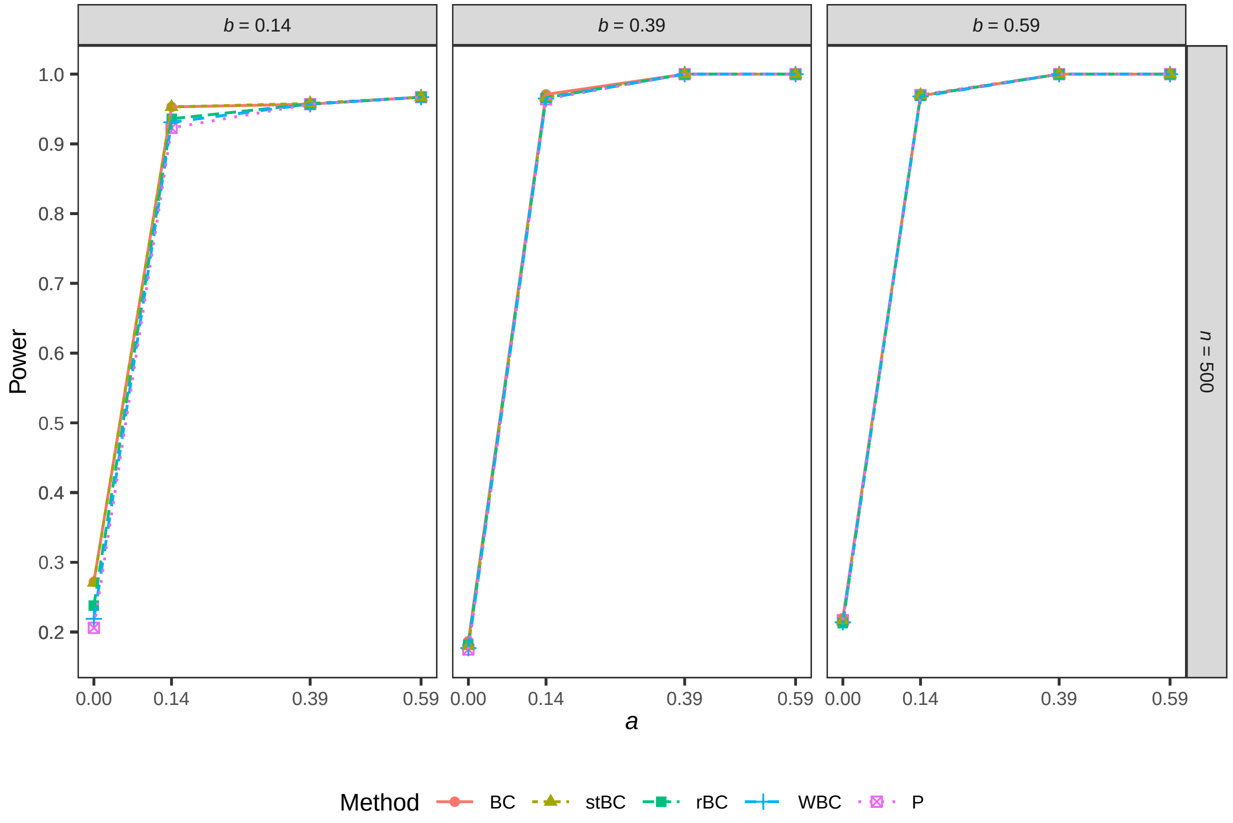

SM Figure 18. Power of all methods set at 80% confidence level when $n = 500$ across the range of b-path sizes and a-path sizes. *Note.* 'BC' = bias-corrected bootstrap confidence interval, 'stBC' = significance-tested bias-corrected bootstrap confidence interval, 'rBC' = reduced bias-corrected bootstrap confidence interval, 'WBC' = 30% Winsorized bias-corrected bootstrap confidence interval, 'P' = percentile bootstrap confidence interval.

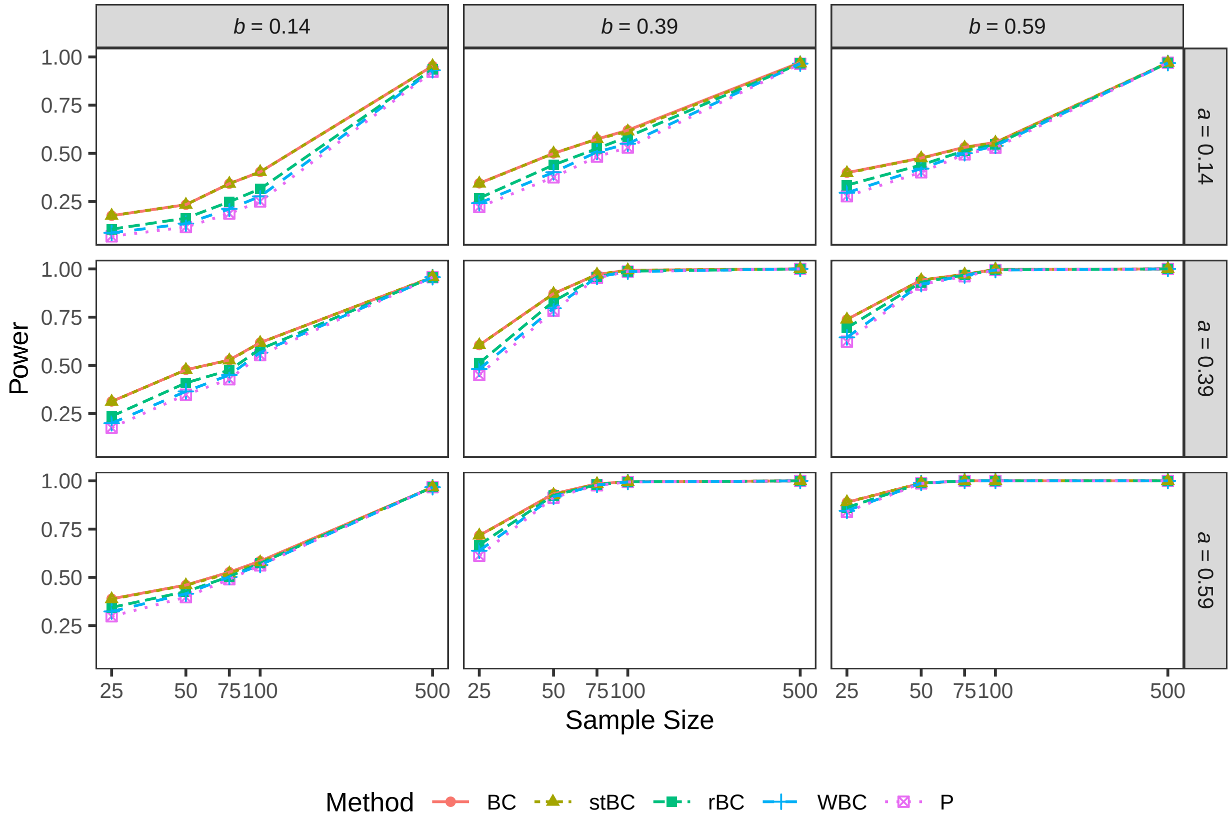

SM Figure 19. Chen and Fritz (2021)-style power graphs of all methods set at 80% confidence level across the range of a-path sizes, b-path sizes, and sample sizes. *Note.* 'BC' = bias-corrected bootstrap confidence interval, 'stBC' = significance-tested bias-corrected bootstrap confidence interval, 'rBC' = reduced bias-corrected bootstrap confidence interval, 'WBC' = 30% Winsorized bias-corrected bootstrap confidence interval, 'P' = percentile bootstrap confidence interval.

Balance Graphs

Confidence Level = 90%

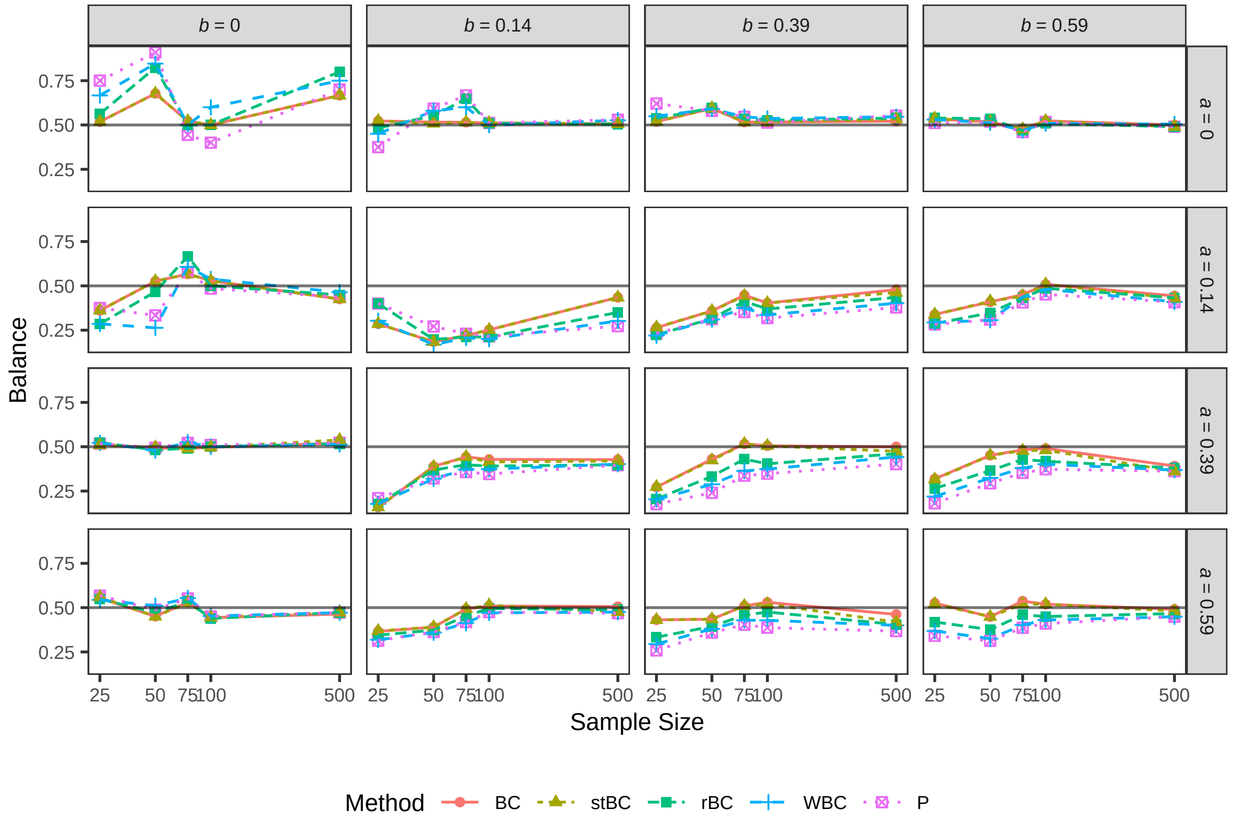

SM Figure 20. Balance of all methods set at 90% confidence level across the range of a-path sizes, b-path sizes, and sample sizes. *Note.* 'BC' = bias-corrected bootstrap confidence interval, 'stBC' = significance-tested bias-corrected bootstrap confidence interval, 'rBC' = reduced bias-corrected bootstrap confidence interval, 'WBC' = 30% Winsorized bias-corrected bootstrap confidence interval, 'P' = percentile bootstrap confidence interval. The black horizontal line at .50 on the graphs represents perfect balance. The x-axis is on the natural log scale.

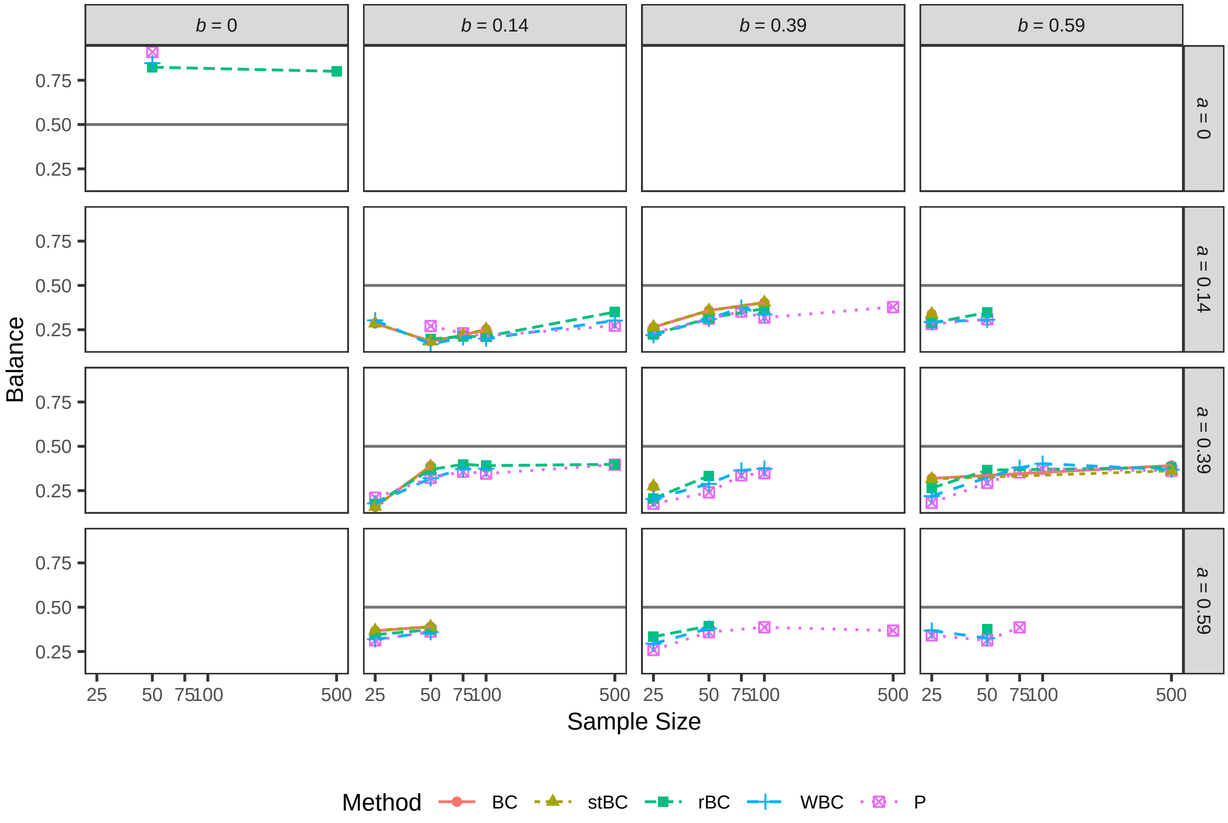

SM Figure 21. Balance of all methods set at 90% confidence level that are significant at $\alpha = 0.05$ across the range of a-path sizes, b-path sizes, and sample sizes. *Note.* 'BC' = bias-corrected bootstrap confidence interval, 'stBC' = significance-tested bias-corrected bootstrap confidence interval, 'rBC' = reduced bias-corrected bootstrap confidence interval, 'WBC' = 30% Winsorized bias-corrected bootstrap confidence interval, 'P' = percentile bootstrap confidence interval. The black horizontal line at .50 on the graphs represents the null hypothesis of perfect balance. The x-axis is on the natural log scale.

Confidence Level = 80%

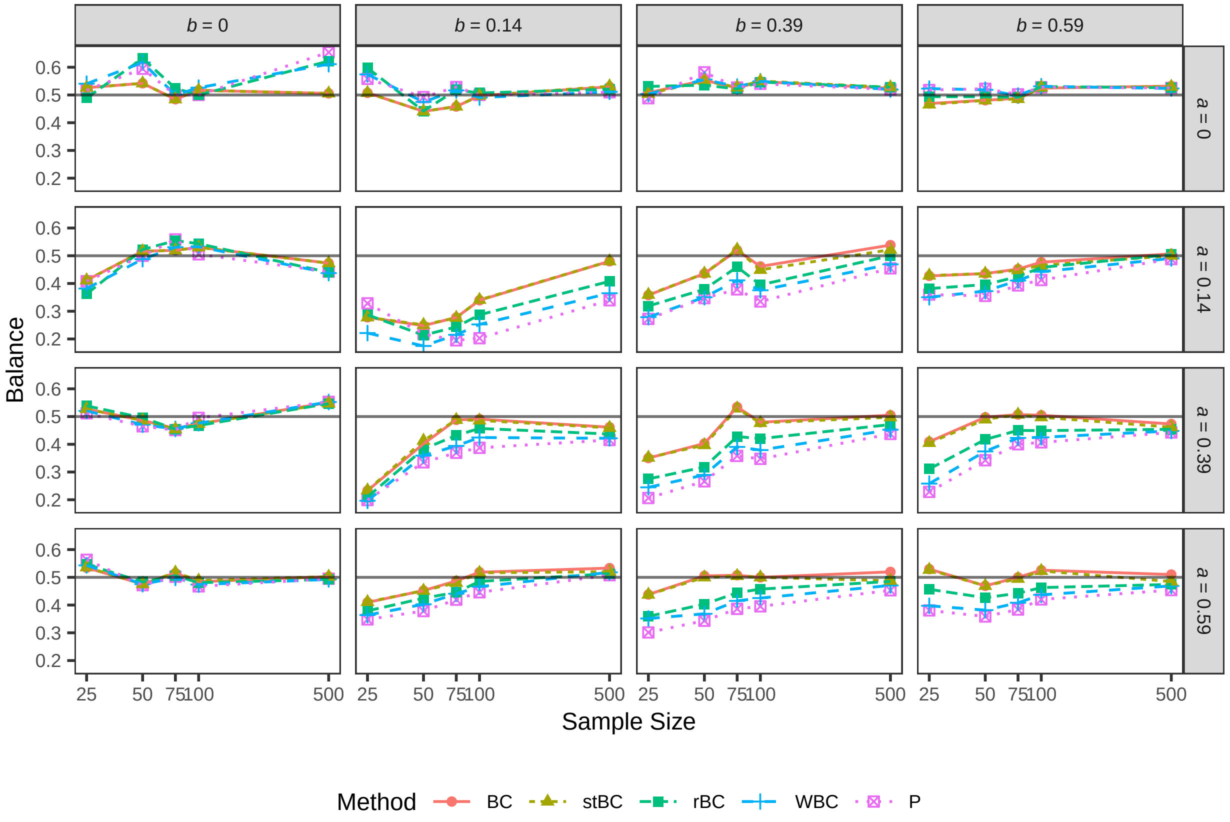

SM Figure 22. Balance of all methods set at 80% confidence level across the range of a-path sizes, b-path sizes, and sample sizes. *Note.* 'BC' = bias-corrected bootstrap confidence interval, 'stBC' = significance-tested bias-corrected bootstrap confidence interval, 'rBC' = reduced bias-corrected bootstrap confidence interval, 'WBC' = 30% Winsorized bias-corrected bootstrap confidence interval, 'P' = percentile bootstrap confidence interval. The black horizontal line at .50 on the graphs represents perfect balance. The x-axis is on the natural log scale.

```
## geom_path: Each group consists of only one observation. Do you need to adjust
## the group aesthetic?
```

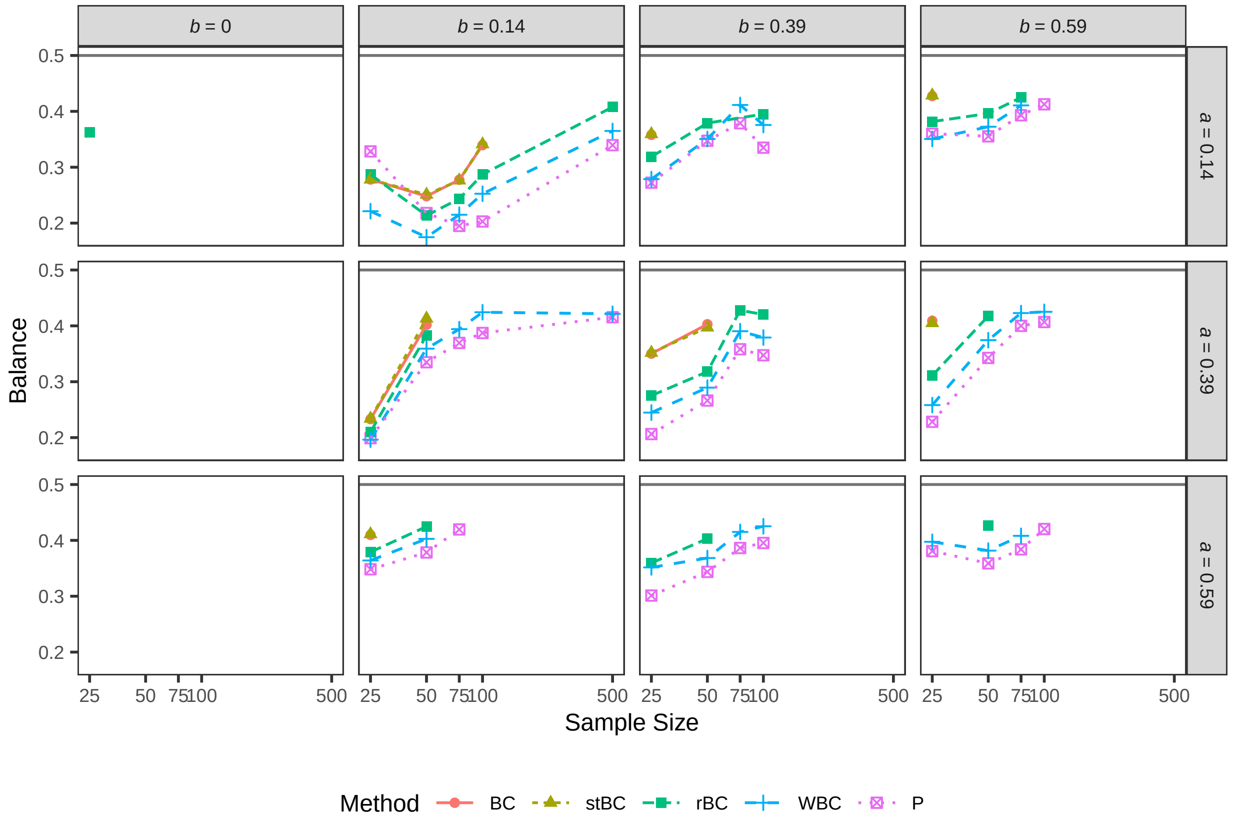

SM Figure 23. Balance of all methods set at 80% confidence level that are significant at $\alpha = 0.05$ across the range of a-path sizes, b-path sizes, and sample sizes. *Note.* 'BC' = bias-corrected bootstrap confidence interval, 'stBC' = significance-tested bias-corrected bootstrap confidence interval, 'rBC' = reduced bias-corrected bootstrap confidence interval, 'WBC' = 30% Winsorized bias-corrected bootstrap confidence interval, 'P' = percentile bootstrap confidence interval. The black horizontal line at .50 on the graphs represents the null hypothesis of perfect balance. The x-axis is on the natural log scale.

Power/Balance Graphs Controlling for Type I Error Rate

Confidence Level = 95%

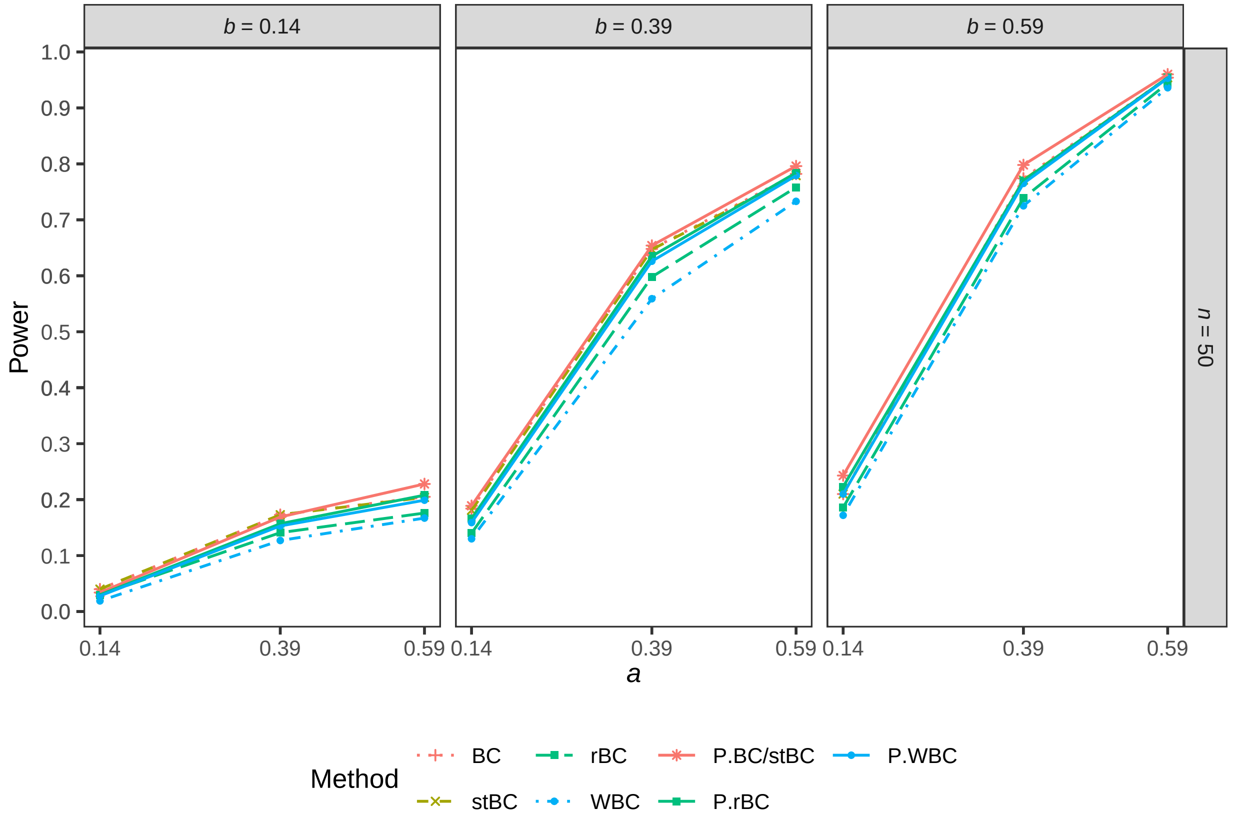

SM Figure 24. Power of all methods set at 95% confidence level compared to PBCI controlling for type I error rate when $n = 50$ across the range of b-path sizes and a-path sizes. *Note.* 'BC' = bias-corrected bootstrap confidence interval, 'stBC' = significance-tested bias-corrected bootstrap confidence interval, 'rBC' = reduced bias-corrected bootstrap confidence interval, 'WBC' = 30% Winsorized bias-corrected bootstrap confidence interval, 'P.BC/stBC' = comparison percentile bootstrap confidence interval for BC and stBC, 'P.rBC' = comparison percentile bootstrap confidence interval for rBC, 'P.WBC' = comparison percentile bootstrap confidence interval for WBC.

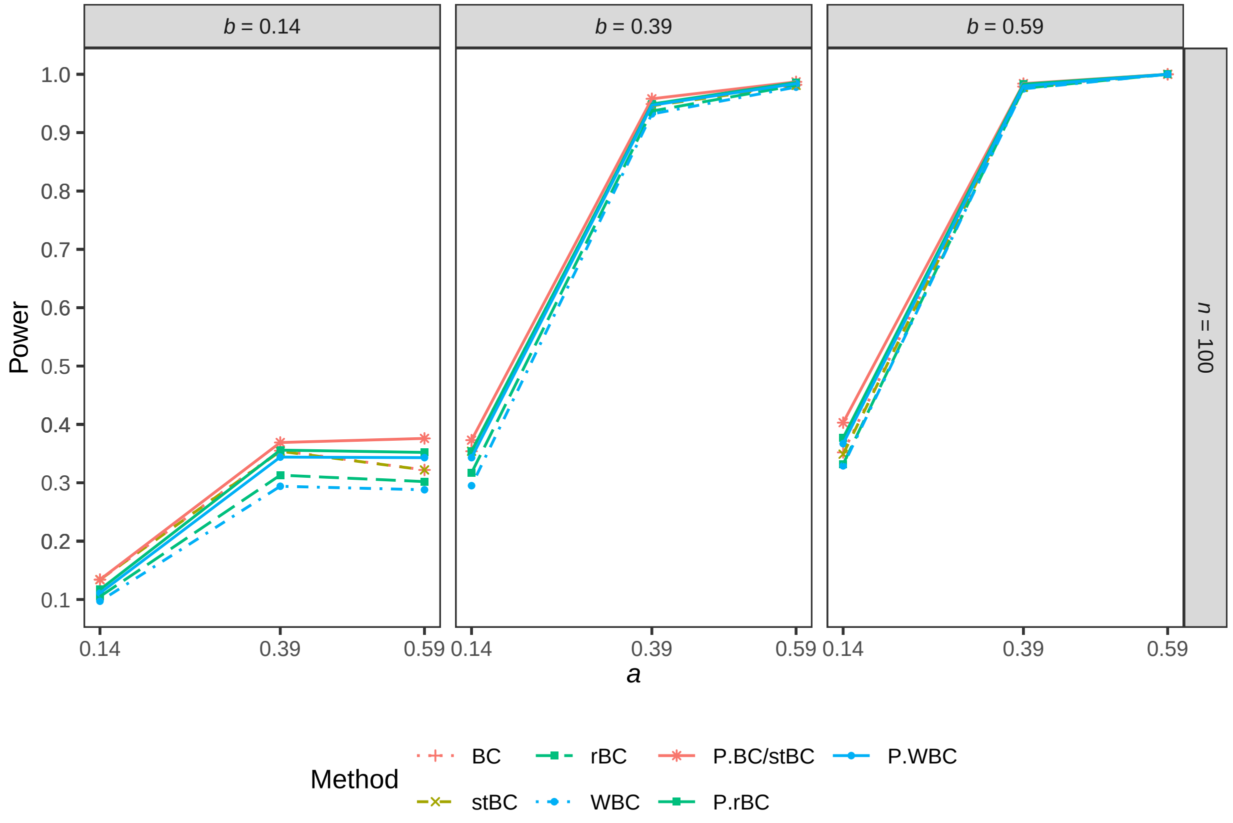

SM Figure 25. Power of all methods set at 95% confidence level compared to PBCI controlling for type I error rate when n = 100 across the range of b-path sizes and a-path sizes. *Note.* 'BC' = bias-corrected bootstrap confidence interval, 'stBC' = significance-tested bias-corrected bootstrap confidence interval, 'rBC' = reduced bias-corrected bootstrap confidence interval, 'WBC' = 30% Winsorized bias-corrected bootstrap confidence interval, 'P.BC/stBC' = comparison percentile bootstrap confidence interval for BC and stBC, 'P.rBC' = comparison percentile bootstrap confidence interval for rBC, 'P.WBC' = comparison percentile bootstrap confidence interval for WBC.

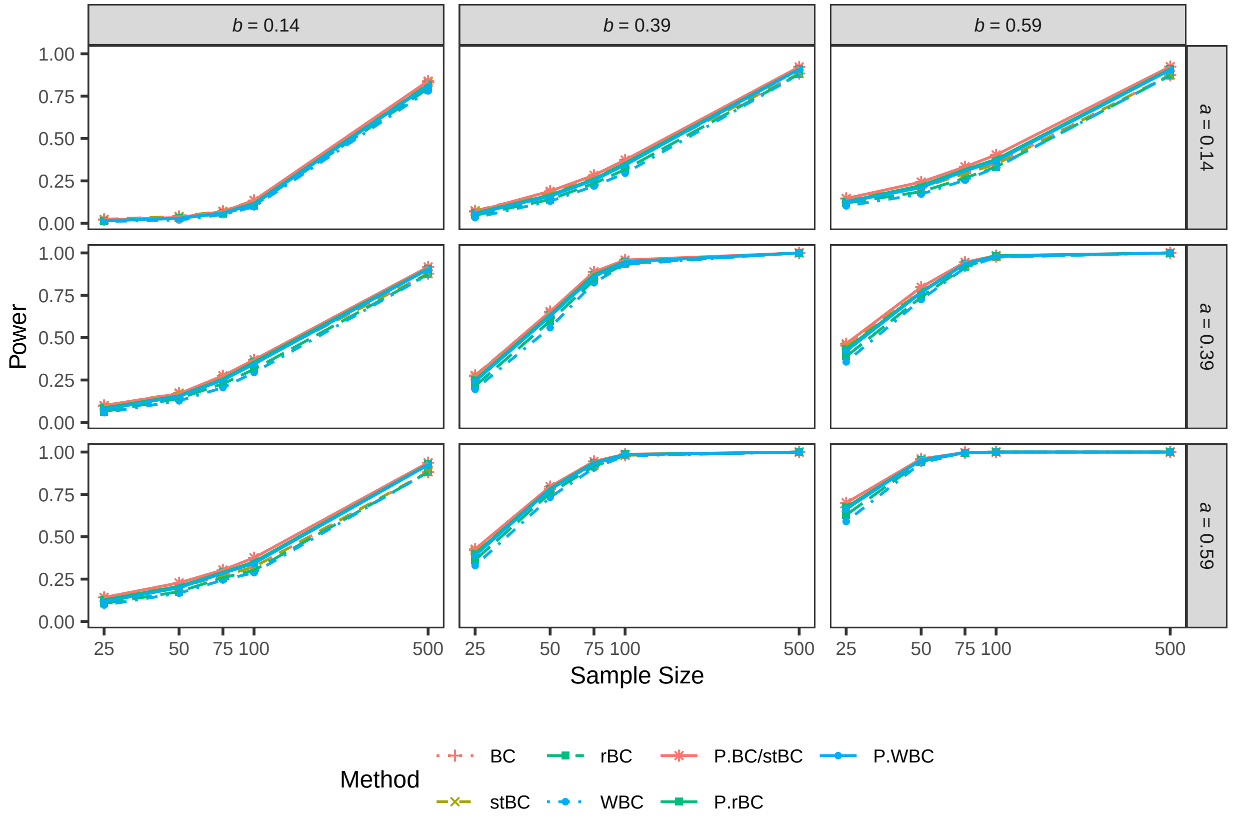

SM Figure 26. Chen and Fritz (2021)-style power graphs of all methods set at 95% confidence level compared to PBCI controlling for type I error rate across the range of a-path sizes, b-path sizes, and sample sizes. *Note.* 'BC' = bias-corrected bootstrap confidence interval, 'stBC' = significance-tested bias-corrected bootstrap confidence interval, 'rBC' = reduced bias-corrected bootstrap confidence interval, 'WBC' = 30% Winsorized bias-corrected bootstrap confidence interval, 'P.BC/stBC' = comparison percentile bootstrap confidence interval for BC and stBC, 'P.rBC' = comparison percentile bootstrap confidence interval for rBC, 'P.WBC' = comparison percentile bootstrap confidence interval for WBC.

```
##
##       BCBCI  BCBCI.PBCI        PBCI      rBCBCI rBCBCI.PBCI     stBCBCI
##          16          34          25          18          31          17
##      WBCBCI WBCBCI.PBCI
##          26          31

## geom_path: Each group consists of only one observation. Do you need to adjust
## the group aesthetic?
```

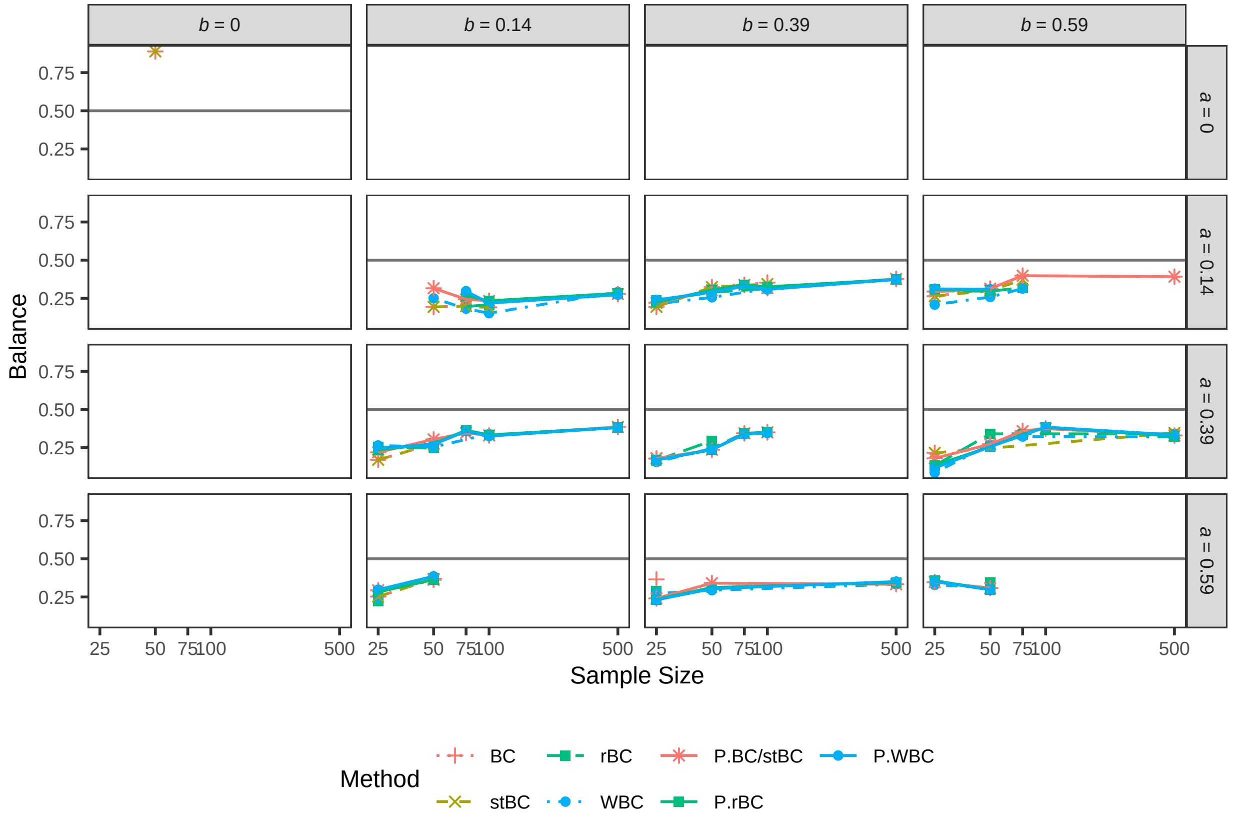

SM Figure 27. Balance of all methods set at 95% confidence level compared to PBCI controlling for type I error rate that are significant at $\alpha = 0.05$ across the range of a-path sizes, b-path sizes, and sample sizes. *Note.* 'BC' = bias-corrected bootstrap confidence interval, 'stBC' = significance-tested bias-corrected bootstrap confidence interval, 'rBC' = reduced bias-corrected bootstrap confidence interval, 'WBC' = 30% Winsorized bias-corrected bootstrap confidence interval, 'P' = percentile bootstrap confidence interval. The black horizontal line at .50 on the graphs represents the null hypothesis of perfect balance. The x-axis is on the natural log scale. *Note.* 'BC' = bias-corrected bootstrap confidence interval, 'stBC' = significance-tested bias-corrected bootstrap confidence interval, 'rBC' = reduced bias-corrected bootstrap confidence interval, 'WBC' = 30% Winsorized bias-corrected bootstrap confidence interval, 'P.BC/stBC' = comparison percentile bootstrap confidence interval for BC and stBC, 'P.rBC' = comparison percentile bootstrap confidence interval for rBC, 'P.WBC' = comparison percentile bootstrap confidence interval for WBC.

Coverage Graphs

Confidence Level = 95%

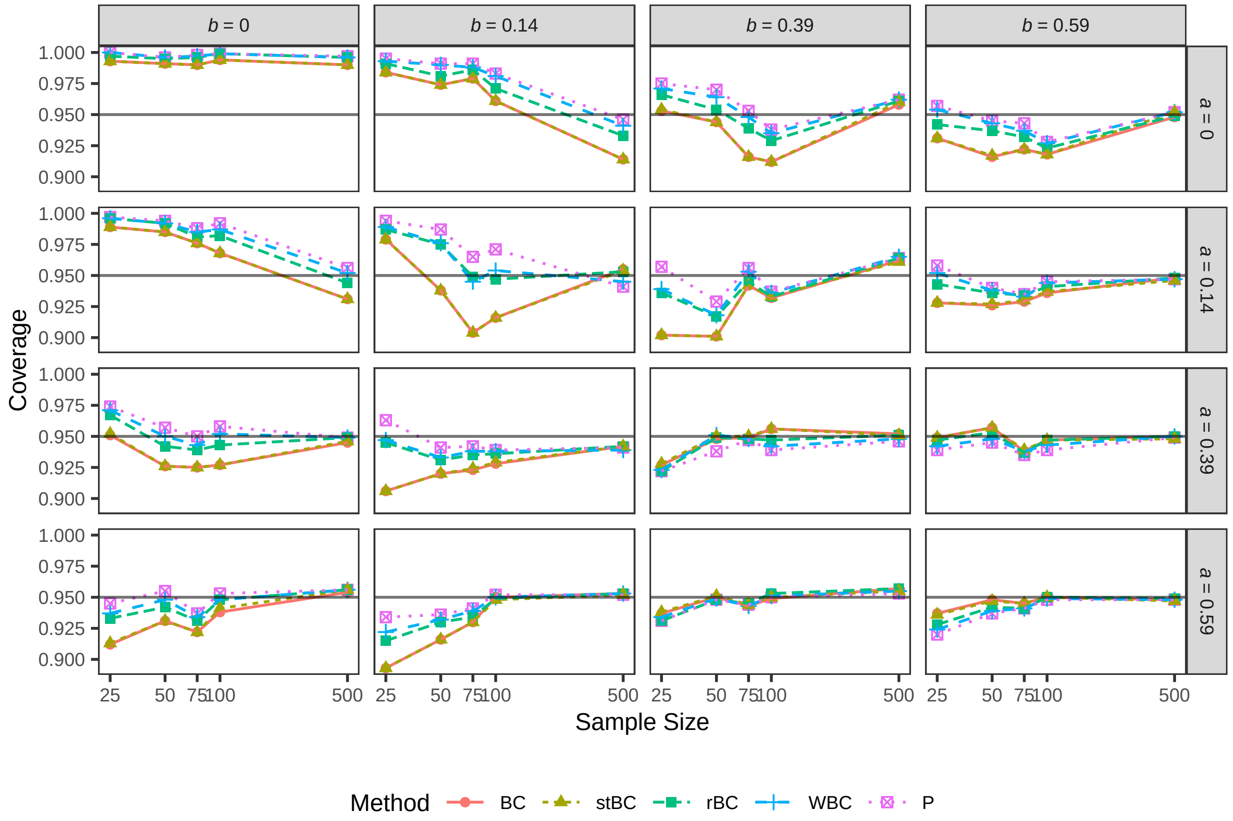

SM Figure 28. Coverage of all methods set at 95% confidence level across the range of a-path sizes, b-path sizes, and sample sizes. *Note.* 'BC' = bias-corrected bootstrap confidence interval, 'stBC' = significance-tested bias-corrected bootstrap confidence interval, 'rBC' = reduced bias-corrected bootstrap confidence interval, 'WBC' = 30% Winsorized bias-corrected bootstrap confidence interval, 'P' = percentile bootstrap confidence interval. The black horizontal line at .95 on the graphs represents target coverage. The x-axis is on the natural log scale.

Confidence Level = 90%

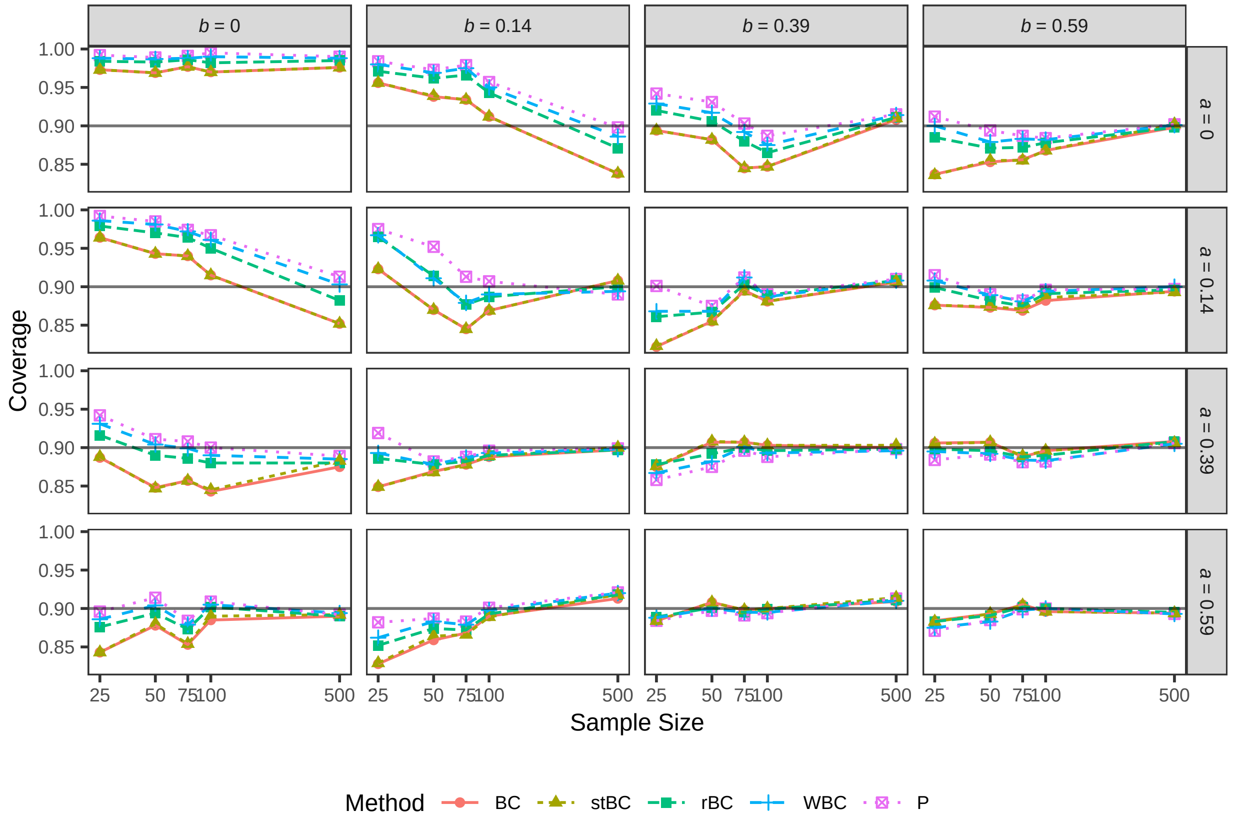

SM Figure 29. Coverage of all methods set at 90% confidence level across the range of a-path sizes, b-path sizes, and sample sizes. *Note.* 'BC' = bias-corrected bootstrap confidence interval, 'stBC' = significance-tested bias-corrected bootstrap confidence interval, 'rBC' = reduced bias-corrected bootstrap confidence interval, 'WBC' = 30% Winsorized bias-corrected bootstrap confidence interval, 'P' = percentile bootstrap confidence interval. The black horizontal line at .90 on the graphs represents target coverage. The x-axis is on the natural log scale.

Confidence Level = 80%

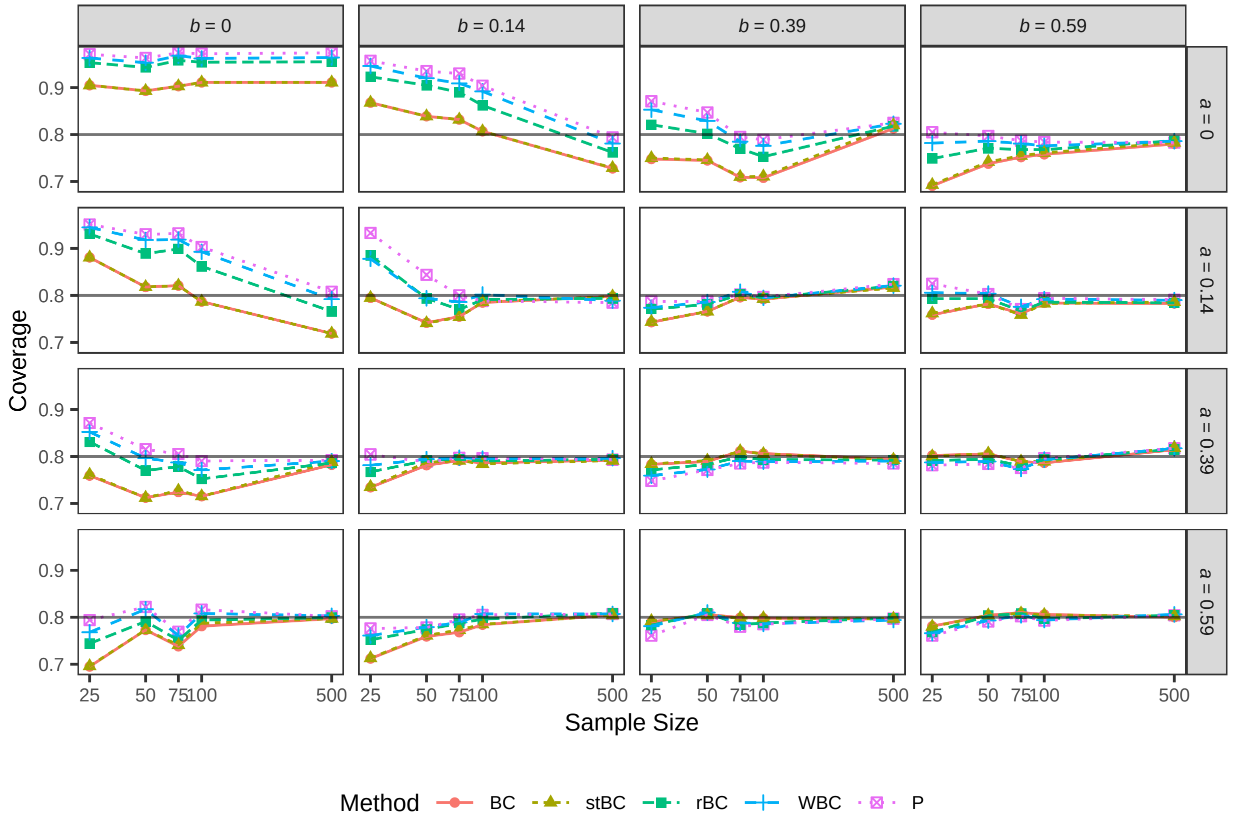

SM Figure 30. Coverage of all methods set at 80% confidence level across the range of a-path sizes, b-path sizes, and sample sizes. *Note.* 'BC' = bias-corrected bootstrap confidence interval, 'stBC' = significance-tested bias-corrected bootstrap confidence interval, 'rBC' = reduced bias-corrected bootstrap confidence interval, 'WBC' = 30% Winsorized bias-corrected bootstrap confidence interval, 'P' = percentile bootstrap confidence interval. The black horizontal line at .80 on the graphs represents target coverage. The x-axis is on the natural log scale.

Width Graphs

Confidence Level = 95%

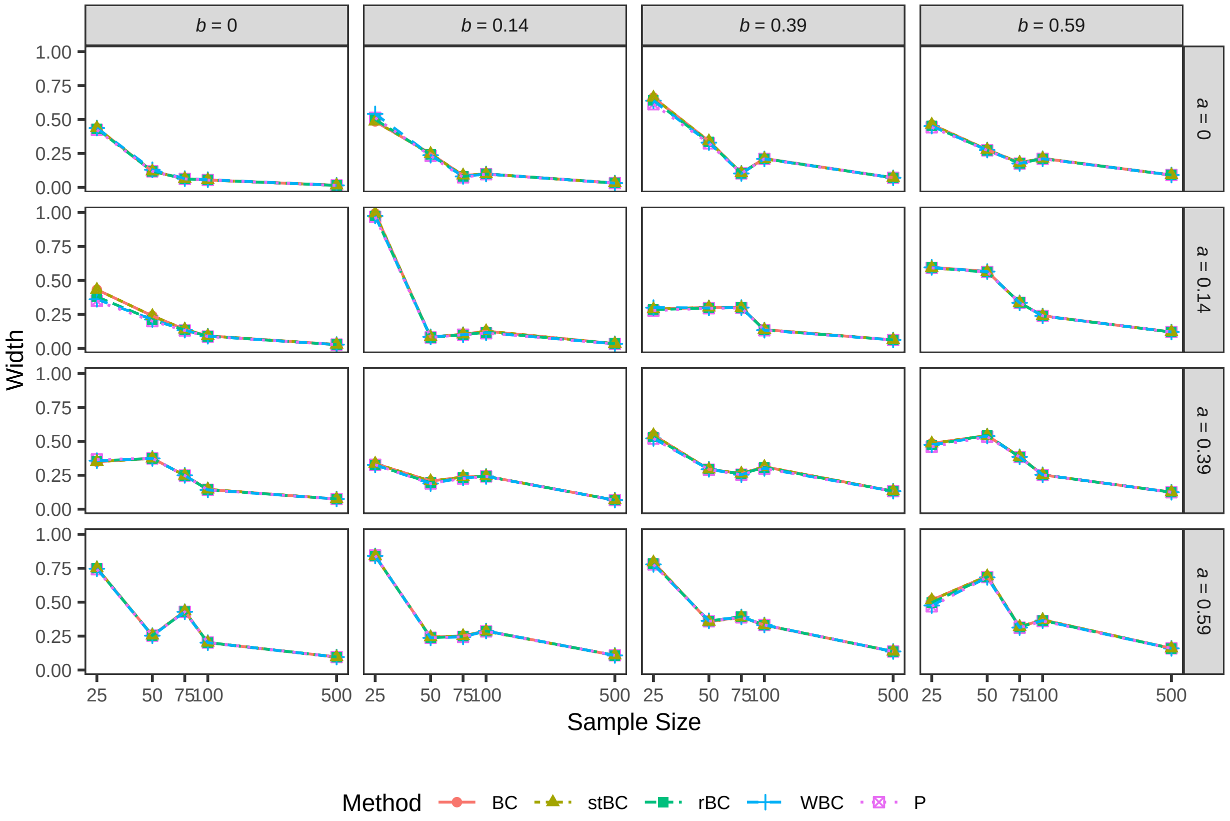

SM Figure 31. Width of all methods set at 95% confidence level across the range of a-path sizes, b-path sizes, and sample sizes. *Note.* 'BC' = bias-corrected bootstrap confidence interval, 'stBC' = significance-tested bias-corrected bootstrap confidence interval, 'rBC' = reduced bias-corrected bootstrap confidence interval, 'WBC' = 30% Winsorized bias-corrected bootstrap confidence interval, 'P' = percentile bootstrap confidence interval. The x-axis is on the natural log scale.

Confidence Level = 90%

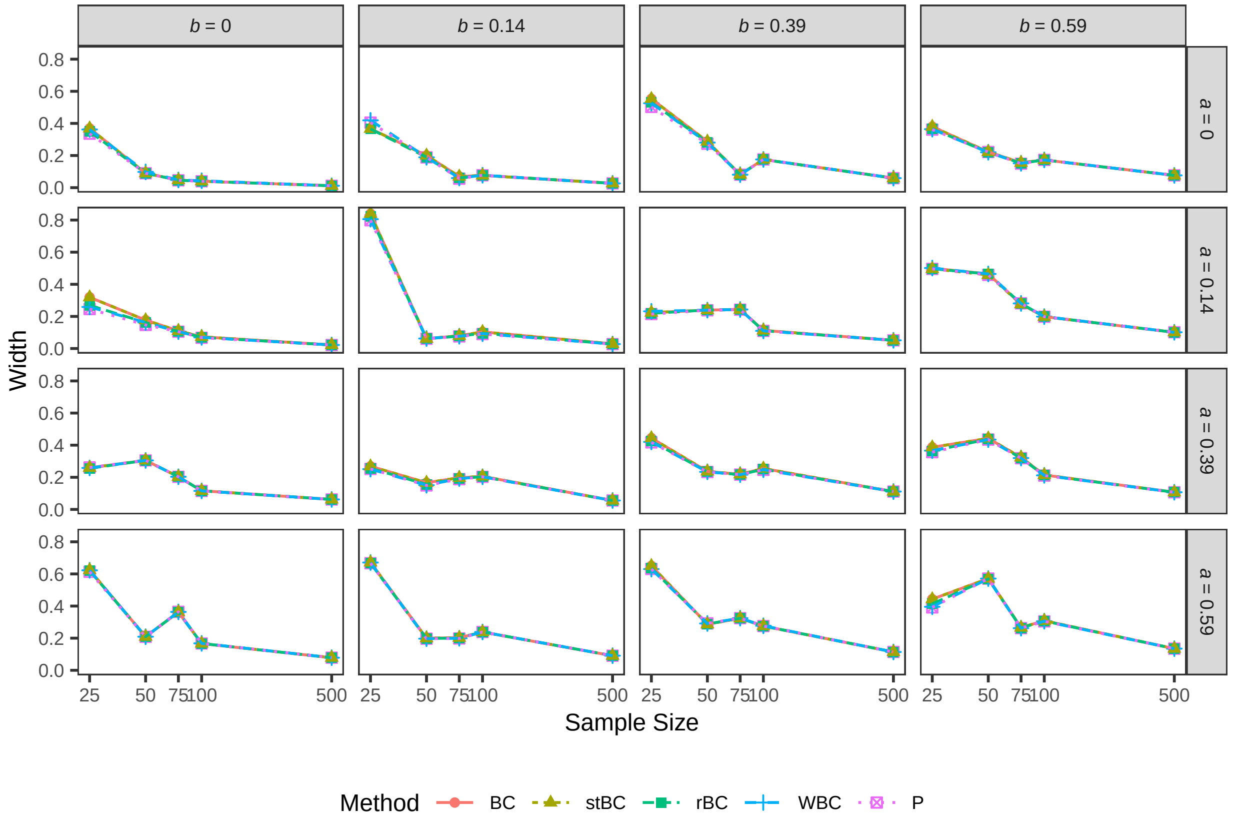

SM Figure 32. Width of all methods set at 90% confidence level across the range of a-path sizes, b-path sizes, and sample sizes. *Note.* 'BC' = bias-corrected bootstrap confidence interval, 'stBC' = significance-tested bias-corrected bootstrap confidence interval, 'rBC' = reduced bias-corrected bootstrap confidence interval, 'WBC' = 30% Winsorized bias-corrected bootstrap confidence interval, 'P' = percentile bootstrap confidence interval. The x-axis is on the natural log scale.

Confidence Level = 80%

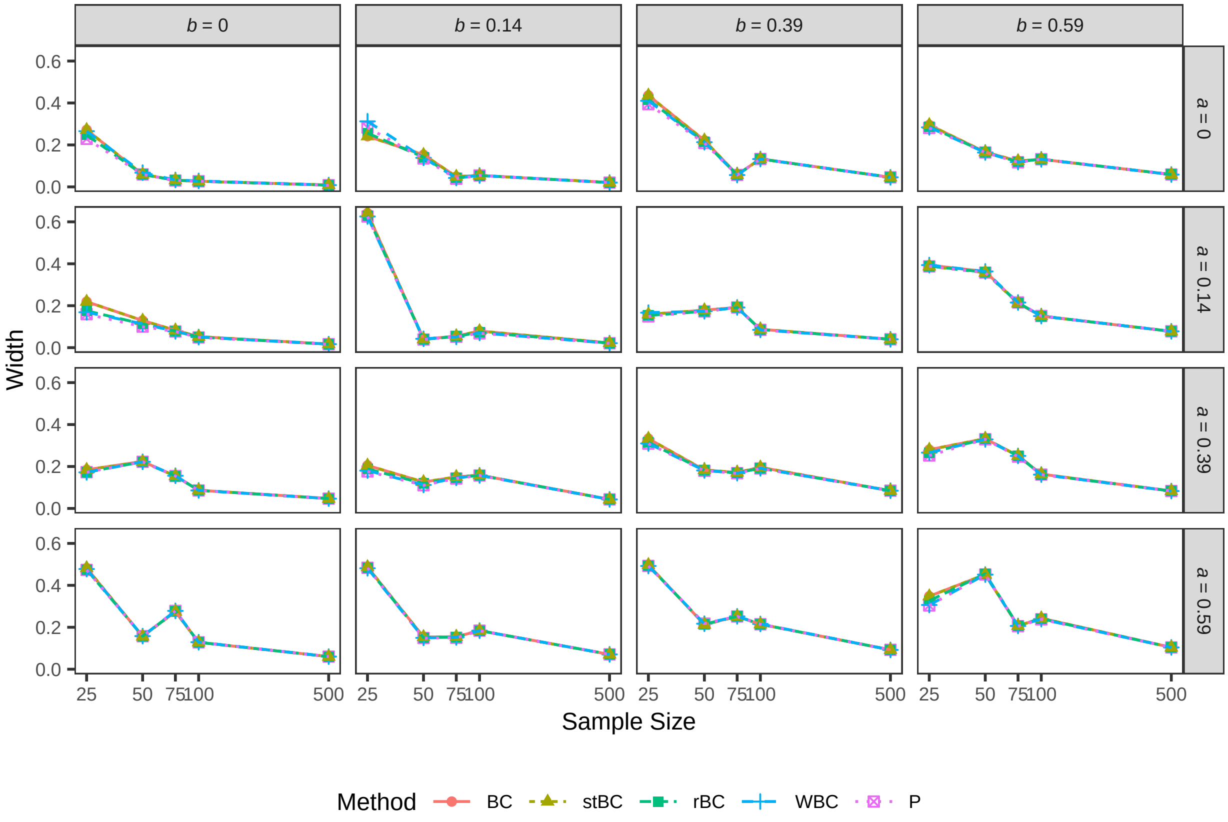

SM Figure 33. Width of all methods set at 80% confidence level across the range of a-path sizes, b-path sizes, and sample sizes. *Note.* ‘BC’ = bias-corrected bootstrap confidence interval, ‘stBC’ = significance-tested bias-corrected bootstrap confidence interval, ‘rBC’ = reduced bias-corrected bootstrap confidence interval, ‘WBC’ = 30% Winsorized bias-corrected bootstrap confidence interval, ‘P’ = percentile bootstrap confidence interval. The x-axis is on the natural log scale.

Rejection Rate Tables

Confidence Level = 95%

Factor	LR χ^2	df	p-value
a	127825	3	$< .001$
b	129185	3	$< .001$
Method	800	4	$< .001$
n	64392	4	$< .001$
$a \times b$	4844	9	$< .001$
$a\times$ Method	68	12	$< .001$
$a \times n$	8021	12	$< .001$
$b\times$ Method	56	12	$< .001$
$b \times n$	7884	12	$< .001$
Method $\times n$	97	16	$< .001$
$a \times b\times$ Method	48	36	.089
$a \times b \times n$	2460	36	$< .001$
$a\times$ Method $\times n$	8	48	1.00
$b\times$ Method $\times n$	9	48	1.00
$a \times b\times$ Method $\times n$	25	144	1.00

SM Table 1. ANOVA table for the results of the logistic regression model predicting rejection of null hypothesis (0 = No, 1 = Yes) with a- and b-path sizes, method used, sample size, and all possible interactions between these four factors. Type II sums of squares were used. *Note.* 'a' = a-path size, 'b' = b-path size, 'Method' = bootstrap confidence interval method, 'n' = sample size, 'LR χ^2' = χ^2 statistic for likelihood ratio test, 'df' = degrees of freedom.

	Estimate	Std. Error	z-value	p-value
(Intercept)	-8.267	0.035	-236.042	$< .001$
$a = 0.14$	2.804	0.021	130.808	$< .001$
$a = 0.39$	4.775	0.023	211.041	$< .001$
$a = 0.59$	5.254	0.023	226.621	$< .001$
$b = 0.14$	2.870	0.022	131.636	$< .001$
$b = 0.39$	4.849	0.023	210.874	$< .001$
$b = 0.59$	5.313	0.024	225.804	$< .001$
BCBCI	0.322	0.016	20.105	$< .001$
rBCBCI	0.153	0.016	9.521	$< .001$
stBCBCI	0.315	0.016	19.687	$< .001$
WBCBCI	0.060	0.016	3.716	$< .001$
$n = 25$	-2.207	0.017	-126.485	$< .001$
$n = 50$	-0.933	0.016	-58.760	$< .001$
$n = 500$	1.859	0.017	110.487	$< .001$
$n = 75$	-0.336	0.016	-21.611	$< .001$

SM Table 2. Table of regression coefficient estimates and their standard errors, z-values, and p-values from the logistic regression model predicting rejection of null hypothesis (0 = No, 1 = Yes) with a- and b-path sizes, Method used, and sample size (no interactions). *Note.* 'a' = a-path size, 'b' = b-path size, 'BCBI' = bias-corrected bootstrap confidence interval, 'stBCBI' = significance-tested bias-corrected bootstrap confidence interval, 'rBCBI' = reduced bias-corrected bootstrap confidence interval, 'WBCBI' = 30% Winsorized bias-corrected bootstrap confidence interval, 'n' = sample size. The reference group (intercept) corresponds to the percentile bootstrap confidence interval (PBCI) with $a = b = 0$ and $n = 100$.
